# Effects of chloride ions in acid-catalyzed biomass dehydration reactions in polar aprotic solvents

**DOI:** 10.1038/s41467-019-09090-4

**Published:** 2019-03-08

**Authors:** Max A. Mellmer, Chotitath Sanpitakseree, Benginur Demir, Kaiwen Ma, William A. Elliott, Peng Bai, Robert L. Johnson, Theodore W. Walker, Brent H. Shanks, Robert M. Rioux, Matthew Neurock, James A. Dumesic

**Affiliations:** 1 Department of Chemical and Biological Engineering, University of Wisconsin–Madison, Madison, WI 53706 USA; 2 DOE Great Lakes Bioenergy Research Center, University of Wisconsin–Madison, Madison, WI 53706 USA; 3 Department of Chemical Engineering and Materials Science, University of Minnesota, Minneapolis, MN 55455 USA; 4 Department of Chemical Engineering, Pennsylvania State University, State College, PA 16801 USA; 5 Department of Chemical and Biological Engineering, Iowa State University, Ames, IA 50011 USA; 6 Department of Chemistry, Pennsylvania State University, State College, PA 16801 USA

## Abstract

The use of polar aprotic solvents in acid-catalyzed biomass conversion reactions can lead to improved reaction rates and selectivities. We show that further increases in catalyst performance in polar aprotic solvents can be achieved through the addition of inorganic salts, specifically chlorides. Reaction kinetics studies of the Brønsted acid-catalyzed dehydration of fructose to hydroxymethylfurfural (HMF) show that the use of catalytic concentrations of chloride salts leads to a 10-fold increase in reactivity. Furthermore, increased HMF yields can be achieved using polar aprotic solvents mixed with chlorides. Ab initio molecular dynamics simulations (AIMD) show that highly localized negative charge on Cl^−^ allows the chloride anion to more readily approach and stabilize the oxocarbenium ion that forms and the deprotonation transition state. High concentrations of polar aprotic solvents form local hydrophilic environments near the reactive hydroxyl group which stabilize both the proton and chloride anions and promote the dehydration of fructose.

## Abstract (web summary)

Despite the potential advantages of using polar aprotic solvents for biomass upgrading reactions, fundamental understanding of these solvation effects is limited at present. Here, the authors show that further increases in catalyst performance in polar aprotic solvents can be achieved through the addition of inorganic salts.

## Introduction

Acid catalysis is ubiquitous in biomass-conversion processes to produce chemicals and fuels. It has been shown recently that the use of organic solvents for such biomass-upgrading reactions leads to increased catalytic activity and selectivity compared with reactions in aqueous media^1–25^. Accordingly, we explored the processing strategies using γ-valerolactone (GVL) solvent mixtures with water to produce concentrated streams of C_5_ and C_6_ sugars^2^ as well as valuable platform chemicals^3^ and high-value products^4^ from biomass using dilute concentrations of mineral acids. In subsequent studies on acid-catalyzed dehydration of xylose to furfural, we reported a 30-fold increase in reactivity and a 25% increase in selectivity (furfural yield increased from 50% to 75%) using GVL as a solvent compared with the reaction carried out in water^5^. Similar solvent enhancement effects have been reported using other polar aprotic solvents, such as tetrahydrofuran (THF)^6,7^, dimethyl sulfoxide^8–11^, 1,4-dioxane^12^, and sulfolane^13^. Despite the potential advantages of using polar aprotic solvents for biomass-upgrading reactions, fundamental understanding of these solvation effects in acid-catalyzed processes using nonaqueous solvents is limited at present.

Herein, we show that the addition of catalytic concentrations (e.g., < 5 mM) of inorganic salts, specifically chloride salts, in polar aprotic solvents, such as GVL, can further enhance the reactivity and yield for acid-catalyzed reactions related to biomass conversion. The dehydration of fructose to 5-hydroxymethylfurfural (HMF) is a Brønsted acid-catalyzed reaction, which is recognized as a promising biomass utilization platform. In addition, this extensively characterized reaction serves as a model system to study the mechanisms behind observed solvent effects. Based on fundamental reaction kinetics studies and ab initio molecular dynamics studies, we show that chloride ions participate in stabilizing protonated transition states for these acid-catalyzed reactions, leading to improvements in reaction rates and selectivities.

## Results

### Experimental reaction kinetics studies

Table 1 compares fructose conversion reaction rate constant values and HMF yield values for various strong homogeneous Brønsted acids (e.g., H_2_SO_4_, triflic acid, and HCl) in water and several polar aprotic solvents (containing 10 wt% water) at 373 K (rate constant values) and 393 K (yield values). Similar fructose conversion rate constant values were obtained in water (i.e., ~0.15 M^−1^ ks^−1^) regardless of the acid used, and HMF yields in water of 40% were achieved with both H_2_SO_4_ and HCl. Rate constant values increased by over an order of magnitude and HMF yields increased by 15% when GVL was used as the solvent with H_2_SO_4_ and triflic acid compared with using water as the solvent. Similar improvements in reactivity and HMF yields were also observed using dioxane and THF as solvents.

**Table 1:** Fructose conversion experimental results using homogeneous acid catalysts^a^

Solvent	Acid catalyst	Rate constant (M^−1^ ks^−1^)^b^	HMF yield (%)^c^
H_2_O	H_2_SO_4_	0.14 ± 0.01	38 ± 1
H_2_O	Triflic acid	0.15 ± 0.03	35 ± 2
H_2_O	HCl	0.14 ± 0.01	40 ± 1
90% GVL/10% H_2_O	H_2_SO_4_	20 ± 1	57 ± 1
90% GVL/10% H_2_O	Triflic acid	21 ± 4	56 ± 2
90% GVL/10% H_2_O	HCl	62 ± 4	78 ± 1
90% THF/10% H_2_O	H_2_SO_4_	5.8 ± 1	70 ± 1
90% THF/10% H_2_O	Triflic acid	5.6 ± 1	72 ± 5
90% THF/10% H_2_O	HCl	28 ± 5	76 ± 1
90% Dioxane/10% H_2_O	H_2_SO_4_	10 ± 2	64 ± 1
90% Dioxane/10% H_2_O	Triflic acid	7.7 ± 1	57 ± 2
90% Dioxane/10% H_2_O	HCl	95 ± 6	73 ± 1

^a^Reaction conditions: fructose (50 mM); acid (0.5 M for H_2_O; 5 mM for polar aprotic solvents); solvent (5 mL); stirring (700 rpm). ^b^Rate constant values at 373 K; *r* = *k* [R] [HB]. ^c^HMF yield values at 393 K at ~90% conversion. Bounds represent 95% confidence intervals

We observe a substantial difference in the fructose conversion rate constant values as well as HMF yield when HCl is used as the acid catalyst in these polar aprotic solvent systems compared with other strong acids. For example, in GVL with HCl, a rate constant value of 62 M^−1^ ks^−1^ and HMF yield of 80% were achieved, a threefold increase in rate constant value, and a 25% increase in HMF yield compared with using H_2_SO_4_ and triflic acid in GVL. Similar improvements using HCl compared with H_2_SO_4_ and triflic acid were observed in THF and dioxane solvents, with a 5-fold and 10-fold increase in rate, respectively. This increased reaction performance using HCl compared with other strong Brønsted acid catalysts is not observed using H_2_O as the solvent under these conditions.

As shown in Table 2, the improved performance of the fructose dehydration reaction can also be achieved with the addition of equimolar concentrations (i.e., 5 mM) of chloride-containing salts (e.g., KCl) with H_2_SO_4_ and triflic acid in GVL solvent, suggesting that ions are involved in the fructose dehydration catalytic process in polar aprotic solvents. The use of KCl for fructose dehydration without a Brønsted acid catalyst in GVL led to a low fructose conversion rate and shows that the chloride anion alone does not catalyze fructose dehydration.

**Table 2:** Fructose conversion experimental results in 90% GVL using homogeneous acid/salt systems^a^

Acid catalyst	Salt	Rate constant (M^−1^ ks^−1^)^b^
−^c^	KCl	3.2 ± 1
H_2_SO_4_	KCl	53 ± 9
Triflic acid	KCl	64 ± 5^e^
HCl	KCl	78 ± 8^f^
H_2_SO_4_	NaCl	55 ± 20
Triflic acid	NaCl	53 ± 6
Triflic acid	LiCl	69 ± 10
Triflic acid	CaCl_2_^d^	71 ± 10
HBr	−	28 ± 3
Triflic acid	KBr	22 ± 3
Triflic acid	NaBr	25 ± 2
Triflic acid	LiBr	29 ± 1
HI	−	2.7 ± 1
Triflic acid	KI	4.0 ± 1
Triflic acid	KF	1.9 ± 1

^a^Reaction conditions: fructose (50 mM); acid (5 mM); salt (5 mM); solvent (90% GVL/10% H_2_O; 5 mL); stirring (700 rpm). ^b^Rate constant values at 373 K; *r* = *k* [R] [HB]. ^c^No acid catalyst; *r* = *k* [R] [Salt]. ^d^Salt concentration (2.5 mM). ^e^78 ± 1% HMF yield at 393 K at ~90% conversion. ^f^81 ± 1% HMF yield at 393 K at ~90% conversion. Bounds represent 95% confidence intervals

We also explored the effects of adding various cations (Na^+^, Li^+^, and Ca^2+^) and anions (Br^−^, I^−^, and F^−^) using H_2_SO_4_ and/or triflic acid as catalysts in GVL (Table 2). The addition of the cations Na^+^, Li^+^, and Ca^2+^, and the anion Br^−^ with H_2_SO_4_ and/or triflic acid did not lead to significant differences in fructose conversion reaction rates. The addition of the anions I^−^ and F^−^ (with K^+^) led to lower reaction rates for fructose conversion reactions with triflic acid.

The iodide ion is strongly electronegative and may potentially promote a substitution reaction mechanism over an elimination mechanism, as no detectable production of HMF was observed using HI as a catalyst in GVL. Furthermore, iodide anions are strong reducing agents and have the potential to oxidize to diatomic iodine and water, deactivating the acid catalyst. The fluoride ion is more basic and has a strong affinity for the proton in solution relative to the other halogen ions used in this study (i.e., HF is a weaker acid; p*K*_a_ value in H_2_O of 3.2), and thus, F^−^ likely competes with fructose for the acidic proton, leading to decreased fructose dehydration rates.

Importantly, we found that the increased reactivity and yield with the addition of chloride ions in GVL is also observed for other acid-catalyzed sugar dehydration reactions, and this behavior thus appears to be of general significance (Supplementary Table 1). For example, the acid-catalyzed dehydration of xylose to furfural displays a similar increase in reactivity in GVL with HCl compared with H_2_SO_4_ (e.g., fourfold rate increase). Furthermore, the furfural yield from xylose increased from 60% using H_2_SO_4_ as a catalyst to 75% using HCl as a catalyst in GVL. Similarly, a twofold increase in glucose conversion reactivity and 25% HMF yield increase were achieved in GVL with HCl compared with H_2_SO_4_ as a catalyst, which has also been shown previously by Li et al.^26^.

## Discussion

Promotional effects in reactivity and selectivity have been reported in the literature for biomass conversion reactions using metal halides, such as NaCl, in aqueous media^27–30^. For example, Marcotullio et al. reported a 20% increase in furfural yield from xylose (i.e., from 60% to 80% furfural yield) and three- to fourfold increases in xylose dehydration reaction rates using 1 M NaCl in aqueous solution^27,28^. Similarly, Enslow et al. explored the role of various metal halides for xylose dehydration to furfural using salt cations (i.e., Li^+^, Na^+^, and K^+^) and anions (i.e., Cl^−^, Br^−^, and I^−^) at 5 M metal halide aqueous solutions, suggesting that metal halide cations disrupt the solvation of xylose by water, and metal halide anions act to stabilize critical xylose dehydration intermediates, leading to increased reactivity and selectivity^29^. In the present study, we observe promotional effects of inorganic salts in polar aprotic solvents at ~250- to 1000-fold lower concentrations compared with these chloride effects in water (e.g., 5 mM).

Mascal et al.^31,32^ have previously reported a method to produce chloromethylfurfural from carbohydrates (e.g., glucose) using HCl as a catalyst in a biphasic reaction system with concentrated HCl and dichloroethane, where Cl^−^ is directly involved in the reaction. Thus, we performed variable-temperature ^13^C NMR experiments for fructose conversion with ^13^C-enriched fructose, using both HCl and H_2_SO_4_ (0.05 M) in 90% dioxane-d_8_/10% H_2_O (w/w) at 340 K. Under these conditions, we did not detect any differences in the ^13^C NMR spectra between HCl and H_2_SO_4_-catalyzed reactions in dioxane-d_8_, suggesting that the observed reaction rate and product yield increases in the presence of chloride ions are not caused by changes in the reaction mechanism involving chlorinated species.

GVL has previously been shown to reversibly hydrate to 4-hydroxyvaleric acid (4-HVA) under acidic liquid-phase conditions^33^. Quantitative solution-phase ^1^H NMR was performed to display the effects of chloride ions on the 4-HVA equilibrium concentration in 90% GVL/10% H_2_O (w/w) with fructose and H_2_SO_4_ at various temperatures (Supplementary Figure 2a). Equilibrium concentrations of 4-HVA are marginally lower in the presence of KCl at typical reaction conditions (e.g., 1.2 vs. 1.4 mol% at 373 K). In addition, Supplementary Figure 2b displays the concentration of 4-HVA as a function of reaction time during fructose dehydration in 90 wt% GVL/10 wt% H_2_O (w/w) using H_2_SO_4_ with or without the presence of KCl. From the initial reaction start time to 10 min under reaction conditions (the period during which the reactor achieves the desired temperature of 373 K), the amount of 4-HVA in solution decreases slightly, and it then remains constant throughout the remainder of the experiment, indicating that GVL and 4-HVA are in equilibrium for both cases under typical reaction times and conditions. Moreover, reaction kinetics experiments were performed for fructose dehydration in 90% GVL/10% H_2_O containing H_2_SO_4_, KCl, and valeric acid (a molecular surrogate for 4-HVA at representative equilibrium concentrations of 1 mol% 4-HVA), as shown in Supplementary Figure 3. Negligible differences in fructose dehydration reaction kinetics were observed with the presence of valeric acid in the reactor. Thus, based on this analysis, we conclude that the increased reaction performance for fructose dehydration with chloride anions in GVL does not involve 4-HVA species.

The equilibrium of an acid, HB, in the liquid phase into an acidic proton, H^+^, and its conjugate base, B^−^, is written as

1 $${\mathrm{HB}} \hskip 2pt \rightleftarrows \hskip 2pt {\mathrm{H}}^ + + {\mathrm{B}}^ -$$

Acid catalysis of a reactant, R, in solution by H^+^ and an undissociated acid, HB, is often described by a reaction kinetics rate expression in the following form:

2 $$r = k_{{\mathrm{H}}^ + }\left[ {\mathrm{R}} \right][{\mathrm{H}}^ + ] + k_{{\mathrm{HB}}}[{\mathrm{R}}][{\mathrm{HB}}]$$

where *k*_H+_ and *k*_HB_ are the rate constants for the specific-acid catalyzed and general-acid catalyzed reactions, respectively. We have measured an inverse kinetic isotope effect (KIE) using D_2_O as a solvent, and also using GVL mixtures with D_2_O as a solvent, as shown in Supplementary Table 2. These measurements of the KIE led to rate constant value ratios (*k*_D_/*k*_H_) of 1.7−2.7. The measured KIE suggests that acid-catalyzed dehydration of fructose is catalyzed primarily by the acidic proton, H^+^ (i.e., acid dissociation is not rate determining), and therefore, general-acid catalysis (*k*_HB_) can be neglected^34,35^. Thus, the improved performance for the fructose dehydration reaction with the addition of chloride ions is not due to general-acid catalysis by undissociated HCl.

The equilibrium constant for dissociation of a Brønsted acid (Equation (1)) (i.e., a p*K*_a_ value) is dependent on the nature of the solvent^36^. Previously, we carried out reaction kinetics experiments for fructose conversion with triflic acid in GVL, THF, and dioxane with the addition of the conjugate base of the acid catalyst (i.e., potassium triflate)^37^. These reaction kinetics experiments for fructose conversion show that the rate is independent of the addition of the conjugate base of the acid catalyst, indicating that these strong acids are largely dissociated in each of these solvents. Thus, the measured rate allows calculation of the value of the specific acid rate constant (i.e., *k*_H+_).

We measured the apparent activation energies and pre-exponential factors for the specific acid-catalyzed fructose conversion in water using HCl and 90% GVL/10% H_2_O (w/w) with triflic acid and HCl. As shown in Supplementary Figure 1, the apparent activation energy values for water with HCl and for GVL with triflic acid are similar (e.g., ~110 kJ mol^−1^); however, the pre-exponential value is higher by an order of magnitude in the GVL solvent relative to water. The use of GVL as a solvent with HCl increases the apparent activation energy by 25 kJ mol^−1^ and increases the pre-exponential value by four orders of magnitude compared with using triflic acid in GVL.

Values of the enthalpy change for dissolution of fructose in H_2_O and 90% GVL/10% H_2_O with combinations of triflic acid and potassium salts were measured at 298 K (Supplementary Table 3) by solution calorimetry. These values show that a mixed solvent consisting of 90% GVL plus 10% H_2_O had a destabilizing effect on fructose relative to pure H_2_O (+ 5.2 kJ mol^−1^). The presence of 5 mM triflic acid in 90% GVL/10% H_2_O did not influence the stability of fructose relative to 90% GVL/10% H_2_O without an acid. Similarly, the presence of 5 mM KBr in 90% GVL/10% H_2_O did not have a significant effect on the enthalpy change for dissolution of fructose relative to 90% GVL/10% H_2_O without salt. In contrast, the addition of 5 mM KCl to 90% GVL/10% H_2_O increased fructose stability by 2.3 kJ mol^−1^ relative to 90% GVL/10% H_2_O without salt, which is in agreement with previous results showing the ability of chloride anions to stabilize carbohydrates in the gas phase^38,39^. However, the combined presence of 5 mM triflic acid and 5 mM KCl in 90% GVL/10% H_2_O negates the small stabilizing effect of 5 mM KCl on fructose in 90% GVL/10% H_2_O, resulting in no net stabilization. Thus, the calorimetric data suggest that the influence of the Cl^−^ is unlikely driven by enthalpic destabilization of reactants, but rather the promoting effect of Cl^−^ is either through stabilization of the transition state or by entropic contributions to the activation free energy.

**Fig. 1:**
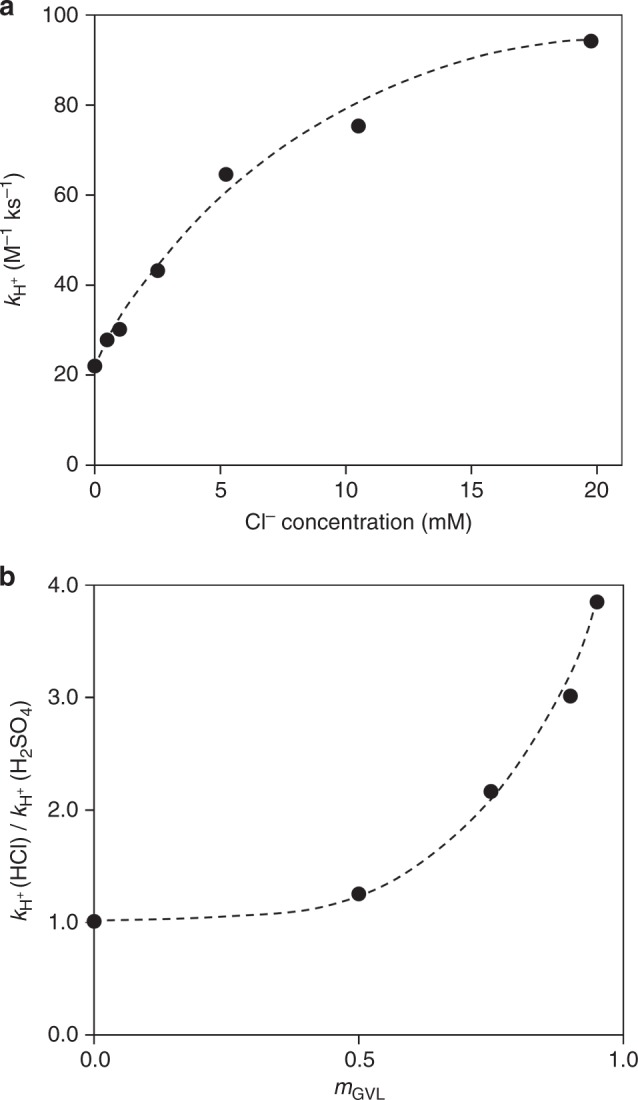
Fructose conversion rate constant versus Cl^−^ and GVL concentrations. **a** Fructose conversion rate constant values in 90% GVL/10% H_2_O with varying Cl^−^ concentration using triflic acid (5 mM acid) and (**b**) ratio of fructose conversion rate constant values for fructose conversion into HMF for HCl and H_2_SO_4_ (equimolar catalyst concentrations) with varying GVL solvent concentration (mass fraction with H_2_O). Black dashed lines represent visual guides. Reaction conditions: fructose (50 mM); acid (Fig. 1a: 5 mM; Fig. 1b: 5 mM–0.5 M), 373 K; solvent (5 mL); stirring (700 rpm). Reaction rate constant values: *r* = *k*_H+_ [R] [H^+^]

Figure 1a displays the results for the effects of varying the chloride ion concentration on the fructose conversion rate constant value using triflic acid as a catalyst in 90% GVL/10% H_2_O. The reaction rate constant shows an increasing, concave down reactivity trend with increasing chloride ion concentration, meaning there is a diminishing promotional effect using chloride ions. Furthermore, we explored the effect of varying the GVL solvent concentration on the enhancement in the rate caused by changing from H_2_SO_4_ to HCl (equimolar amounts) as the acid catalyst, plotted as the ratio of fructose conversion rate constant values for HCl and H_2_SO_4_ versus the mass fraction of GVL in solvents consisting of GVL mixed with water (Fig. 1b). The effect of chloride ions on the fructose conversion reaction rate relative to H_2_SO_4_ has an increasing trend with increasing concentrations of GVL in the solvent system.

Based on the aforementioned reaction kinetics results, we suggest that the rate-determining transition state, R^‡^, interacts with a Cl^−^ ion:

3 $${\mathrm{Cl}}^ - + R^\ddagger \mathop { \leftrightarrow }\limits^{K_{{\mathrm{Cl}}}} {\mathrm{RCl}}^\ddagger$$

where *K*_Cl_ is the equilibrium constant for the formation of a chloride transition state, RCl^‡^. The fraction of the transition state that is interacting with Cl^−^ is equal to [RCl^‡^]/[R^‡^]_total_, given by

4 $$\frac{{[{\mathrm{RCl}}^\ddagger ]}}{{[{\mathrm{R}}^\ddagger ]_{{\mathrm{total}}}}} = \frac{{K_{{\mathrm{Cl}}}[{\mathrm{Cl}}^ - ]}}{{1 + K_{{\mathrm{Cl}}}[{\mathrm{Cl}}^ - ]}}$$

where [R^‡^]_total_ is the total concentration of the transition state. The rate that proceeds through transition state R^‡^ is defined as *r*_o_, and the rate through the chloride transition state RCl^‡^ is equal to some faster value, *r*_Cl_. The total rate is now

5 $$\mathrm{rate} = r_{\mathrm{o}} + r_{{\mathrm{Cl}}}\left( {\frac{{K_{{\mathrm{Cl}}}[{\mathrm{Cl}}^ - ]}}{{1 + K_{{\mathrm{Cl}}}[{\mathrm{Cl}}^ - ]}}} \right)$$

Based on the results in Fig. 1b, the value of *K*_Cl_ increases as we add GVL to the solvent, in the same way that the value of the proton rate constant increases, i.e., the presence of GVL enhances the free energy for interaction of Cl^−^ with the transition state.

We can define an enhancement factor, *r*_enhance_, such that

6 $$r_{{\mathrm{enhance}}} = \frac{{r_{{\mathrm{Cl}}}}}{{r_{\mathrm{o}}}}$$

The rate becomes

7 $$\mathrm{rate} = r_{\mathrm{o}}\left[ {1 + r_{{\mathrm{enhance}}}\left( {\frac{{K_{{\mathrm{Cl}}}[{\mathrm{Cl}}^ - ]}}{{1 + K_{{\mathrm{Cl}}}[{\mathrm{Cl}}^ - ]}}} \right)} \right]$$

**Fig. 2:**
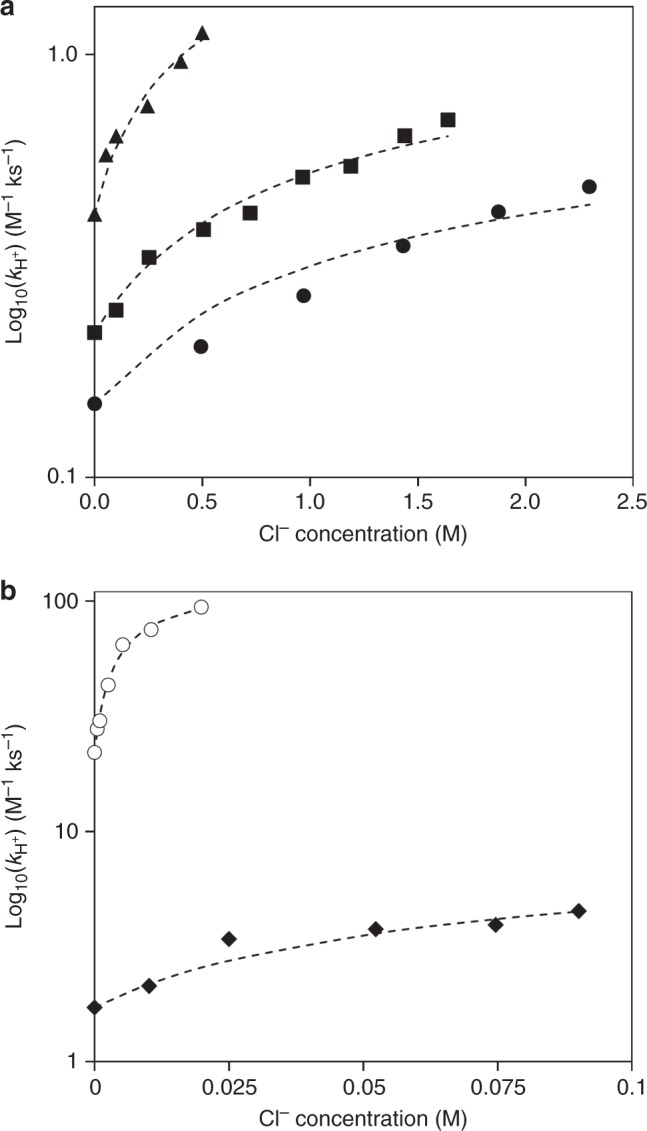
Fructose conversion rate constant versus Cl^−^ concentration in GVL/H_2_O (w/w). **a** Overall, 5% GVL (black circles), 25% GVL (squares), and 50% GVL (triangles). **b** In all, 75% GVL (diamonds) and 90% GVL (hollow circles). Black dashed lines represent the model fits using Equation (7). Reaction conditions: fructose (50 mM); acid (H_2_SO_4_; 5 mM–0.5 M); salt (KCl; 5 mM–2.5 M), 373 K; solvent (5 mL); stirring (700 rpm). Reaction rate constant values: *r* = *k*_H+_ [R] [H^+^]

**Fig. 3:**
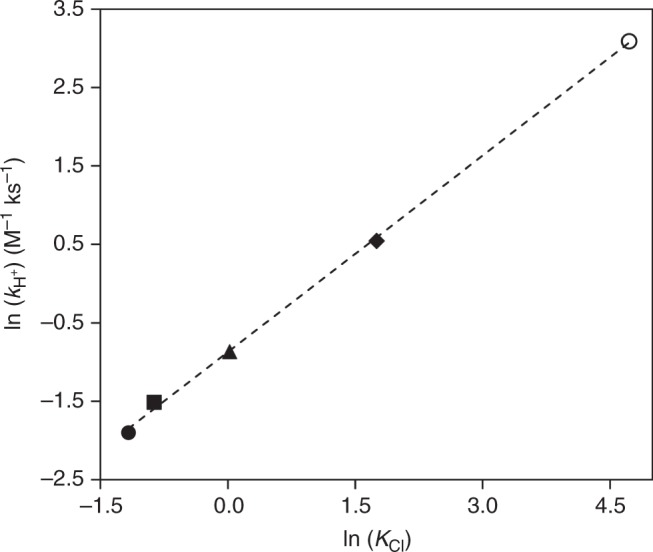
Fructose conversion rate constant versus *K*_Cl_ for Cl^−^ transition state formation. Data points represent the following GVL concentrations: 5% GVL (black circle), 25% GVL (square), 50% GVL (triangle), 75% GVL (diamond), and 90% GVL (hollow circle). The black dashed lines represent a linear trendline: y = 0.84 × −0.87, R^2^ = 0.99. Reaction rate constant values: *r* = *k*_H+_ [R] [H^+^]. *K*_Cl_ represents the equilibrium constant. Equilibrium constant values (i.e., *K*_Cl_): model fits using Equation (7) with data from Fig. 2. Reaction conditions: fructose (50 mM); acid (H_2_SO_4_; 5 mM–0.5 M); salt (KCl; 5 mM–2.5 M), 373 K; solvent (5 mL); stirring (700 rpm)

To probe the behavior predicted by Equation (7), we collected reaction kinetics data for fructose conversion using H_2_SO_4_ and varying amounts of KCl for a variety of solvent mixtures consisting of GVL and water (i.e., 5% GVL, 25% GVL, 50% GVL, 75% GVL, and 90% GVL). Figure 2 displays rate constant values plotted against the chloride ion concentration for each solvent mixture. Equation (7) was used to model these reaction kinetics data in various GVL/H_2_O solvent systems, using *r*_enhance_ and *K*_Cl_ as parameters. This analysis shows that the value of *r*_enhance_ is a constant (equal to ~5) for all GVL concentrations, and the values of *r*_o_ and *K*_Cl_ depend on the solvent concentration. Figure 3 shows a plot of the value of the chloride ion-free fructose conversion rate constant values (i.e., *k*_H+_) versus the modeled equilibrium constants for the formation of the chloride transition state (i.e., *K*_Cl_) for each GVL/H_2_O solvent system. This plot shows that the enhancements of both the rate of acid-catalyzed conversion of fructose as well as the *K*_Cl_ have a linear dependence with increasing concentrations of GVL solvent.

Previously, we suggested that increased reactivity in polar aprotic solvents, such as GVL, for acid-catalyzed reactions is due to destabilization of the acidic proton, where the protonated transition state is destabilized by a fraction of this amount in these polar aprotic solvents relative to water^5^. More recently, we quantified these solvation effects in terms of initial and transition state contributions^37,40^ and developed a multi-descriptor correlation model that predicts rate constants as a function of solvent composition^41^ for acid-catalyzed reactions. Based on the experimental results of the present study, we now probe whether the observed increases in acid-catalyzed dehydration rates with chloride salts in polar aprotic solvents are due to the stabilization of the protonated transition state by chloride ions.

Ab initio density functional theory molecular dynamic simulations were used to explore the effects of GVL, as well as the influence of the chloride ion and other anions on the kinetics for the acid-catalyzed dehydration of fructose. The Brønsted-acid catalyzed conversion of fructose to HMF involves multiple dehydration steps. The initial dehydration of the hydroxyl group at the anomeric C2 carbon is considered to be the rate-determining step^37,42,43^. This step proceeds via the initial protonation and elimination of water from the C2 hydroxyl of fructose (Supplementary Figure 4a; Structures a–c). The resulting oxocarbenium intermediate, which is stabilized by the lone pair of electrons on the ring oxygen, is subsequently deprotonated by the basic oxygen of a water molecule in solution, resulting in the formation of the enol (Supplementary Figure 4a; Structures d–f). The initial proton transfer from solution to fructose, proton addition and the elimination of water at the C2 position of fructose, and the deprotonation of the corresponding oxocarbenium ion are illustrated in the structures shown in Supplementary Figure 5.

**Fig. 4:**
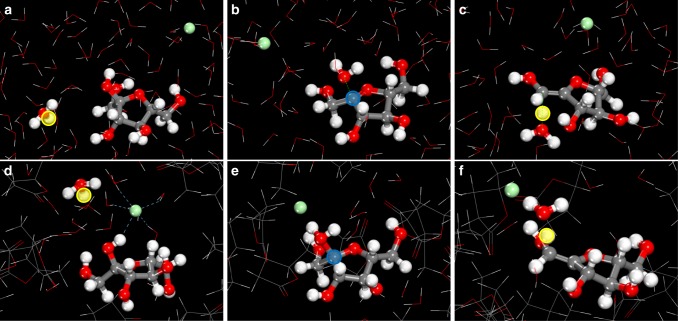
Fructose dehydration reaction structures. Structures along the reaction path for fructose dehydration carried out in water and HCl mixtures (**a**–**c**) and in 90% GVL/10% H_2_O (w/w) and HCl mixtures (**d**–**f**). The structure in the first (**a**, **d**), second (**b**, **e**), and third (**c**, **f**) columns refers to fructose along with H^+^ and Cl^−^ in their reactant state in solution, the resulting oxocarbenium ion from the initial protonation of the C2 hydroxyl group and the elimination of water, and the transition state for the deprotonation of the oxocarbenium ion to form the enol product, respectively. Red, gray, green, and white spheres refer to the oxygen, carbon, chlorine, and hydrogen atoms, respectively. The yellow and blue highlighted circles refer to the reactive proton and oxocarbenium ion centers, respectively (additional structures along the path are presented in Supplementary Figure 6 and Supplementary Figure 9)

The calculated free-energy changes for acid-catalyzed fructose dehydration in H_2_O and GVL solvent systems with various anions are summarized in Table 3. For fructose dehydration in pure H_2_O (Supplementary Figure 6 and Supplementary Figure 7), the reaction free energy for the initial protonation of the C2 hydroxyl of fructose and the formation of the oxocarbenium ion is 58 kJ mol^−1^. The activation free energy for the subsequent proton abstraction step from the oxocarbenium ion to form the enol intermediate (Fig. 4c) was calculated to be 35 kJ mol^−1^. Thus, the apparent barrier which involves the free energy to form the oxocarbenium ion and the intrinsic activation free energy to subsequently deprotonate the ion is calculated to be 93 kJ mol^−1^. The simulations demonstrate that H^+^ and Cl^−^ ions formed upon dissociation of the acid in H_2_O are strongly bound to water molecules within extensive hydrogen-bonding networks (Supplementary Figure 8) and are significantly separated from one another. These solvation structures prevent interactions between the chloride anion and the reactive site on fructose. The anion, therefore, does not influence the calculated barriers and overall energies. This behavior is consistent with the experimental results, which shows that chloride ions and other anions have little effect on the rates or the yields for fructose dehydration reactions carried out in water at low ion concentrations. At higher concentrations of salt, such as those reported in previous studies^27–30^, chloride ions reside near the alcohol, promoting the acid-catalyzed dehydration.

**Table 3:** AIMD-calculated reaction free energies for fructose dehydration

Solvent system	Catalyst/anion	Oxocarbenium ion formation^a^ (kJ mol^−1^)	Proton abstraction^‡b^ (kJ mol^−1^)	Total free energy of activation^‡c^ (kJ mol^−1^)
H_2_O	H^+^	57	35	92
H_2_O	H^+^/Cl^−^	58	35	93
75% GVL/25% H_2_O	H^+^	49	35	84
75% GVL/25% H_2_O	H^+^/Cl^−^	44	30	74
90% GVL/10% H_2_O	H^+^	43	33	76
90% GVL/10% H_2_O	H^+^/Cl^−^	38^d^	29	67
90% GVL/10% H_2_O	H^+^/TfO^−^	43^e^	33^e^	76
90% GVL/10% H_2_O	H^+^/HSO_4_^−^	45	33	78

^a^The values for oxocarbenium ion formation are the reaction free energies for the simultaneous protonation of the C2 hydroxyl of fructose and the elimination of water that forms. ^b^The proton abstraction values refer to the activation free energies for water to deprotonate the oxocarbenium ion that forms. ^c^The apparent activation barrier listed as the total activation free energy is the sum of the free energy of the reaction to form the oxocarbenium ion and the activation free energy to deprotonate it. ^d^The free energy of oxocarbenium ion formation in 90% GVL/10% H_2_O (w/w) with HCl required a correction calculated from the 75% GVL/25% H_2_O (w/w) system to ensure the complete dissociation of the H^+^ and Cl^−^ in the initial reactant state as discussed in the Supplementary Discussion. ^e^The triflate anion migrated away from fructose and local water molecules during the free energy sampling and partitioned between the water and GVL domains away from the active site. The numbers reported in this column were therefore taken from the 90% GVL/10% H_2_O (w/w) system without an anion. The double dagger (‡) symbol is a descriptor that refers to the transition state

Higher concentrations of GVL in water disrupt hydrogen-bonding networks and lead to the formation of hydrophilic domains near the fructose that are surrounded by hydrophobic GVL domains^37^. The encapsulated hydrophilic domains localize the protons near the fructose, thus removing the energetic penalties required to transfer the protons from bulk water. In addition, GVL-encapsulated water stabilizes the charged transition state for deprotonation, thus lowering the overall activation barrier. Simulations carried out for solvent mixtures consisting of 75% GVL/25% H_2_O and 90% GVL/10% H_2_O (Supplementary Figure 9a–c) in the absence of an anion show that the overall apparent activation free energies for fructose dehydration decrease to 84 kJ mol^−1^ and to 76 kJ mol^−1^, respectively. These results are in alignment with the experimentally observed increased fructose dehydration reaction rates with increasing GVL concentrations (Fig. 2; chloride ion-free results).

**Fig. 5:**
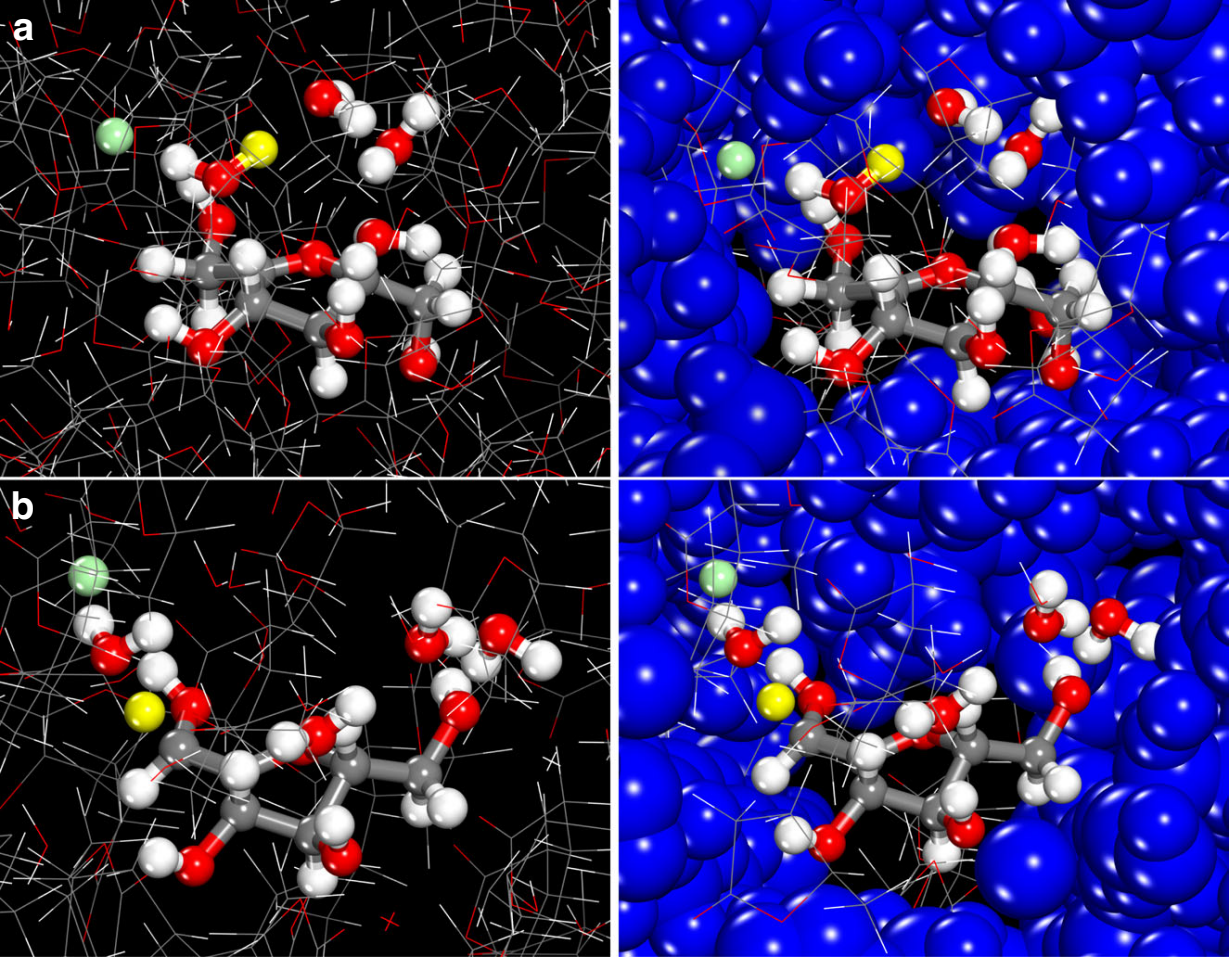
Structures representing solvation by Cl^−^ of transition states in 90% GVL/10% H_2_O (w/w). **a** The protonation of the C2 hydroxyl and the elimination of water to form the oxocarbenium ion and (**b**) the deprotonation of the oxocarbenium ion to form the enol. The Cl^−^ (green sphere) in both transition state structures directly interacts with the active protons (yellow sphere) and resides in the hydrophilic shell that surrounds fructose. The active hydrophilic cavity which is shown via the ball-and-stick structures is encapsulated by the hydrophobic shell comprising GVL. The structures on the left-hand side show the hydrophobic outer shell via simple stick figures, whereas those on the right-hand side depict the atoms in the hydrophobic shell as CPK structures to better highlight the inner hydrophilic cavity and the outer hydrophobic shell. The high concentration of GVL localizes the H^+^ and Cl^−^ ions at the active site, enabling Cl^−^ to promote the reaction. The structures of transition states for fructose dehydration carried out in 90% GVL/10% H_2_O (w/w)

The simulation results show that increasing GVL concentration localizes the Cl^−^ anion within the hydrophilic domain near fructose (Supplementary Figure 10 and Supplementary Figure 11). For 75 wt% and 90 wt% GVL solvent mixtures with water, the chloride ion preferentially resides in the local hydrophilic water domain near the C2 hydroxyl of fructose, thus enabling Cl^−^ to stabilize both the formation of the oxocarbenium ion intermediate (Fig. 4d, e) as well as the transition state for deprotonation (Fig. 4f), whereas in the pure water system, the Cl^−^ preferentially resides in bulk water (Fig. 4a–c). In the 75% GVL/25% H_2_O (w/w) system with Cl^−^, the overall activation free energy is 74 kJ mol^−1^, which is 10 kJ mol^−1^ lower than the value for the chloride ion-free system. The results from the simulations for the 90% GVL/10% H_2_O (w/w) solvent mixture containing a chloride ion (Supplementary Figure 9d–f and Supplementary Figure 12) show that increasing GVL from 75 wt% to 90 wt% GVL decreased the barrier by another 7 kJ mol^−1^, resulting in an overall barrier of 67 kJ mol^−1^. The high concentration of GVL in this system results in large hydrophobic domains taken up by the GVL molecules and small hydrophilic domains that localize near the hydroxyl groups on fructose. This behavior increases the number of active H^+^ and Cl^−^ ions near the reactive hydroxyl groups, and in addition, allows Cl^−^ to stabilize both the oxocarbenium ion and deprotonation transition states (Fig. 5).

Simulations carried out in the 90% GVL/10% H_2_O (w/w) system using triflic acid and sulfuric acid (Supplementary Figure 9) for fructose dehydration gave apparent activation energies of 76 kJ mol^−1^ and 78 kJ mol^−1^, respectively, showing minimal change from the barrier of 74 kJ mol^−1^ in the absence of the Cl^−^ anion in 90% GVL/10% H_2_O. Both triflic acid (p*K*_a_ = −15)^44^ and sulfuric acid (p*K*_a_ = −10; Supplementary Table 4)^45,46^ are more acidic than HCl (p*K*_a_ = −6; Supplementary Table 4)^44^. Relative to the Cl^−^ anion, the triflate (CF_3_SO_3_^−^) and bisulfate (HSO_4_^−^) anions delocalize the negative charge over their corresponding structures and are therefore more stable (i.e., weaker conjugate bases), leading to weaker interactions with the charged oxocarbenium-ion intermediate and the transition state for deprotonation of fructose dehydration. In simulations with triflic acid in 90% GVL/10% H_2_O, the CF_3_SO_3_^−^ anion migrates away from fructose and resides instead at the interface of the water–GVL domains, where the CF_3_ group interacts with the hydrophobic GVL domain and the SO_3_^−^ group resides in the hydrophilic water domain (Supplementary Figure 13). This behavior inhibits the interaction of the CF_3_SO_3_^−^ anion with the oxocarbenium-ion intermediate or the charged transition state, and therefore offers little stabilization. The changes in the calculated activation free energies found in changing the acid or the solvents used in carrying out the dehydration are in agreement with those trends determined by the changes in the experimental rate constants, as is shown in Supplementary Figure 14. While chloride ions increase the rate of conversion of fructose into HMF, they also increase the overall reaction selectivity and yield of HMF as they do not promote the subsequent C–C bond activation that controls the conversion of HMF into levulinic acid^37^. Full simulation details can be found in the Supplementary Methods, Supplementary Discussion, and Supplementary Figures 4–20.

The effects of chloride ions in polar aprotic solvents for fructose dehydration to HMF can also be achieved using heterogeneous Brønsted acids as catalysts (Supplementary Table 5). Rate constant values for fructose conversion in the presence of chloride ions were measured by adding benzyltriethylammonium chloride (BTEAC) to propylsulfonic acid-functionalized silica (PSA/SiO_2_) and Amberlyst 70 catalysts in 90% GVL/10% H_2_O. The benzyltriethylammonium cation was used to provide sufficient steric hindrance to prevent cation exchange with the acidic protons of the solid catalysts. The addition of chloride ions led to only slight improvements in fructose conversion rate constant values for these heterogeneous catalysts. However, increased HMF yields (80%) from fructose were obtained with the addition of BTEAC for both PSA/SiO_2_ and Amberlyst 70, an increase of ~10% HMF yield compared with the chloride ion-free GVL.

In summary, we have studied the effects of salts in polar aprotic solvents, such as GVL, on acid-catalyzed biomass dehydration reactions. Significant increases in reactivity (e.g., 10-fold) and product selectivities (> 80% yields) were observed for the acid-catalyzed dehydration of fructose to HMF with the addition of catalytic concentrations (e.g., 5 mM) of chloride salts in polar aprotic solvents. Based on reaction kinetics results and ab initio molecular dynamics simulations for a variety of strong homogenous acid catalysts and salts in polar aprotic solvent mixtures with water, we show that chloride ions aid in the stabilization of protonated transition states for these acid-catalyzed reactions.

## Methods

### Reaction kinetics experiments

Reaction kinetics measurements were carried out in closed thick-walled glass batch reactors (10 mL). In a typical experiment, 5 -mL solutions of a 50 mM reactant (e.g., fructose), 5 mM acid (e.g., HCl), and 5 mM salt (e.g., KCl) in an organic solvent mixed with water (e.g., 25 wt% GVL with 75 wt% H_2_O) were added into closed-batch reactors. The reactors were placed in an oil bath and stirred at 700 rpm with magnetic stir bars at reaction temperature. The reactors were removed at specific reaction times, and the reactions were stopped by cooling the reactors in an ice bath at 273 K. Sample analyses were performed using a high-performance liquid chromatograph (Waters Alliance 2695) instrument equipped with a differential refractometer (Waters 410) and a photodiode array detector (Waters 996). Concentrations of fructose (RID), HMF (UV; 320 nm), and levulinic acid (RID) in liquid solution were monitored using an ion-exclusion column (Bio-Rad; Aminex HPX-87H; 7.8 × 300 mm, 5 μm). As an example, a table showing a subset of reactions performed with experimental conditions for the Brønsted acid-catalyzed fructose dehydration is presented in Supplementary Table 6.

Reaction kinetics profiles for the reactant conversion were constructed from the aforementioned reaction kinetics data. Each reaction time represents an individual experiment, and these values were combined to produce reaction kinetics profiles. Values of rate constants (*k*_H+_; Equation (2)), reaction energetics values (*A* and *E*_a_), and equilibrium constants (*K*_Cl_) were derived from the reaction kinetics profiles for reactant consumption using nonlinear least-squares regression in MATLAB (nlinfit function; Levenberg–Marquardt nonlinear least-squares algorithm). Confidence intervals were calculated at the 95% confidence level (nlparci function). Detailed experimental methods can be found in the Supplementary Methods.

### NMR spectroscopy

For detection and analysis of chloride-containing species, ^13^C NMR experiments were performed using a Bruker spectrometer equipped with a 14.1 T superconducting magnet and acquired and processed using TOPSPIN. The samples were prepared in a glovebox and kept on ice until analysis. NMR tubes with sealed screw caps purchased from Wilmad Glass were used for all spectra. ^1^H spectra were acquired using a recycle delay of 3.0 s with 30° ^1^H excitation pulse lengths. ^13^C spectra were acquired by using a recycle delay of 5.0 s with 30° ^13^C excitation pulse lengths using a carrier frequency offset of 100 ppm.

Quantitative solution-phase ^1^H NMR experiments of GVL and 4-HVA (Supplementary Figure 2) were performed on a Bruker AV 500-MHz spectrometer equipped with an N_2_-cooled cryogenic probe. To avoid any confounding effects on the equilibrium conversion of GVL into 4-HVA, samples did not contain deuterated solvents or internal standards. Spectra were collected with the deuterium lock channel off, and shimming was performed manually on the residual water resonance at ~3.5 ppm. Quantitative ^1^H spectra were collected using a standard 45° pulse sequence with a pulse delay of 10 s, an acquisition time of 2 s, and eight scans per spectrum. The relative values of the peak integrals at ~0.5 and ~0.7ppm, corresponding to 4-HVA and GVL, respectively, were used to assess the mole fraction of 4-HVA in solution as a function of temperature and reaction time. Samples generated under reaction conditions were stored at 277 K overnight. For the variable-temperature experiments, samples were allowed to equilibrate for 30 min after the sample and probe reached the desired temperature.

### Solution calorimetry

Solution calorimetry experiments were performed on a semi-adiabatic solution calorimeter (TAMIII Precision Solution Calorimeter, TA Instruments) with the bath temperature controlled to ± 0.0001 K. A glass ampoule was loaded with fructose through a small opening in the ampoule. The opening was closed with a rubber stopper and sealed with wax. The ampoule was immersed in a 25 -mL vessel containing solvent and inserted into the calorimeter. The contents of the vessel were stirred at 600 rpm to ensure proper dissolution of fructose and proper heat transfer. Dissolution of the fructose was initiated by breaking the top and bottom of the ampoule on a spike within the vessel, exposing the contents of the ampoule to the solvent. The change in temperature was measured with SolCal v1.2 software. Internal calibrations were performed before and after the dissolution process by charging 3 J of heat into the vessel and measuring the change in temperature. The heat of the dissolution process was determined using the two calibrations. To account for the heat associated with solvent–ampoule interactions, the heat associated with breaking an empty ampoule in the solvent was subtracted from the observed heat. The adjusted heat was then normalized by the number of moles of fructose.

### Computational simulations

Liquid-phase molecular systems were prepared by placing a fructose molecule, solvent molecules, a proton, and an anion in a 15 × 15 × 15 Å^3^ periodic box. The molecular configurations of each system were equilibrated using classical molecular dynamics implemented in GROMACS^47^ software. The final snapshots were taken as starting structures for ab initio molecular dynamics (AIMD) simulations based on density functional theory (DFT) quantum chemical calculations in CP2K/Quickstep package^48^. The classical molecular dynamics simulations of the prepared molecular systems were equilibrated for 1 ns using a canonical (NVT) ensemble followed by 10 ns of equilibration using an isothermal–isobaric (NPT) ensemble. The temperature was kept at 373 K for all simulations. Molecular interactions of fructose and GVL molecules were adapted from an all-atom version of Optimized Potentials for Liquid Simulations^49,50^ (OPLS-AA) force field. Literature force fields were implemented for the hydronium ion^51^, chloride anion^52^, bisulfate anion^53^, triflate anion^54^, and water^55^.

In DFT-based AIMD simulations, all systems were further equilibrated for 5 ps. Non-local exchange and correlation energies were calculated using the Perdew–Burke–Ernzerhof^56^ (PBE) functional. Goedecker–Teter–Hutter pseudopotentials^57^ were used to represent the electronic density with a plane-wave cutoff of 280 Ry. A short-ranged version of Gaussian-type double-ζ basis set^58^ was used to expand the Kohn–Sham orbitals. DFT-D3(BJ) dispersion corrections^59^ were applied to correct for the medium- and long-range van der Waals interactions. The temperature of the systems was kept at 373 K. Equations of motion were integrated with a time step of 0.5 fs. Deuterium mass was used in place of hydrogen atoms to attenuate the fast vibration modes associated with light particles. The results of free energy sampling as well as complete details on the computational methods can be found in the Supplementary Methods, Supplementary Discussion, and Supplementary Figures 4–20. Extensive molecular dynamics simulations were carried out to establish the lowest free energy states. Subsequent AIMD simulations were carried out with established sampling methods. Errors in the calculated changes in the reaction energies and activation barriers were found to be within 2 kJ mol^−1^.

## Supplementary information

Supplementary Information

Peer Review File

## Data Availability

The data that support the plots within this paper and other findings of this study are available from the corresponding author upon reasonable request.
