# Supplementary material for: Effects of chloride ions in acid-catalyzed biomass dehydration reactions in polar aprotic solvents
Source: Nat Commun. 2019 Mar 8;10:1132. doi: 10.1038/s41467-019-09090-4 (PMC6408490; doi:10.1038/s41467-019-09090-4)
Supplement: Supplementary file 1 — Supplementary Information [file 41467_2019_9090_MOESM1_ESM.pdf]

Supplementary Information for

Effects of Chloride Ions in Acid-Catalyzed Biomass Dehydration Reactions in Polar Aprotic Solvents

Authors:

Max A. Mellmer[1,2], Chotitath Sanpitakseree[3], Benginur Demir[1,2], Kaiwen Ma[1], William A. Elliott[4], Peng Bai[3], Robert L. Johnson[5], Theodore W. Walker[1], Brent H. Shanks[5], Robert M. Rioux[4,6], Matthew Neurock[3], James A. Dumesic[1,2,*]

Affiliations:

[1]Department of Chemical and Biological Engineering, University of Wisconsin–Madison, Madison, WI 53706, USA

[2]DOE Great Lakes Bioenergy Research Center, University of Wisconsin–Madison, Madison, WI 53706, USA

[3]Department of Chemical Engineering and Materials Science, University of Minnesota, Minneapolis, MN 55455, USA

[4]Department of Chemical Engineering, Pennsylvania State University, State College, PA 16801, USA

[5]Department of Chemical and Biological Engineering, Iowa State University, Ames, IA 50011, USA.

[6]Department of Chemical Engineering, Pennsylvania State University, State College, PA 16801, USA

[*]Corresponding Author: jdumesic@wisc.edu

Table of Contents:

Supplementary Methods

Supplementary Discussion

Supplementary Tables 1-6

Supplementary Figures 1-20

Supplementary References 1-35

Supplementary Methods

For reaction kinetics experiments, chemicals were obtained from Sigma-Aldrich, including reactants (fructose, glucose, and xylose) and products (HMF, furfural, and levulinic acid), inorganic salts (KCl, NaCl, LiCl, $CaCl_2$, KBr, NaBr, LiBr, KI, KF, K-triflate), solvents (dioxane, THF, GVL, D_2O, and 1,4-dioxane-d_8), and acid catalysts (H_2SO_4, triflic acid, HCl, HBr, HI) were used as acquired. Purified water was used and obtained through an in-house Milli-Q water purification system. Amberlyst 70 (The Dow Chemical Company; acid site density = 2.55 mmol g^{-1}) was washed, dried, and crushed before use. Si-propylsulfonic acid was obtained from SiliCycle (SiliaBond Propylsulfonic Acid SCX-2; acid site density = 0.63 mmol g^{-1}) and was used as acquired.

Reaction kinetics measurements were carried out in closed thick-walled glass batch reactors (10 mL) for fructose, xylose, and glucose dehydration in dioxane, THF, GVL, H_2O, D_2O, 1,4-dioxane-d_8 and mixtures thereof. Reaction kinetics data for fructose dehydration in pure organic solvents without water could not be obtained due to low fructose solubility. In a typical experiment, 5 mL solutions of 50 mM reactant (e.g., fructose), 5 mM acid (e.g., HCl), and 5 mM salt (e.g., KCl) in an organic solvent mixed with water (e.g., 25 wt% GVL with 75 wt% H_2O) were added into closed batch reactors. The reactors were placed in an oil bath and stirred at 700 rpm with magnetic stir bars at reaction temperature. The reactors were removed at specific reaction times, and the reactions were stopped by cooling the reactors in an ice bath at 273 K.

After each reaction, the content of the reactor was filtered using a 0.2 μm membrane (VWR International; PTFE). Sample analyses were performed using a high-performance liquid chromatograph (Waters Alliance 2695) instrument equipped with a differential refractometer (Waters 410) and a photodiode array detector (Waters 996). Concentrations of fructose (RID), HMF (UV; 320 nm), levulinic acid (RID) in liquid solution were monitored using an ion-exclusion column (Bio-Rad; Aminex HPX-87H; 7.8 × 300 mm, 5 μm). A mobile phase of 5 mM sulfuric acid aqueous solution at a flow rate of 0.6 mL min^{-1} was used.

Reaction kinetics profiles for the reactant conversion were constructed from the aforementioned reaction kinetics data. As an example, a table showing a subset of reactions performed including experimental conditions for the Brønsted acid-catalyzed fructose dehydration is presented in Supplementary Table 6. Each reaction time represents an individual experiment, and these values were combined to produce reaction kinetics profiles. Values of rate constants

(k_{H+}; Manuscript Equation (2)), reaction energetics values (A and E_a), and equilibrium constants (K_{Cl}) were derived from the reaction kinetics profiles for reactant consumption using nonlinear least squares regression in MATLAB (nlinfit function; Levenberg-Marquardt nonlinear least squares algorithm). Confidence intervals were calculated at the 95% confidence level (nlparci function). Fructose dehydration has been shown in previous studies to be first order with respect to both reactant and proton concentration[1], and small quantities of levulinic acid and formic acid products were also detected in these reactions.

For computational simulations, the liquid-phase systems with fructose were initially prepared by placing fructose, solvent molecules, a proton, and an anion in a 15 × 15 × 15 $Å^3$ periodic box using Packmol tool[2]. The molecular configurations for fructose dehydration in different solvent systems were equilibrated using classical molecular dynamics as implemented in GROMACS software package[3]. The subsequent equilibration and *ab initio* density functional theory (DFT)-based molecular dynamics simulations of these systems were carried out using CP2K program[4].

The water system consisted of 100 H_2O molecules, one hydronium ion, and one fructose molecule. The water system with free chloride also included one chloride anion in solution. In the system where the chloride anion was artificially placed near fructose, the chloride ion was bound to the C2 hydroxyl group of fructose, resulting in a single fructose-Cl^- entity. An unconstrained molecular dynamics simulation was carried out to establish the radial distribution function and determine the distance between the anion and hydrogen on the fructose hydroxyl group (2.1 Å). The 75% GVL/25% H_2O (w/w) system consisted of 13 GVL molecules, 23 H_2O molecules, one hydronium ion, and one fructose molecule. The 75% GVL/25% H_2O (w/w) system with chloride anion consisted of 13 GVL molecules, 23 H_2O molecules, one hydronium ion, and one fructose-Cl^- structure. The 90% GVL/10% H_2O (w/w) system consisted of 15 GVL molecules, 10 H_2O molecules, one hydronium ion, and one fructose molecule. The 90% GVL/10% H_2O (w/w) system with a chloride anion, bisulfate anion, or triflate anion each consisted of 15 GVL molecules, 10 H_2O molecules, one hydronium ion, and one fructose-anion structure. The distances from the bisulfate sulfur atom and triflate sulfur atom to the hydrogen atom on the C2 fructose hydroxyl group were set to 3.0 Å and 2.9 Å, respectively.

The prepared periodic molecular systems were equilibrated in GROMACS for 1 ns using constant number of particles, constant volume, and constant temperature (NVT) ensembles followed by 10 ns of equilibration using constant number of particles, constant pressure, and

constant temperature (NPT) ensembles. The temperature was kept near 373 K using the Leap-frog stochastic dynamics integrator[5] with a 2 ps temperature coupling time constant. The pressure of the system was controlled by the Parrinello–Rahman barostat[6,7] with a 2 ps coupling time constant. The molecular interactions of fructose and GVL molecules were adapted from the all-atom version of Optimized Potentials for Liquid Simulations (OPLS-AA)[8,9] force field. Force fields for the hydronium ion[10], chloride anion[11], bisulfate anion[12], and triflate anion[13] were adopted from the literature. The interactions of water molecules were taken from an Extended Simple Point Charge (SPC/E) water model[14]. The SETTLE algorithm[15] was used to constrain the bond angle and bond distance in water molecules. The cut-off value for intermolecular interactions was set at 7 Å. The Particle Mesh Ewald (PME) scheme was used to account for the long-range electrostatic interactions.

The final atomic coordinates and the average lattice sizes were taken as the initial configurations for the periodic *ab initio* molecular dynamics simulations (AIMD). All systems were further equilibrated for 5 ps using AIMD without any constraints. The exchange and correlation energies were calculated within the generalized gradient approximation using Perdew–Burke–Ernzerhof (PBE) functional[16]. Goedecker–Teter–Hutter pseudopotentials[17] were used to represent the electronic density with a plane wave cut-off of 280 Ry. A short-ranged version of Gaussian-type double-ζ basis set[18] was used to expand the Kohn-Sham orbitals. DFT-D3(BJ) dispersion corrections[19] were applied to correct for the medium and long range van der Waals interactions. The neutralizing background charge was assumed by CP2K. The temperature of the systems was kept at 373 K using canonical sampling through a velocity rescaling (CSVR) thermostat[20]. A time step of 0.5 fs was used, and all the hydrogen mass was replaced with deuterium to attenuate the fast vibration associated with light hydrogen atoms.

Ab initio molecular dynamics were performed using an umbrella sampling technique to calculate the reaction free energy and activation free energy. The umbrella samplings were carried out under the same conditions as the unbiased AIMD runs. A minimum of 16 sampling windows evenly distributed over the reaction coordinate were used to ensure a reasonable overlapping of sampled configurations between adjacent windows. For each sampling window, the condensed systems were further equilibrated for 5 picoseconds followed by 15 picoseconds of data collection. The shapes of free energy profiles were obtained using Weight Histogram Analysis Method (WHAM)[21]. All the free energy profiles are presented in Supplementary Figures 15-20.

The free energy calculations of fructose dehydration were performed on the carbenium ion formation step (Supplementary Figure 4, a; Structures a-c) and proton abstraction step (Supplementary Figure 4, a; Structures d-f). The reaction coordinates used were defined based on coordination numbers:

$$\mathrm{CN}_{ij} = \frac{1-\left(d_{ij}/c\right)^{9}}{1-\left(d_{ij}/c\right)^{14}} \tag{1}$$

where CN_{ij} is the coordination number of atom *i* with respect to atom *j*, d_{ij} is the distance between atom *i* and *j* in angstroms, and *c* is the parameter that controls the shape of Supplementary Equation 1. The *c* parameter was set to 1.3 for coordination numbers involving the C-H as well as O-H bonds and 1.6 for the longer C-O bond.

The reaction coordinate for the carbenium ion formation step (Supplementary Figure 4, b) was defined as:

$$reaction\ coordinate = CN_{O-2H} - CN_{C-all\ O} \tag{2}$$

where $CN_{O\text{-}2H}$ is a sum of the coordination numbers of fructose hydroxyl oxygen with respect to hydroxyl hydrogen and a closest water hydrogen. The $CN_{C\text{-}allO}$ is a sum of the coordination numbers of fructose C2 carbon with respect to all oxygen atoms and other anions in the solvent, including the oxygens on the triflate and bisulfate anions and the chloride anion. This reaction coordinate describes a simultaneous protonation of the C2-OH group along with C-O bond cleavage. The sampling on this coordinate ranged from −0.1 to 1.8 with a spring constant for bias harmonic potential of 0.3 Hartree. The bias potential, *V*, takes the harmonic form of:

$$V = K \times \left(CV - CV_{target}\right)^{2} \tag{3}$$

where *K* is the harmonic spring constant, *CV* is the instantaneous reaction coordinate described in Supplementary Equation 2, and CV_{target} is the center of bias harmonic potential.

The reaction coordinate for the proton abstraction step (Supplementary Figure 4, c) is defined as follows:

$$reaction\ coordinate = CN_{H-O} - CN_{C-H} \tag{4}$$

where $CN_{C\text{-}H}$ is the coordination number of C1 fructose carbon to the cleaving proton, and $CN_{H\text{-}O}$ is the coordination number of the cleaving proton with respect to an oxygen atom of a water molecule. The water molecule associated with the $CN_{H\text{-}O}$ term is selected using the following function:

$$d_{H-O} = \frac{\sum_{i \in O_{all}} d_{H-i} \, exp(200 \times CN_{H-i})}{\sum_{i \in O_{all}} exp(200 \times CN_{H-i})} \quad (5)$$

where $d_{H\text{-}O}$ is the distance from the cleaving proton to the oxygen of the closest water molecule.

The O_{all} is a set of all solvent oxygen and chloride atoms including the oxygens on triflate and bisulfate anions as well as the Cl of the chloride anion. The parameter *c* used in *CN* calculations was set to 1.8 for the Supplementary Equation 5. Each term in the numerator of Supplementary Equation 5 is relatively small except the term for the closest water molecule. When the denominator is applied, the term with the largest $d_{H\text{-}i}$ remains relevant while the other terms with smaller $d_{H\text{-}i}$ become negligible. Supplementary Equation 5 results in the distance between the cleaving proton to the closest oxygen atom of a water molecule, including oxygens on anions and the chloride anion. This distance, $d_{H\text{-}O\ water}$, is used in the calculation of $CN_{H\text{-}O\ water}$ in Supplementary Equation 4. Overall, the second reaction coordinate describes a proton transfer from the fructose oxocarbenium ion to the closest water molecule that acts as a base, thus, involving both O-H bond breaking and O-H bond formation. The sampling along this coordinate ranged from −0.85 to 0.95 with a spring constant for bias harmonic potential of 0.45 Hartree. The reaction coordinates for the proton abstraction (Supplementary Equation 4 and Supplementary Equation 5) were also used in conjunction with a potential wall on the reaction coordinates for carbenium ion formation (second term of Supplementary Equation 2) to deter the carbenium ion from turning back to initial state. The position of the potential wall was determined from the fluctuation of the reaction coordinate Supplementary Equation 2 in the window corresponding to the carbenium ion, where the wall was set at the maximum reaction coordinate observed in the simulation.

Supplementary Discussion

The rates for Brønsted acid-catalyzed reactions carried out in the liquid phase are governed by the stability of the transition state of the rate-limiting step relative to the stability of the solvated reactants.[22,23] As discussed in the manuscript, the acids used herein fully dissociate in water as well as the co-solvent systems.[24] As such, classical molecular dynamics simulations were carried out to determine the equilibrium configurations of fructose, dissociated proton, and anion in the different solvent systems. The lowest energy structures were used as the initial configurations for *ab initio* density functional (DFT) molecular dynamics simulations with umbrella sampling to

determine the free energies of activation and the reaction free energies for the different elementary steps in the dehydration of fructose.

The Brønsted-acid catalyzed conversion of fructose to HMF and other platform chemicals involve multiple dehydration steps, where the first dehydration is identified as the rate-limiting step.[1,25] This step proceeds by the initial protonation of fructose at the C2 hydroxyl group (Supplementary Figure 4, a; Structures a-b) followed by the elimination of water (Supplementary Figure 4, a; Structures b-c). The resulting oxocarbenium structure is stabilized by a lone pair of electrons on the ring oxygen. The oxocarbenium ion is subsequently deprotonated by the basic oxygen of a water molecule in the solvent, thus resulting in the formation of the olefin structure (Supplementary Figure 4, a; Structures d-f). The initial transfer of the proton from solution to fructose, proton addition, the elimination of water at the C2 position of fructose, and the deprotonation of the corresponding oxocarbenium ion are illustrated in the structures shown in Supplementary Figure 5.

Ab initio molecular dynamics simulations were carried out for each of the elementary steps to calculate intrinsic activation energies and reaction energies as well as the overall activation free energy for fructose dehydration in presence of chloride, triflate, and bisulfate anions that result from the dissociation of hydrochloric acid, triflic acid, and sulfuric acid, respectively. For comparison purposes we also carried out simulations with and without the corresponding anions. The simulations of fructose dehydration with and without a chloride anion were performed in pure water, 75% GVL/25% H_2O (w/w), and 90% GVL/10% H_2O (w/w), while the simulations with triflate and bisulfate anions were performed in 90% GVL/10% H_2O (w/w).

In the pure water system with no anion, the activation free energy and the reaction free energy for the protonation of fructose and the simultaneous water cleavage (Supplementary Figure 6, a-b) were calculated to be 65 kJ mol^{-1} and 57 kJ mol^{-1}, respectively. The activation free energy of the following proton abstraction step (Supplementary Figure 6, b-c) was found to be 35 kJ mol^{-1}. The overall apparent activation free energy is 92 kJ mol^{-1} for the first dehydration step. The subsequent transformation of the olefin intermediate into hydroxymethylfurfural (HMF) is likely fast and therefore not considered.[1,25] We combined the free energy of reaction for the carbenium ion formation step with the activation free energy of proton abstraction step. The resulting value is reported as an overall apparent activation free energy of the reaction, summarized in Table 3 of the manuscript.

In the 75% GVL/25% H_2O (w/w) system, the reaction free energy of the oxocarbenium ion formation of fructose and the activation free energy of subsequent proton abstraction were calculated to be 49 kJ mol^{-1} and 35 kJ mol^{-1}, respectively. The total apparent activation free energy of 84 kJ mol^{-1} is 8 kJ mol^{-1} lower than the reaction in water without an anion. This computational result agrees with the higher experimentally measured fructose conversion reaction rates in GVL/H_2O systems. This enhancement in reactivity in GVL is in part the result of the destabilization of reactant proton, since GVL molecules disrupts the hydrogen bond network.[24] Furthermore, the fructose structure contains multiple hydrophilic hydroxyl groups, which promotes the localization of the hydrophilic proton around the active site which stabilizes the transition state, as outlined in our previous work.[24] The results in 90% GVL/10% H_2O (w/w) system (Supplementary Figure 9, a-c) are consistent with the experimental fructose dehydration reactivity trend. In this case, the overall activation free energy of fructose dehydration was calculated to be 76 kJ mol^{-1}, which was 8 kJ mol^{-1} and 16 kJ mol^{-1} lower than the activation energies in 75% GVL/25% H_2O (w/w) and pure water systems, respectively.

The force-field molecular dynamics simulations were performed prior to the *ab initio* molecular dynamics simulations to examine the solvation structure of the chloride anion in the water system. The results show extensive solvation of the anion in the hydrogen bond network between water molecules (Supplementary Figure 6, d-f). This solvation structure inhibits any significant interactions between the chloride anion and the reactive site on fructose. The reaction free energy of oxocarbenium ion formation of fructose in water with a chloride anion (Supplementary Figure 6, d-f and Supplementary Figure 7, a) was calculated to be 58 kJ mol^{-1}, which is similar to the 57 kJ mol^{-1} with no chloride anion.

The effect of the chloride anion on the overall activation free energy of fructose dehydration could be studied if the chloride anion is arranged near the fructose hydroxyl group at the beginning of the *ab initio* simulations (Supplementary Figure 6, g-i and Supplementary Figure 7, b). Relatively short *ab initio* molecular dynamics simulations (20 ps in total) allow the chloride anion to stay near fructose, whereas a longer simulation time will eventually result in the migration of chloride anion into bulk water. At short simulation times, the reaction free energy of carbenium ion formation was calculated to be 50 kJ mol^{-1}, which is 7 kJ mol^{-1} lower than the system without anion. This result suggests the direct interaction between anion and the reactive species plays a major role in the experimentally observed reaction rate enhancement in presence of anions. The

chloride anion, initially located at the fructose hydroxyl group, becomes fully solvated in bulk water solvent at the end of carbenium formation step (Supplementary Figure 6, e). Therefore, the chloride anion does not participate in the subsequent proton abstraction step. This behavior is supported by the calculated 35 kJ mol^{-1} activation free energy of the proton abstraction in the system which has chloride ion initially located on the fructose in the reactant state. The overall apparent activation energy of this artificial system then becomes 85 kJ mol^{-1}.

In the 75% GVL/25% H_2O (w/w) and 90% GVL/10% H_2O (w/w) systems, the simulations were carried out on the fructose structure with chloride anion positioned near the hydroxyl group. The calculated reaction free energy of carbenium ion formation and the activation free energy of subsequent proton abstraction in 75% GVL/25% H_2O system were 44 kJ mol^{-1} and 30 kJ mol^{-1}, respectively. The resulting overall activation free energy of 74 kJ mol^{-1} is 10 kJ mol^{-1} lower than the 75% GVL/25% H_2O system without chloride anion. The transition state complex formed during the proton abstraction step in 75% GVL/25% H_2O featured a chloride ion near the reactive complex (Supplementary Figure 10, c), contrary to the transition state in the water system (Supplementary Figure 6, h). This chloride interaction is made possible by the diminishing available phase space, which is largely taken up by GVL molecules. This behavior allows the negatively charged chloride anion to be located near the transition state structure and contributes to the stabilization of the positively charged transition state (Supplementary Figure 11).

The simulations in 90% GVL/10% H_2O (w/w) (Supplementary Figure 9, d-f) resulted in 55 kJ mol^{-1} and 29 kJ mol^{-1} for the free energy of carbenium ion formation and the activation free energy of proton abstraction, respectively. The overall activation free energy of 84 kJ mol^{-1} for fructose dehydration contradicts the experimental results that the addition of chloride anion positively affects the overall fructose conversion rate. Detailed inspection revealed that the hydrochloric acid did not fully dissociate in the simulations (Supplementary Figure 12, a). The use of a small box size in the simulations led to a high acid concentration (~ 0.5 M) with few water molecules present, while a much lower acid concentration (e.g., 5 mM) was used in the fructose dehydration experiments. Therefore, the higher overall activation free energy is a result of computational artifact that requires a correction. The correction for the results in 90% GVL/10% H_2O (w/w) was taken from the results in 75% GVL/25% H_2O (w/w) system, where the chloride anion lowers the reaction free energy of carbenium ion formation from 49 kJ mol^{-1} to 44 kJ mol^{-1}. The correction factor of 5 kJ mol^{-1} is then subtracted from the 43 kJ mol^{-1} reaction free energy of carbenium ion

formation in 90% GVL/10% H_2O without an anion. This correction leads to values of 38 kJ mol^{-1} and 29 kJ mol^{-1} for the free energy of carbenium ion formation and the activation free energy of proton abstraction, respectively. The overall activation free energy of fructose conversion in 90% GVL/10% H_2O becomes 67 kJ mol^{-1}, which is 9 kJ mol^{-1} lower than the reaction in 90% GVL/10% H_2O without anion. This result is in agreement with the experimentally observed higher fructose conversion rates with the addition of chloride anion in 90% GVL/10% H_2O, and indicates the enhanced dehydration reactivity is due to the anion-stabilized positively charged transition state structure.

Analogous to the simulations with the chloride anion, *ab initio* molecular dynamics simulations were carried out in 90% GVL/10% H_2O (w/w) using fructose structures with triflate and bisulfate anions positioned near the fructose hydroxyl groups. For simulations with bisulfate ion (Supplementary Figure 9, g-i), the reaction free energy of carbenium ion formation of fructose and the activation free energy of proton abstraction were calculated to be 45 kJ mol^{-1} and 35 kJ mol^{-1}, respectively. The total overall activation energy of 78 kJ mol^{-1} is comparable to the 76 kJ mol^{-1} from the reaction in 90% GVL/10% H_2O (w/w) without an anion. This result implies that the bisulfate anion interacts with reactive species along the reaction pathway similarly to other solvent molecules. In addition, the sulfuric acid fully dissociated under the simulating condition since it did not yield an acid-base ion pairing as observed with hydrochloric acid. This observation is consistent with the higher acidity of sulfuric acid than hydrochloric acid (see Supplementary Table 4).

Unlike simulations with the chloride and bisulfate anions, simulations involving the triflate anion were unique in that the migration of the triflate anion away from the fructose took place within a few ps in the simulation (Supplementary Figure 9, j-k and Supplementary Figure 13). This migration event appears to be the result of the electronic structure of the triflate ion where the negative charge is largely attracted toward the trifluoromethyl group. This results in low electron density on the triflate oxygen atoms which consequently attenuates the extent of hydrogen bonding with other species in the system. The migration of weak-hydrogen bonding species away from the hydrophilic domain near fructose thus becomes favorable. Therefore, the overall activation free energy of fructose dehydration in 90% GVL/10% H_2O (w/w) with triflic acid was determined to be the same as the overall activation free energy in the 90% GVL/10% H_2O system without anion, 76 kJ mol^{-1}.

The dissociation of Brønsted acid in aqueous solution can be described using Born-Haber cycle as follows:

$$\begin{array}{ccccc} \text{H-B}_{(g)} & \xrightarrow{\Delta G_{(g)}} & \text{H}^{+}_{(g)} & + & \text{B}^{-}_{(g)} \\ \Delta G_{(sol,\ H\text{-}B)} \downarrow & & \Delta G_{(sol,\ H^{+})} \downarrow & & \Delta G_{(sol,\ B^{-})} \downarrow \\ \text{H-B}_{(aq)} & \xrightarrow{\Delta G_{(aq)}} & \text{H}^{+}_{(aq)} & + & \text{B}^{-}_{(aq)} \end{array} \qquad (6)$$

where B is the conjugate base of the H-B acid and $\Delta G_{(g)}$ and $\Delta G_{(aq)}$ are acid dissociation free energies in gas and aqueous phase, respectively. The $\Delta G_{(sol)}$ terms represent the solvation free energies. The comparison of acidity was carried out using pK_a values calculated by:

$$pK_a = -\frac{\Delta G_{(aq)}}{RT\ln(10)} \qquad (7)$$

where $\Delta G_{(aq)}$ is the free energy for dissociation of the acid in the aqueous phase.

The value of $\Delta G_{(aq)}$ for each acid was calculated from the following equation:

$$\Delta G_{(aq)} = -\Delta G_{(sol,H-B)} + \Delta G_{(g)} + \Delta G_{(sol,H^{+})} + \Delta G_{(sol,B^{-})} - 7.93\ [\text{kJ mol}^{-1}] \qquad (8)$$

where $\Delta G_{(sol,\ H\text{-}B)}$ is determined from DFT simulations as the difference in the free energy of the acid in the solution compared to the free energy of the acid in the gas phase, whereas the values for $\Delta G_{(g)}$, $\Delta G_{(sol,\ H^{+})}$, and $\Delta G_{(sol,\ B^{-})}$ were taken from the literature values[26-28], as shown in Supplementary Table 4. The final term, 7.93 kJ mol^{-1}, is associated with the change in standard concentration from 1 atm in gas phase to 1 mol L^{-1} in aqueous phase. DFT calculations were carried out using Gaussian 09 software using M06-2X[29] DFT hybrid functional and 6-311+g(d,p) basis set. The solvation free energies were obtained using SMD[30] implicit solvation model.

The relationship between the proton activity coefficient and the reactivity can be established by writing the rate in terms of the activities which are equal to the activity coefficients multiplied by the concentrations of the reactants.

$$r = k_{H^{+}}\gamma_{H^{+}}\gamma_{R}[H^{+}][R] \qquad (9)$$

where r is an experimentally measured rate, k_{H+} is a rate constant for specific-acid catalyzed and γ_{H+} and γ_R are the activity coefficient of proton and fructose, respectively. Transition state theory can then be used to express the rate constants in term of activation free energy:

$$r = \kappa \frac{k_B T}{h} e^{\frac{-\Delta G^{\ddagger}}{RT}} \gamma_{H^+} \gamma_R [H^+][R] \tag{10}$$

where κ is a transmission coefficient, k_B is Boltzmann constant, T is temperature, and h is Planck constant. The activity coefficients γ_{H+} and γ_R can then be combined with the activation free energy term:

$$r = \kappa \frac{k_B T}{h} e^{\frac{\ln(\gamma_{H^+})RT + \ln(\gamma_R)RT - \Delta G^{\ddagger}}{RT}} [H^+][R] \tag{11}$$

to express rate in terms of an apparent free energy barrier:

$$r = \kappa \frac{k_B T}{h} e^{\frac{\Delta G^{\ddagger}_{calculated}}{RT}} [H^+][R] \tag{12}$$

As such the experimental as well as the calculated activation free energies reported in the manuscript due include these changes in proton activity. The simulations model the proton and transition state interactions with the explicit solvent molecules in the local environment and as such directly calculate the explicit changes in proton activities.

Supplementary Tables

Supplementary Table 1. Sugar conversion reaction rate constant values and yield values in 90% GVL/10% H_2O using homogeneous acid catalysts.[a]

Sugar	Acid	Rate Constant (M^{-1} ks^{-1})[b]	HMF or Furfural Yield (%)[c]
Glucose	H_2SO_4	23	20
Glucose	HCl	41	45
Xylose	H_2SO_4	25	60
Xylose	HCl	95	74

[a] Reaction conditions: sugar (25 mM for glucose; 50 mM for xylose); acid (10 mM); solvent (90% GVL/10% H_2O; 5 mL); 433 K; stirring (700 rpm). [b] $r = k$ [R] [HB]. [c] HMF (for glucose dehydration) and furfural (for xylose dehydration) yield values at approximately 90% conversion.

Supplementary Table 2. Solvent kinetic isotope effect for fructose dehydration to HMF.[a]

Solvents	Acid / Salt	k_D/k_H[b]
D_2O/H_2O	H_2SO_4	1.9
D_2O/H_2O	HCl	2.4
10%D_2O/10%H_2O in 90% GVL	H_2SO_4	1.7
10%D_2O/10%H_2O in 90% GVL	HCl	2.7
10%D_2O/10%H_2O in 90% GVL	H_2SO_4 / KCl	2.4

[a] Reaction conditions: fructose (50 mM); acid (0.5 M for H_2O/D_2O; 5 mM for GVL with H_2O/D_2O); salt (5 mM); 373 K; solvent (5 mL); stirring (700 rpm). [b] $r = k_H$ [R] [HB] using H_2O; $r = k_D$ [R] [HB] using D_2O.

Supplementary Table 3. Enthalpy changes for dissolution of fructose in solvent mixtures containing combinations of potassium salts and triflic acid.[a]

Solvent	Acid	Salt	ΔH_{sol} (kJ mol^{-1})	95% C.I. (kJ mol^{-1})
H_2O	−	−	8.9	[8.5, 9.3]
90% GVL/10% H_2O	−	−	14.1	[13.5, 14.7]
90% GVL/10% H_2O	−	KCl	11.8	[11.2, 12.4]
90% GVL/10% H_2O	−	KBr	14.2	[12.8, 15.6]
90% GVL/10% H_2O	Triflic	−	14.8	[13.9, 15.5]
90% GVL/10% H_2O	Triflic	KCl	14.4	[13.6, 15.2]

[a] Solution calorimetry conditions: fructose (2 mM); acid (5 mM); salt (5 mM); solvent (25 mL); temperature (298 K).

Supplementary Table 4. DFT-calculated pK_a values for HCl and H_2SO_4. All free energies are reported in kJ mol^{-1}.

Acid	$\Delta G_{(g)}$ (kJ mol^{-1})	$\Delta G_{(sol, H^+)}$ + $\Delta G_{(sol, B^-)}$ (kJ mol^{-1})	$\Delta G_{(sol, H\text{-}B)}$ (kJ mol^{-1})	$\Delta G_{(aq)}$ (kJ mol^{-1})	Calculated pK_a	Literature pK_a
HCl	1373 [26]	−1408 [28]	−8.4	−34.6	−6.1	−6.1 [31,32]
H_2SO_4	1265 [27]	−1394 [28]	−69.7	−67.3	−11.8	−10 [a],[33,34]

[a] There are two rather different pK_a values for H_2SO_4 in water reported in the literature: (1) the value of −10 reported in References [33,34], which is in good agreement with our calculated value of -11.8 and (2) the value of −2 reported in Reference [35], which is not referenced to any publication.

Supplementary Table 5. Fructose conversion reaction rate constant values and HMF yields for fructose dehydration to HMF in 90% GVL/10% H_2O (w/w) using heterogeneous acid/salt systems.[a]

Acid Catalyst	Solvent/Salt	Rate Constant (M^{-1} ks^{-1})[b]	HMF Yield (%)[c]
H_2SO_4	90% GVL/BTEAC	53	79
PSA/SiO_2	90% GVL	23	72
PSA/SiO_2	90% GVL/BTEAC	35	80
Amberlyst 70	90% GVL	31	70
Amberlyst 70	90% GVL/BTEAC	36	80

[a] Reaction conditions: fructose (50 mM); acid (5 mM); solid acid (25 μmol of acid sites); salt (5 mM); solvent (5 mL); stirring (700 rpm). [b] Rate constant values at 373 K; $r = k_{H+}$ [R] [H^+]. [c] HMF yield values at 393 K at approximately 90% conversion.

Supplementary Table 6. Example subset of reactions performed with experimental conditions for the Brønsted acid-catalyzed fructose dehydration to HMF in H_2O and polar aprotic solvent systems.

Solvent / Mass Fraction of Organic Solvent		Acid / Concentration ([M])		Salt / Concentration ([M])		Reaction Times (min)
Water		HCl	0.5	−	−	0, 15, 30, 45, 60
		H_2SO_4	0.5	−	−	0, 15, 30, 45, 60
		H_2SO_4	0.5	KCl	1	0, 15, 30, 45, 60
		H_2SO_4	0.5	KCl	2	0, 15, 30, 45, 60
		H_2SO_4	0.5	KCl	3	0, 15, 30, 45, 60
GVL	0.05	H_2SO_4	0.5	−	−	0, 15, 30, 45, 60
	0.25	H_2SO_4	0.5	−	−	0, 15, 30, 45, 60
	0.25	H_2SO_4	0.5	KCl	0.5	0, 15, 30, 45, 60
	0.50	H_2SO_4	0.1	−	−	0, 15, 30, 45, 60
	0.50	H_2SO_4	0.1	KCl	0.25	0, 15, 30, 45, 60
	0.75	H_2SO_4	0.025	−	−	0, 15, 30, 45, 60
	0.90	HCl	0.005	−	−	0, 15, 30, 45, 60
	0.90	Triflic	0.001	−	−	0, 30, 60, 90, 120
	0.90	Triflic	0.005	KBr	0.005	0, 10, 20, 30, 40
	0.90	Triflic	0.005	NaBr	0.005	0, 10, 20, 30, 40
	0.90	Triflic	0.005	LiBr	0.005	0, 10, 20, 30, 40
	0.90	Triflic	0.005	KCl	0.001	0, 10, 20, 30, 40
	0.90	Triflic	0.005	KCl	0.01	0, 10, 20, 30, 40
	0.90	Triflic	0.005	NaCl	0.005	0, 10, 20, 30, 40
	0.90	Triflic	0.005	LiCl	0.005	0, 10, 20, 30, 40
	0.90	−	−	KCl	0.005	0, 10, 20, 30, 40
THF	0.90	H_2SO_4	0.005	−	−	0, 15, 30, 45, 60
	0.90	Triflic	0.005	−	−	0, 15, 30, 45, 60
	0.90	HCl	0.005	−	−	0, 15, 30, 45, 60
Dioxane	0.90	H_2SO_4	0.005	−	−	0, 15, 30, 45, 60
	0.90	Triflic	0.005	−	−	0, 15, 30, 45, 60
	0.90	HCl	0.005	−	−	0, 15, 30, 45, 60

[a] Reaction conditions: fructose (50 mM); solvent (5 mL); temperature (373 K); stirring (700 rpm).

Supplementary Figures

a

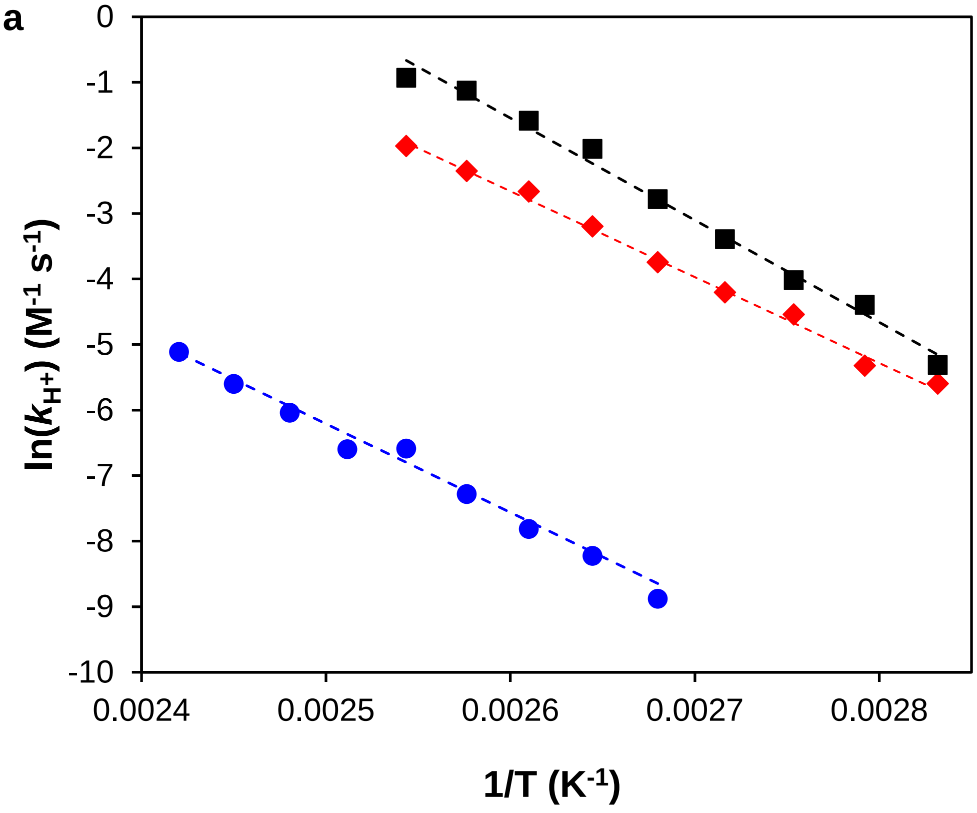

b

Solvent[a]	Catalyst	A[c,d] (M^{-1} s^{-1})	95% C.I. (M^{-1} s^{-1})	E_a[c,d] (kJ mol^{-1})	95% C.I. (kJ mol^{-1})
H_2O	HCl	1.04E12	[2.8E11, 3.9E12]	113	[108, 117]
90% GVL[b]	Triflic	4.38E13	[1.1E13, 1.8E14]	109	[105, 113]
90% GVL[b]	HCl	5.15E17	[5.5E16, 4.8E18]	135	[128, 142]

[a] Reaction conditions: fructose (50 mM); acid (0.025 – 0.5 M for water; 1 – 10 mM for 90% GVL/10% H_2O); solvent (5 mL); stirring (700 rpm). [b] 90% GVL with 10% H_2O (w/w). [c] $r = Ae^{-(Ea/RT)}$ [R] [H^+]. [d] Temperature ranges: 373 – 413 K for H_2O; 353 – 393 K for 90% GVL/10% H_2O.

Supplementary Figure 1. Fructose dehydration reaction energetics. **(a)** Arrhenius plot of the apparent reaction rate constant values at various temperatures for fructose dehydration to HMF in H_2O with HCl (blue circles); 90% GVL/10% H_2O with triflic acid (red diamonds); and 90% GVL/10% H_2O with HCl (black squares). **(b)** Table of apparent activation energy values derived from data in Supplementary Figure 1, a (reaction conditions listed in table footer).

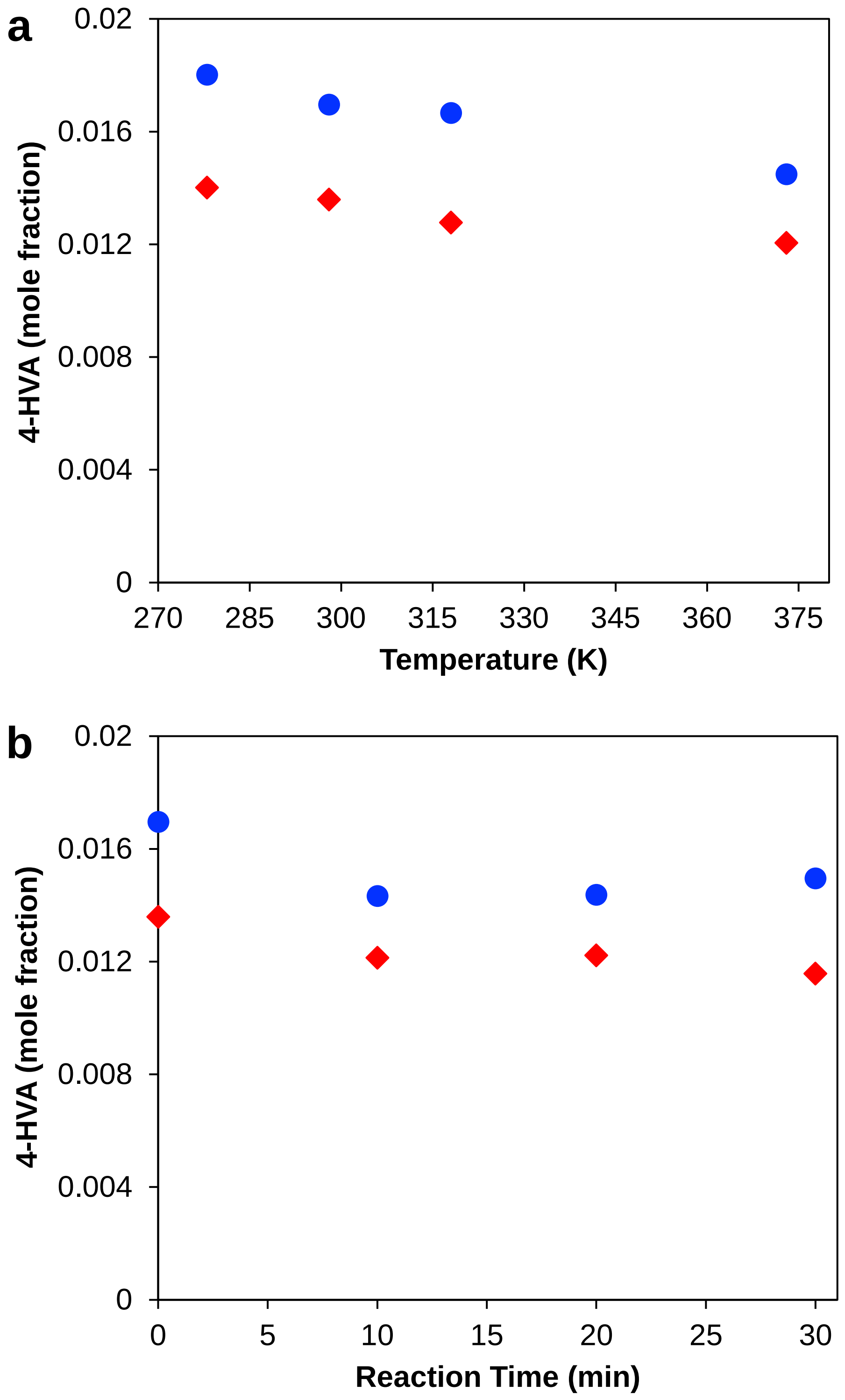

Supplementary Figure 2. NMR results with 4-HVA. Mole fraction of 4-HVA as function of **(a)** temperature and **(b)** reaction time during the fructose dehydration reaction in 90% GVL/10% H_2O (w/w) with H_2SO_4 (blue circles) and with H_2SO_4 and KCl (red diamonds). Reaction conditions: fructose (50 mM); acid (H_2SO_4; 5 mM); salt (KCl; 5 mM); 373 K for **(b)**; solvent (5 mL); stirring (700 rpm).

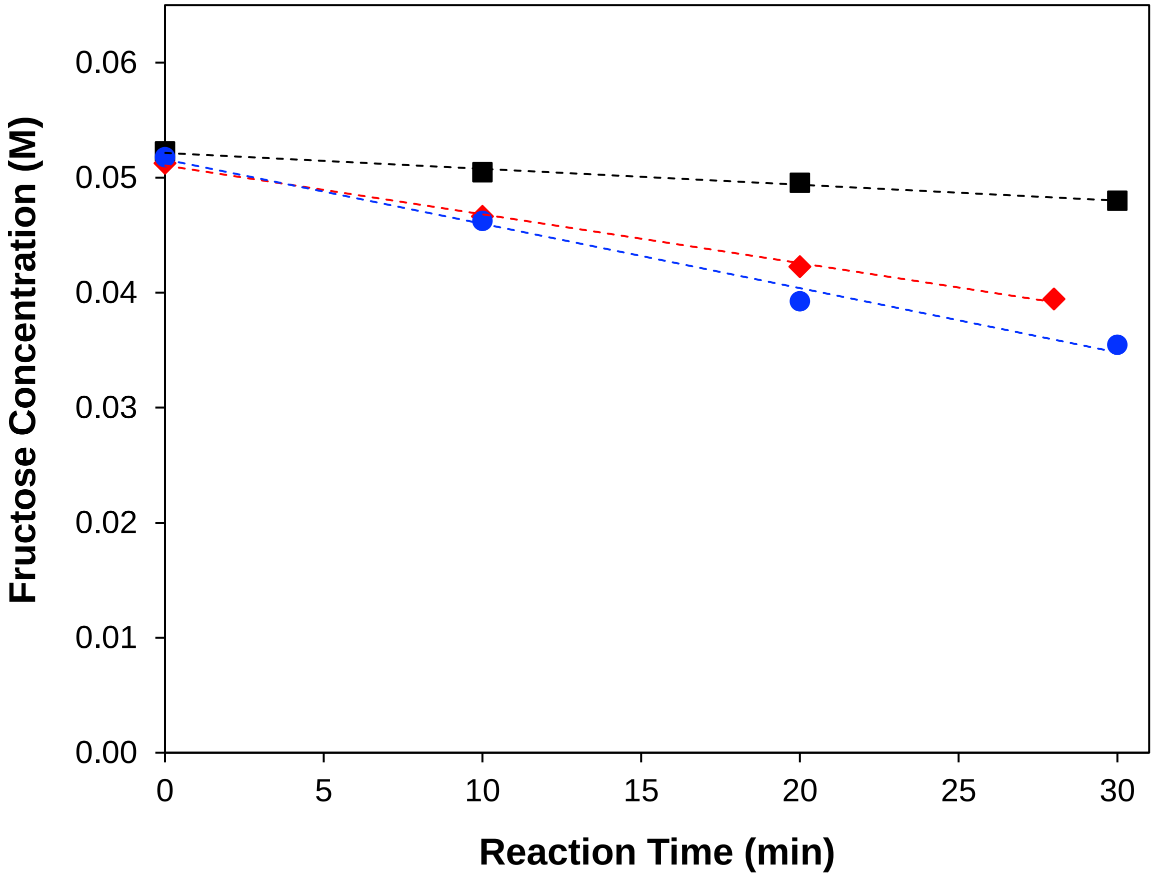

Supplementary Figure 3. Reaction kinetics data for fructose dehydration in 90% GVL/10% H_2O (w/w) with H_2SO_4 (black squares); H_2SO_4 and KCl (red diamonds); and H_2SO_4, KCl, and valeric acid (blue circles). Reaction conditions: fructose (50 mM); acid (H_2SO_4; 5 mM); salt (KCl; 5 mM); valeric acid (90 mM); 373 K; solvent (5 mL); stirring (700 rpm).

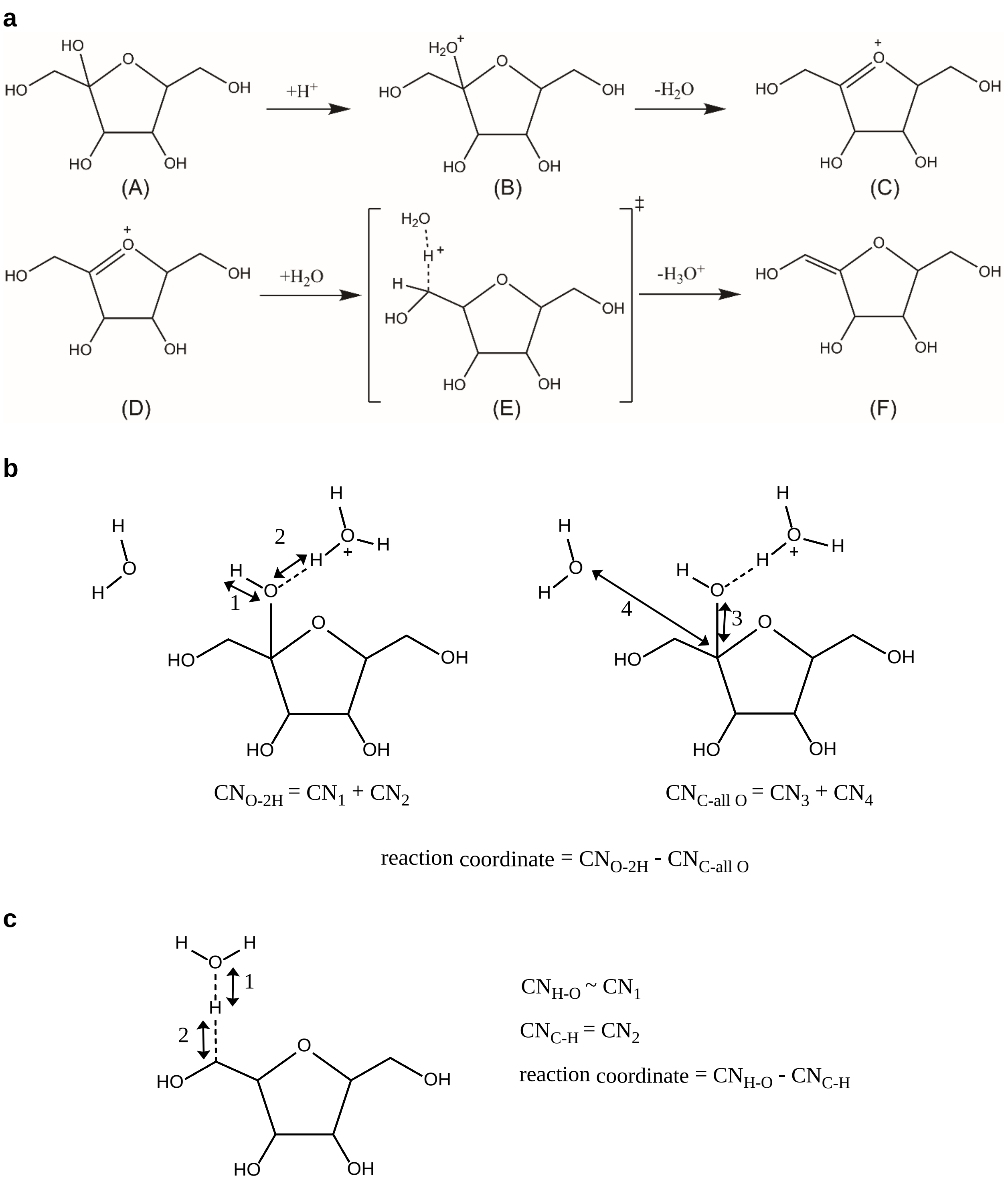

Supplementary Figure 4. Reaction schemes for the Brønsted-acid catalyzed conversion of fructose to HMF. **(a)** Proposed mechanism for fructose dehydration; **(b)** Reaction coordinate for the oxocarbenium ion formation step; and **(c)** Reaction coordinate for the proton abstraction step.

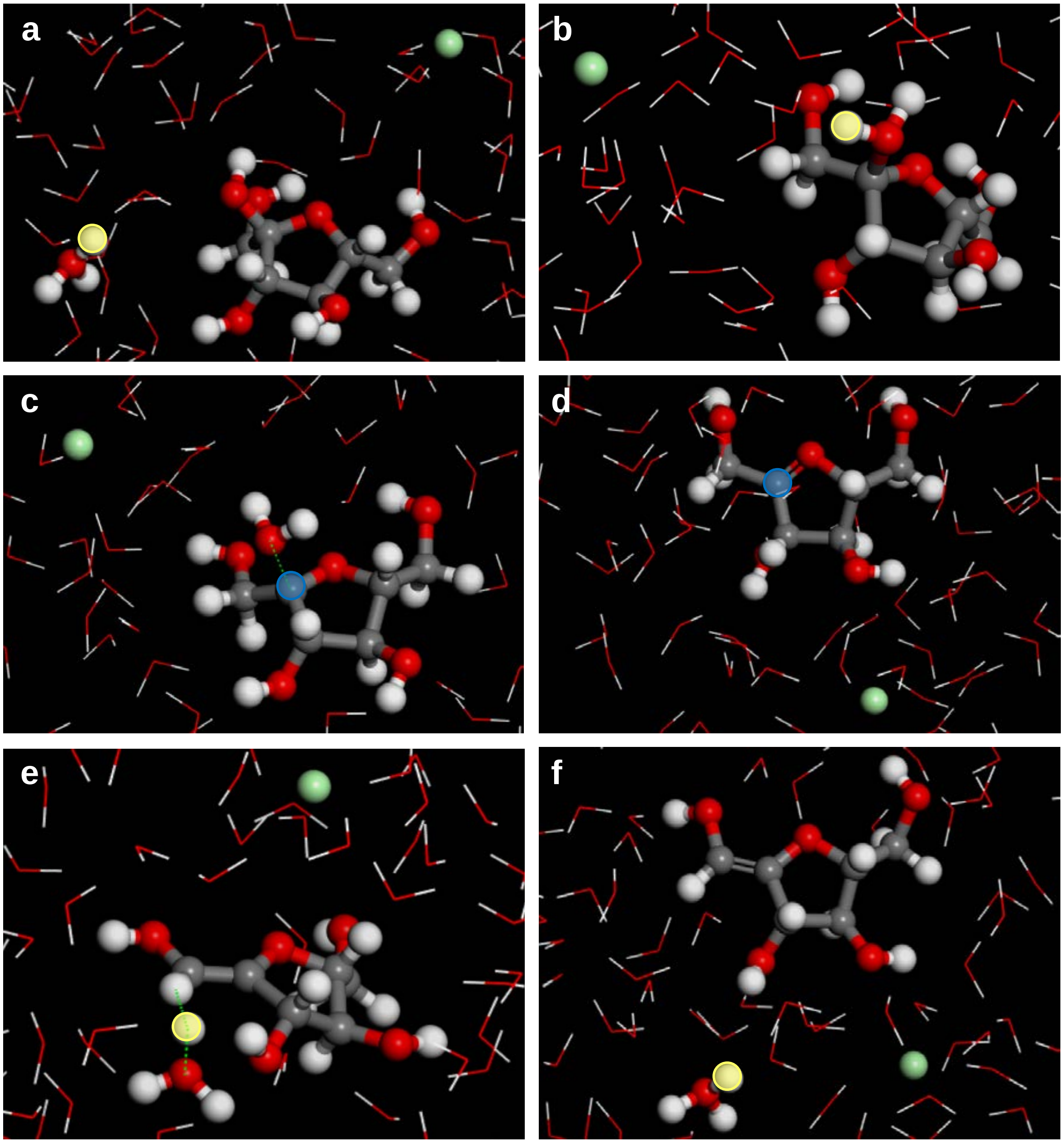

Supplementary Figure 5. The progression of fructose dehydration in water with HCl catalyst. **(a)** The initial state involves fully dissociated HCl as hydronium and chloride ions together with a single fructose molecule in water (proton is highlighted in yellow). **(b)** The protonated fructose structure without stabilization from chloride ion. **(c)** The transition state for the oxocarbenium ion formation step where the scission of C-O bond is shown via the dotted line. **(d)** The carbenium ion intermediate that results from fructose shows the formation of a double bond between ring oxygen and C2 carbon (center of the oxocarbenium ion shown in blue), suggesting a stabilization via the lone pair of electrons on the ring oxygen. **(e)** The transition state for the deprotonation of the oxocarbenium ion without stabilization from chloride ion. **(f)** The resulting olefin product. Figures in panels **(e)** and **(f)** were taken from the systems with bounded chloride anion in the initial state (Supplementary Figure 6, **g-i**). The red, grey, green, and white spheres refer to the oxygen, carbon, chlorine, and hydrogen atoms, respectively. The yellow and blue highlighted circles refer to the reactive proton and oxocarbenium ions centers, respectively.

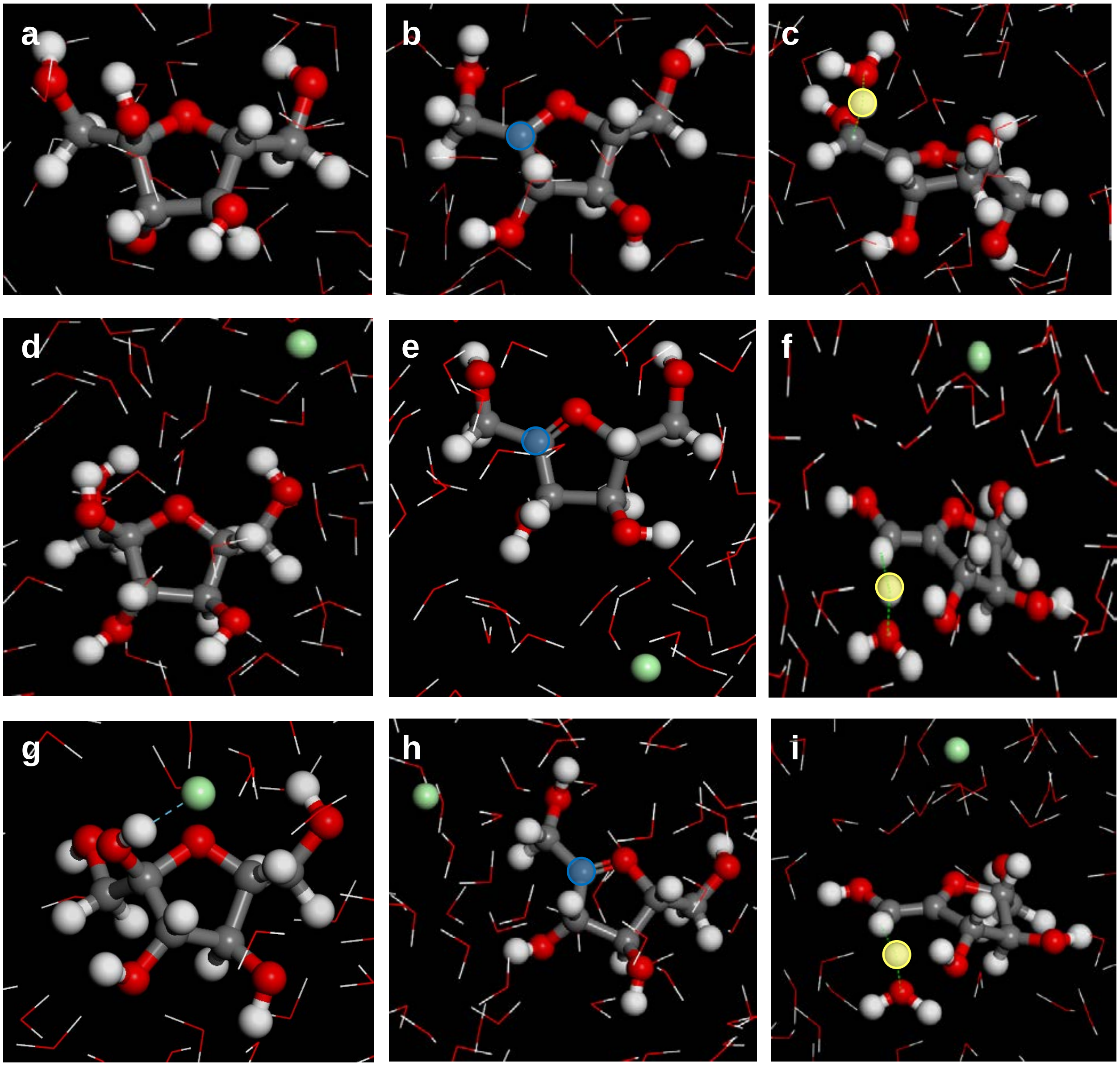

Supplementary Figure 6. Fructose dehydration reaction structures in water. Structures of fructose in the water system without an anion (in **a-c** of the first row), with a free chloride anion (in **d-f** of the second row), and with a bound chloride anion in the initial state (in **g-i** of the third row). The first, second, and third columns represent the initial, oxocarbenium ion, and transition state structures, respectively. The red, grey, green, and white spheres refer to the oxygen, carbon, chlorine, and hydrogen atoms, respectively. The yellow and blue highlighted circles refer to the reactive proton and oxocarbenium ions centers, respectively.

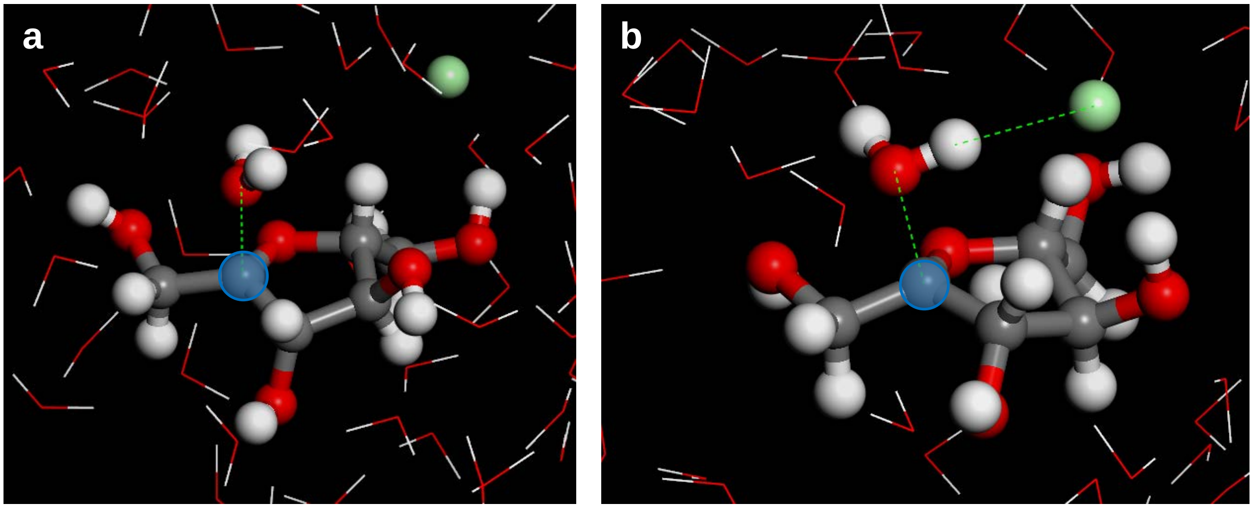

Supplementary Figure 7. Structures of the transition state for the oxocarbenium formation step for fructose dehydration in H_2O. **(a)** The chloride anion was solvated in bulk water in the initial state. The transition state of the oxocarbenium formation step does not involve the anion. **(b)** The chloride anion was deliberately bound to the fructose hydroxyl group in the initial state. The transition state of the oxocarbenium formation step is stabilized by chloride anion. The red, grey, green, and white spheres refer to the oxygen, carbon, chlorine, and hydrogen atoms, respectively. The blue highlighted circle refers to the oxocarbenium ions center.

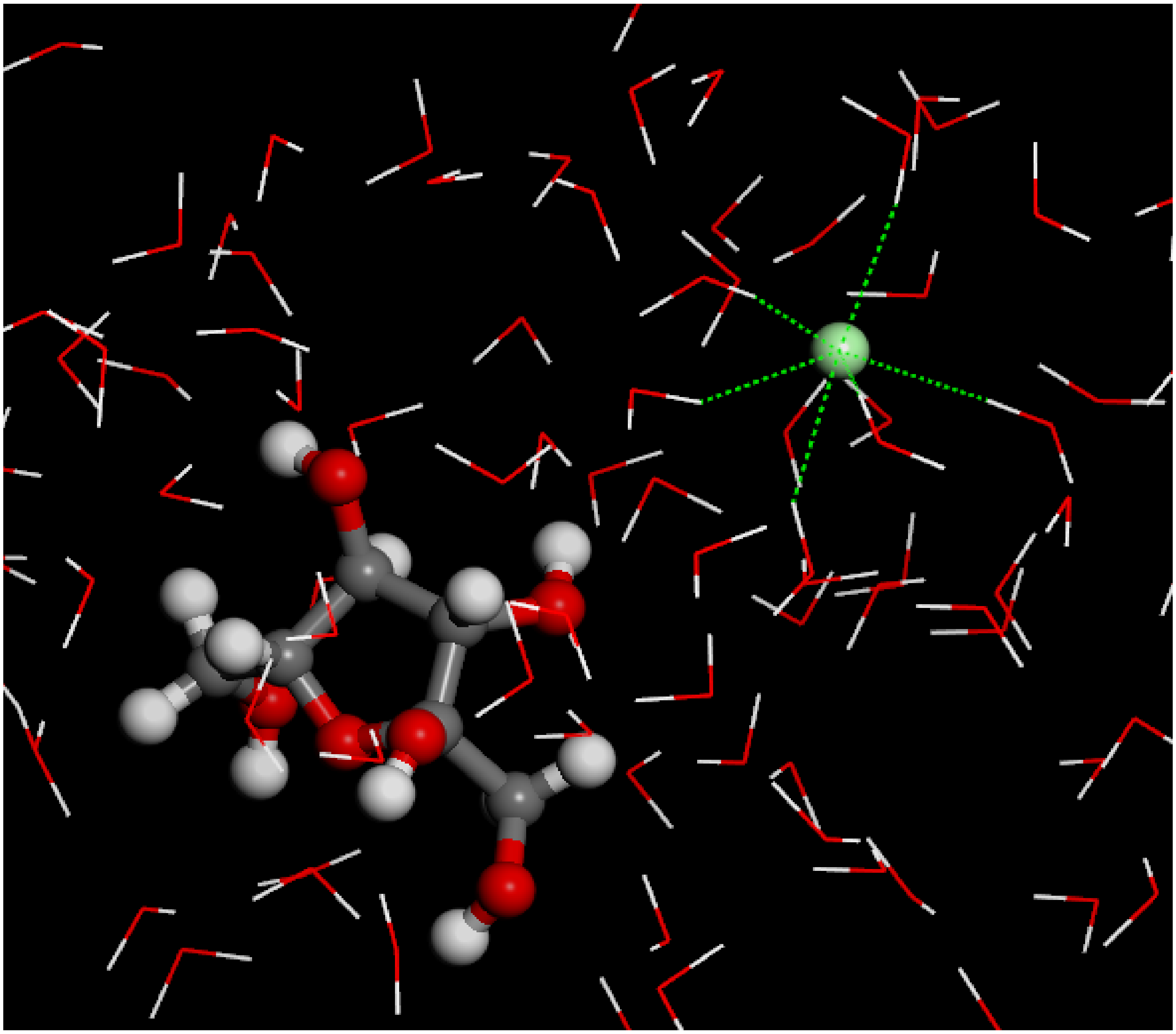

Supplementary Figure 8. The chloride anion in the water system shows extensive hydrogen bonding in the water solvation shell. The interaction between the chloride anion and the reactive site on fructose is inhibited as the anion is locked up in the solution phase. The red, grey, green, and white spheres refer to the oxygen, carbon, chlorine, and hydrogen atoms, respectively.

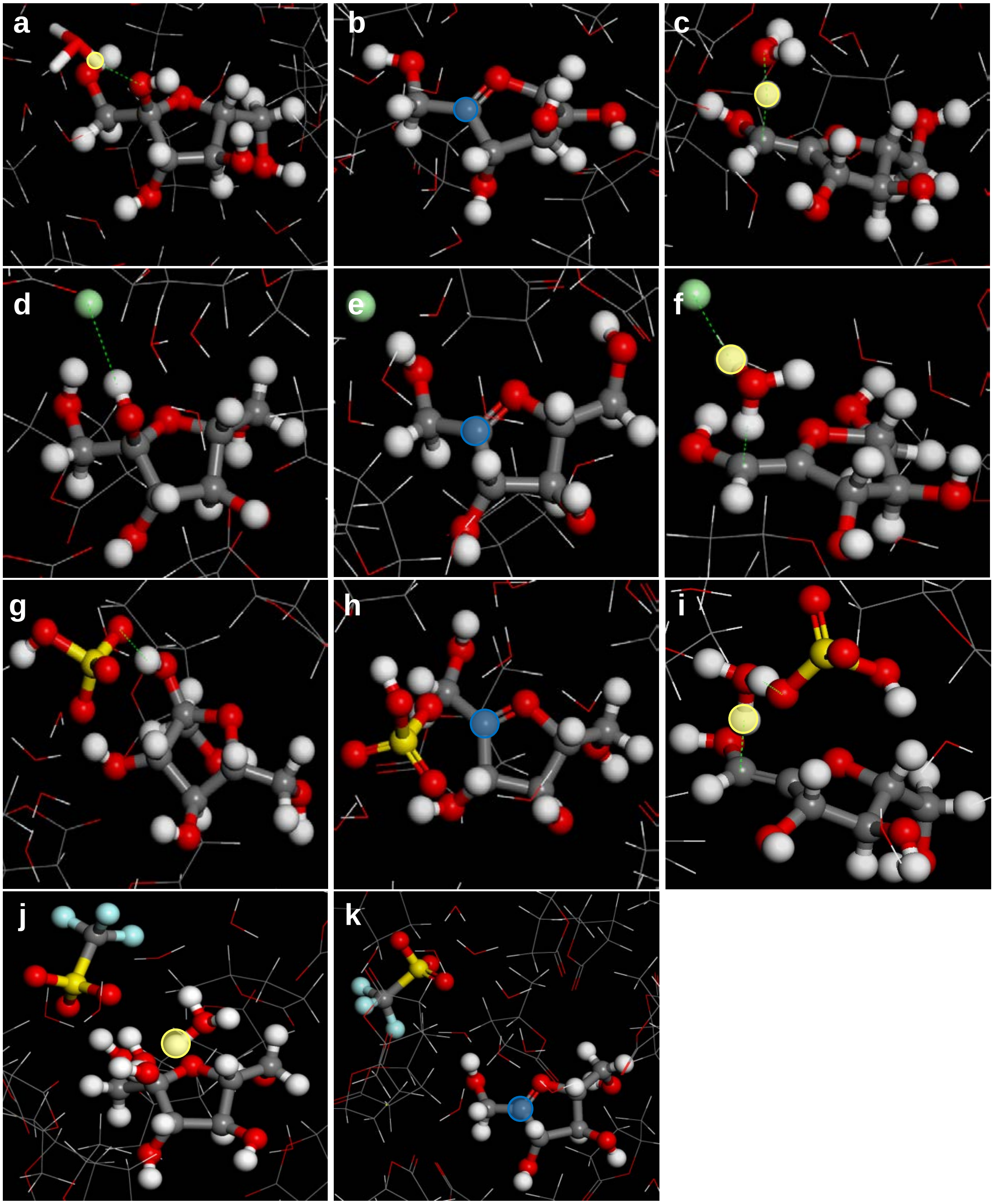

Supplementary Figure 9. Fructose dehydration reaction structures in GVL. Structures of fructose in the 90% GVL/10% H_2O (w/w) systems without an anion (**a-c**, first row), with a bound chloride anion in the initial state (**d-f**, second row), with a bound bisulfate anion in the initial state (**g-i**, third row), and with a bound triflate anion in the initial state (**j** and **k**, fourth row). The first, second, and third columns represent the initial, oxocarbenium ion, and transition state structures, respectively. The red, grey, green, white, yellow, and aqua spheres refer to the oxygen, carbon, chlorine, hydrogen, sulfur, and fluorine atoms, respectively. The yellow and blue highlighted circles refer to the reactive proton and carbenium ions centers, respectively.

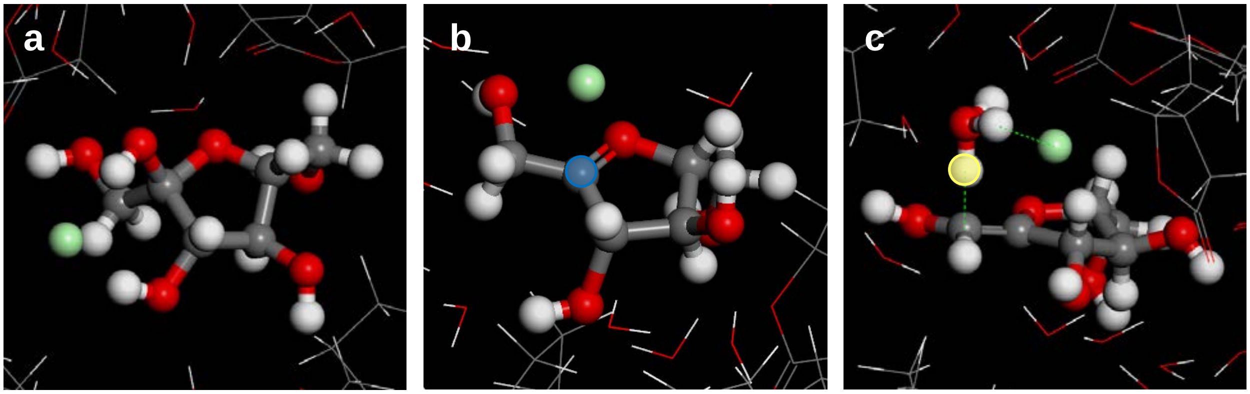

Supplementary Figure 10. Structures of fructose in 75% GVL/25% H_2O (w/w) with a bound chloride anion in the initial state. The first **(a)**, second **(b)**, and third **(c)** panel correspond to initial, oxocarbenium ion, and transition state structures, respectively. The red, grey, green, and white spheres refer to the oxygen, carbon, chlorine, and hydrogen atoms, respectively. The yellow and blue highlighted circles refer to the reactive proton and oxocarbenium ions centers, respectively.

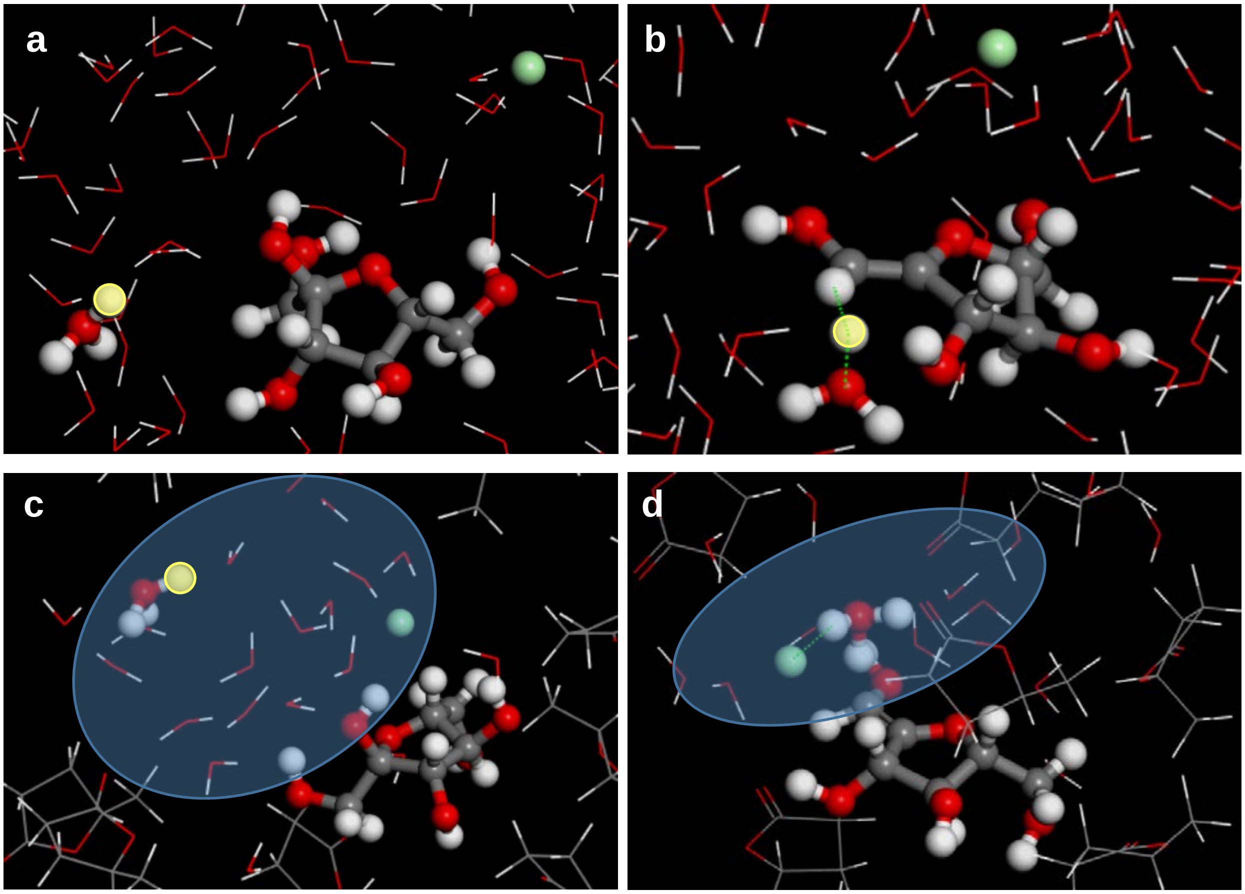

Supplementary Figure 11. Fructose dehydration initial and transition states in water and GVL. **(a)** The initial state and **(b)** the transition state of fructose dehydration in water with hydrochloric acid catalyst. The acid is fully dissociated into hydronium and chloride ions, where both the ions are located in the bulk solvent far from fructose. **(c)** The solvation structures in 75% GVL/25% H_2O (w/w) shows a localization of water molecules in the initial state. The dissociation of acid takes place inside the hydrophilic domain, allowing for stabilization of the transition state by the chloride anion **(d)**. The red, grey, green, and white spheres refer to the oxygen, carbon, chlorine, and hydrogen atoms, respectively. The yellow highlighted circle refers to the reactive proton. The blue circled regions highlight the localized hydrophilic domains.

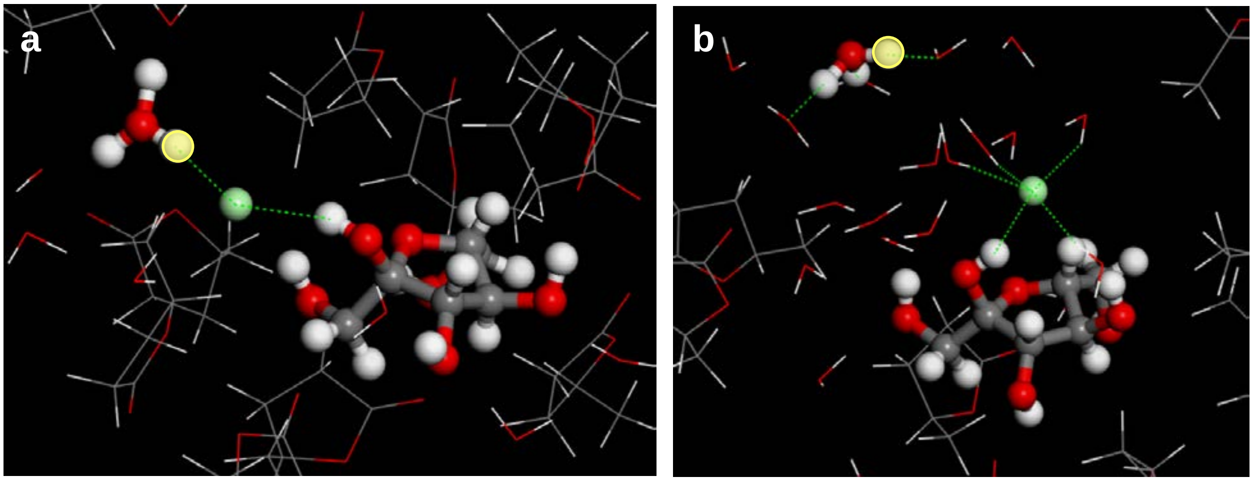

Supplementary Figure 12. A comparison of 0.5 M HCl acid dissociation various GVL solvents. **(a)** 90% GVL/10% H_2O (w/w) and **(b)** 75% GVL/25% H_2O (w/w). An ion pairing is observed in the 90% GVL/10% H_2O system, while the acid is fully dissociated in the 75% GVL/25% H_2O system. Incomplete acid dissociation was not observed in the experimental results since the acid concentration used was much lower (~5 mM acid). The red, grey, green, and white spheres refer to the oxygen, carbon, chlorine, and hydrogen atoms, respectively. The yellow highlighted circle refers to the proton.

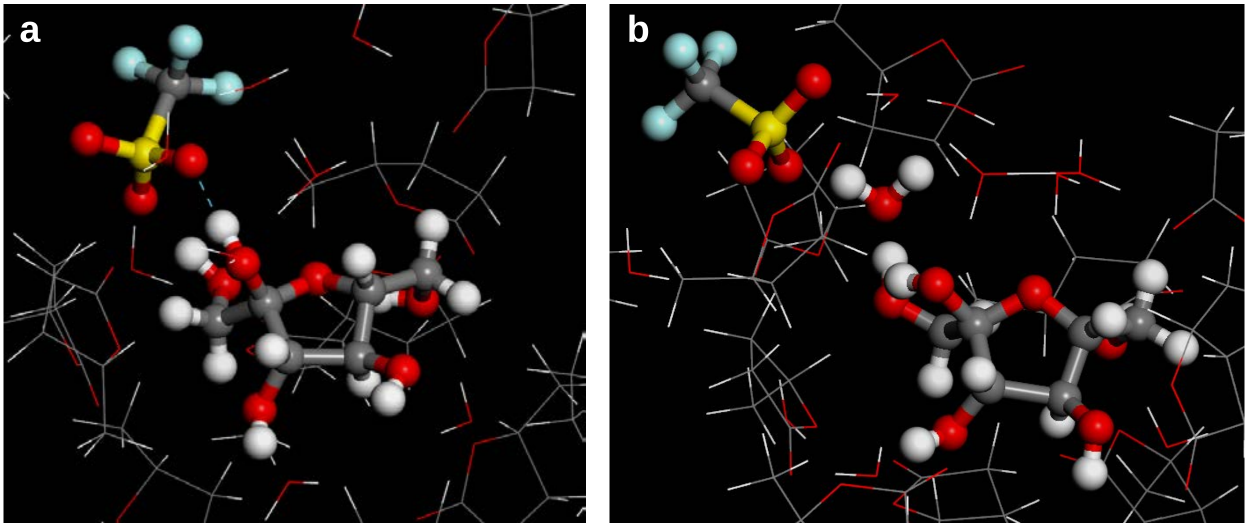

Supplementary Figure 13. Fructose dehydration reaction structures with triflate anion. **(a)** Structures of fructose in 90% GVL/10% H_2O (w/w) with a bounded triflate anion in the initial state. **(b)** The triflate ion in the previous structure migrated away from the hydrophilic domain near fructose molecule partitions with the SO_3 group of triflate in the hydrophilic domain whereas the CF_3 group is in the hydrophobic domain. The interaction between the triflate anion and reactive site near fructose is inhibited. The red, grey, green, white, yellow, and aqua spheres refer to the oxygen, carbon, chlorine, hydrogen, sulfur, and fluorine atoms, respectively.

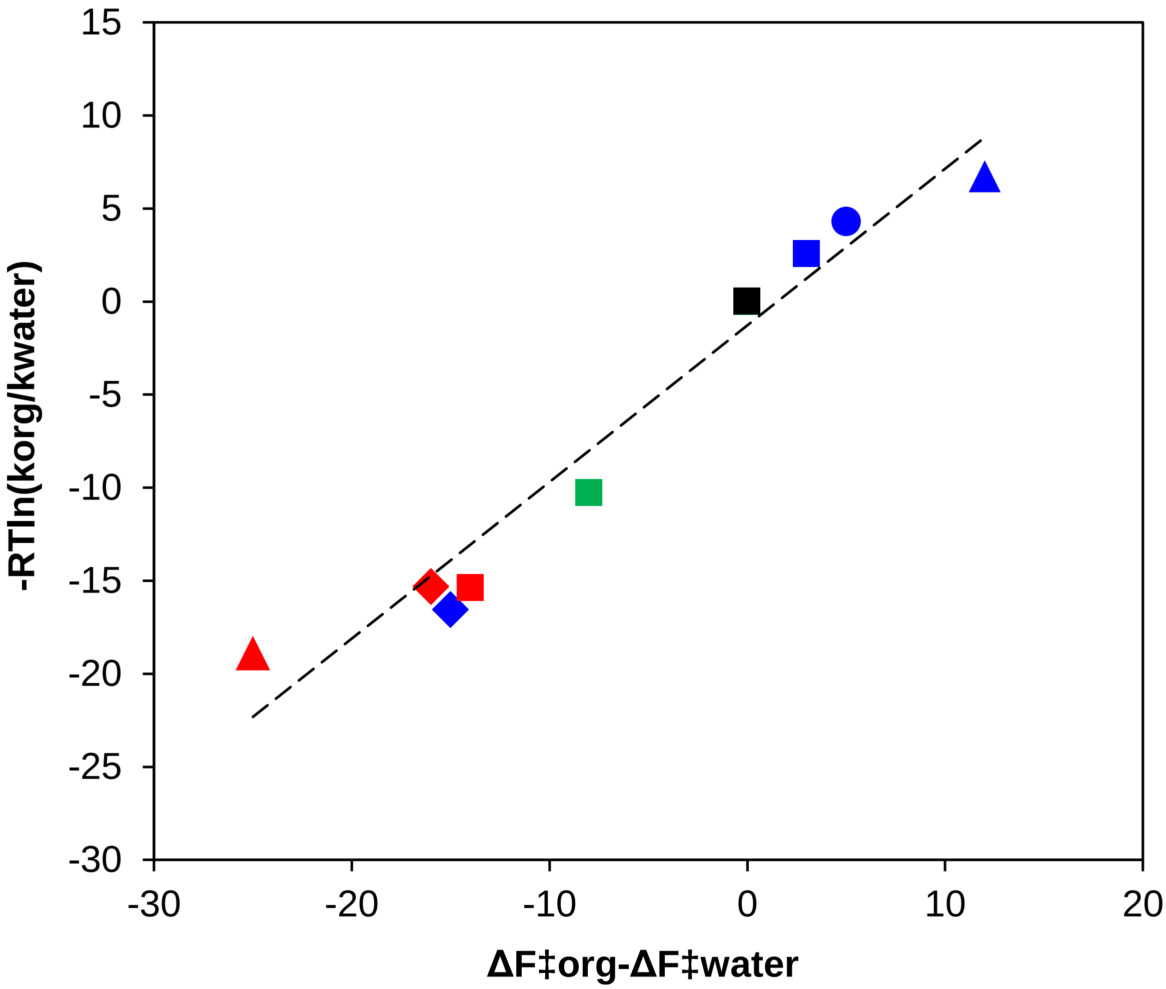

Supplementary Figure 14. Linear relationship between the experimentally measured and AIMD simulated activation free energies for the acid-catalyzed dehydration of different alcohols in different solvents and acids. Each data point corresponds to the following cases:

tert-butanol in H_2O with H^+ (black square)[24]

tert-butanol in 70% GVL/10% H_2O with H^+ (blue square)[24]

tert-butanol in 100% GVL with H^+ (blue diamond)[24]

tert-butanol in 90% DMSO/10% H_2O with H^+ (blue triangle) [24]

tert-butanol in 100% DMSO with H^+ (blue circle)[24]

1,2-propanediol in H_2O with H^+ (black square)[24]

1,2-propanediol in 90% GVL/10% H_2O with H^+ (green square)[24]

fructose in H_2O with H^+ (black square)

fructose in 90% GVL/10% H_2O with H_2SO_4 (red square)

fructose in 90% GVL/10% H_2O with triflic acid (red diamond)

fructose in 90% GVL/10% H_2O with HCl (red triangle)

The black dotted line is the best fit line equation $y = 0.8416x – 1.2747$ with the R^2 value of 0.9538, which represents a linear fit to the data. The data for H^+ acid did not include an anion in computational simulations, while triflic acid was used in the experiments.

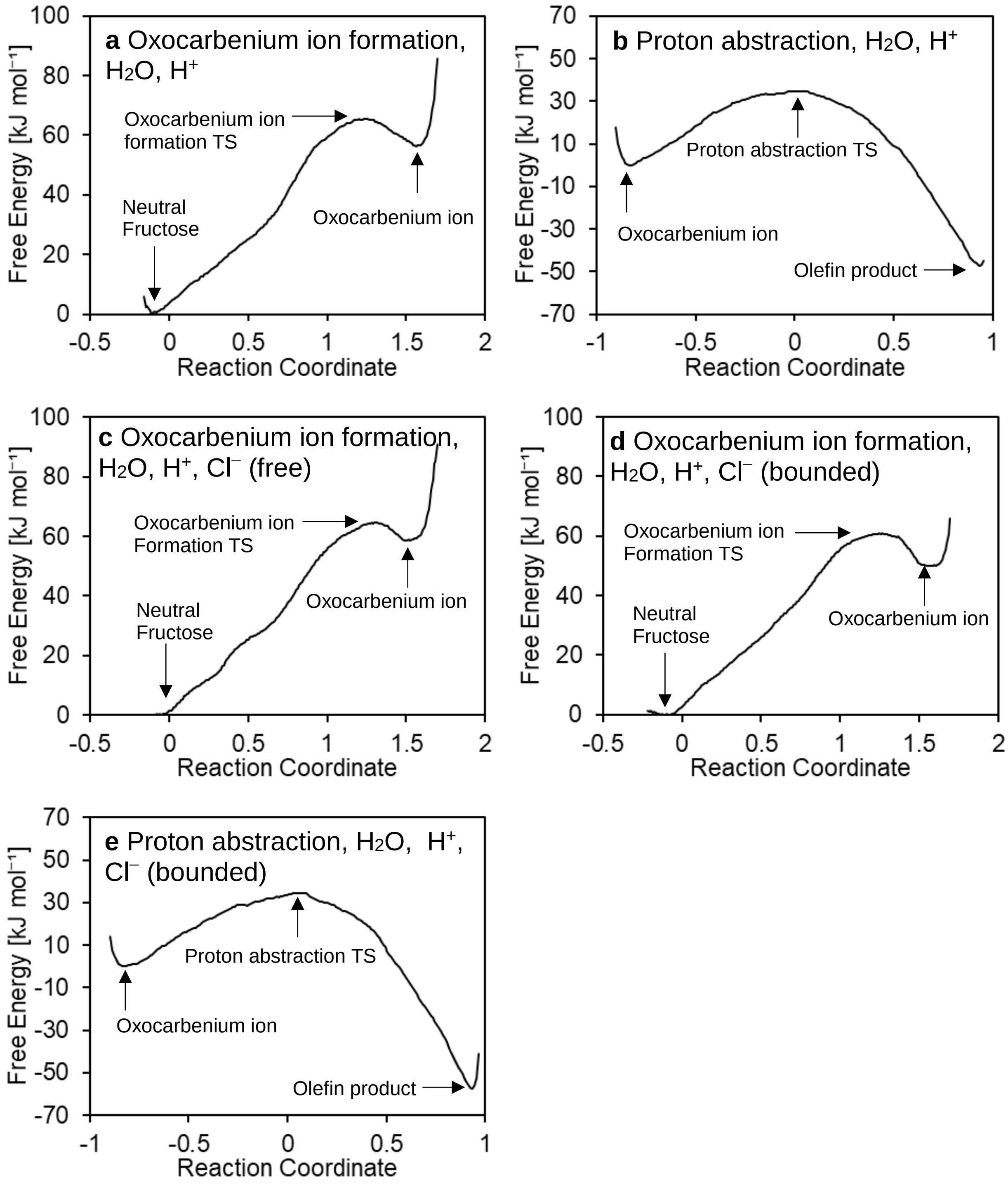

Supplementary Figure 15. Free energy profiles for fructose dehydration in water systems. **(a)** oxocarbenium ion formation without anion; **(b)** proton abstraction without anion; **(c)** carbenium ion formation with free chloride anion; **(d)** oxocarbenium ion formation with bounded chloride anion in the initial state; **(e)** proton abstraction proceeded from structure in **(d)**. These figures **(a-e)** correspond to the structures shown in Supplementary Figure 6.

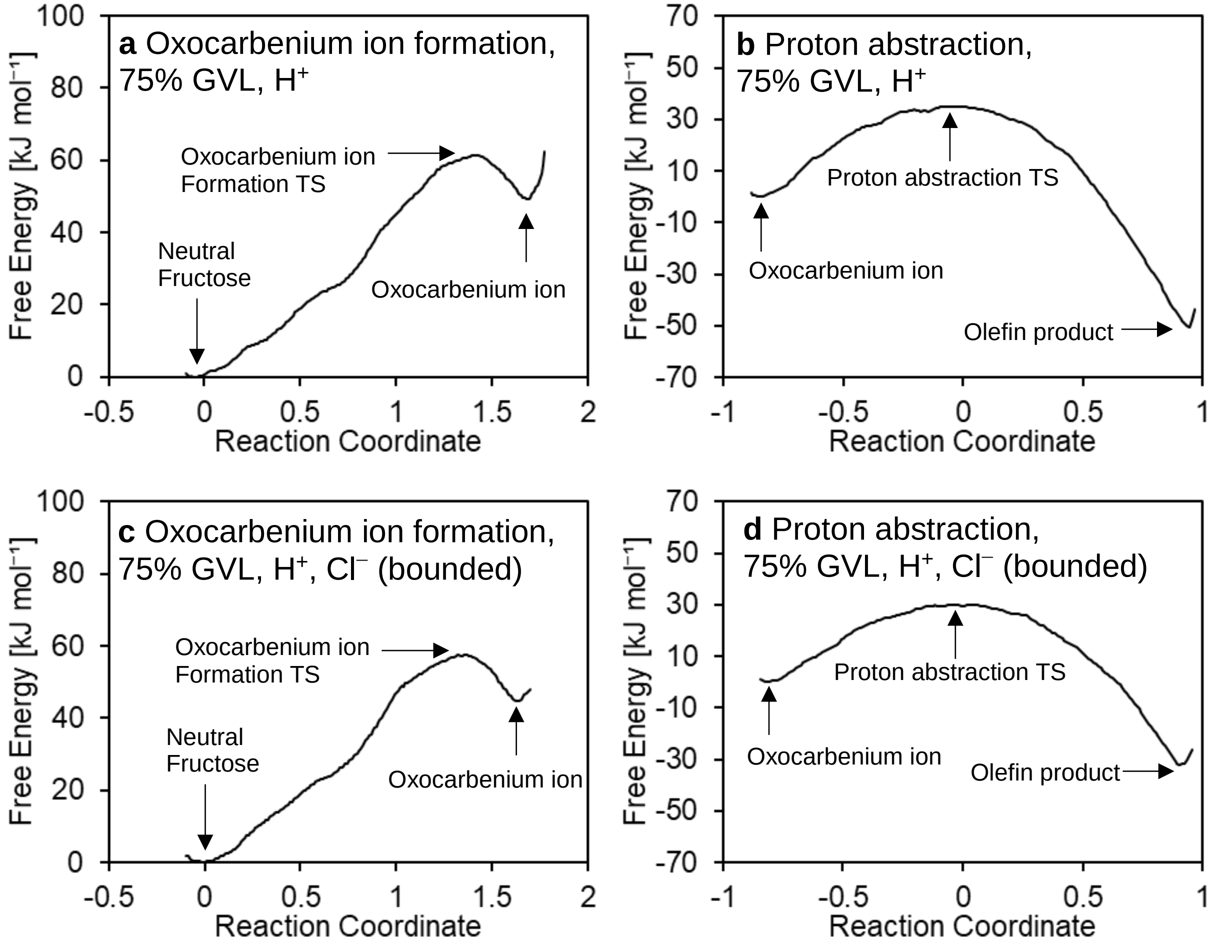

Supplementary Figure 16. Free energy profiles for fructose dehydration in 75% GVL/25% H_2O (w/w) systems. **(a)** oxocarbenium ion formation without anion; **(b)** proton abstraction without anion; **(c)** oxocarbenium ion formation with bounded chloride anion in the initial state; **(d)** proton abstraction proceeded from structure in **(c)**.

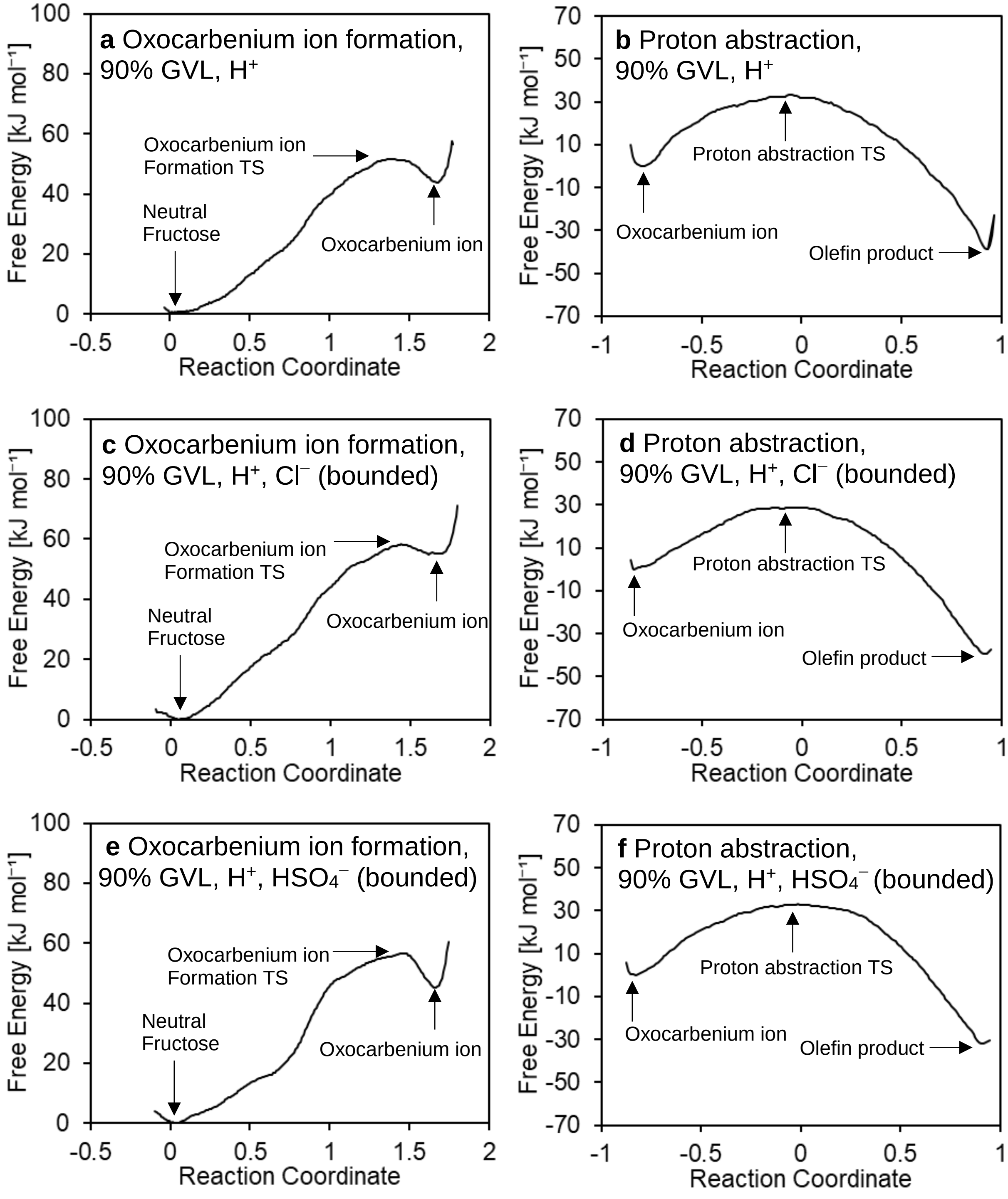

Supplementary Figure 17. Free energy profiles of fructose dehydration in 90% GVL/10% H_2O (w/w) systems. **(a)** oxocarbenium ion formation without anion; **(b)** proton abstraction without anion; **(c)** oxocarbenium ion formation with bounded chloride anion in the initial state; **(d)** proton abstraction proceeded from structure in **(c)**; **(e)** oxocarbenium ion formation with bounded bisulfate anion in the initial state; **(f)** proton abstraction proceeded from structure in **(e)**. These figures **(a-f)** correspond to the structures shown in Supplementary Figure 9.

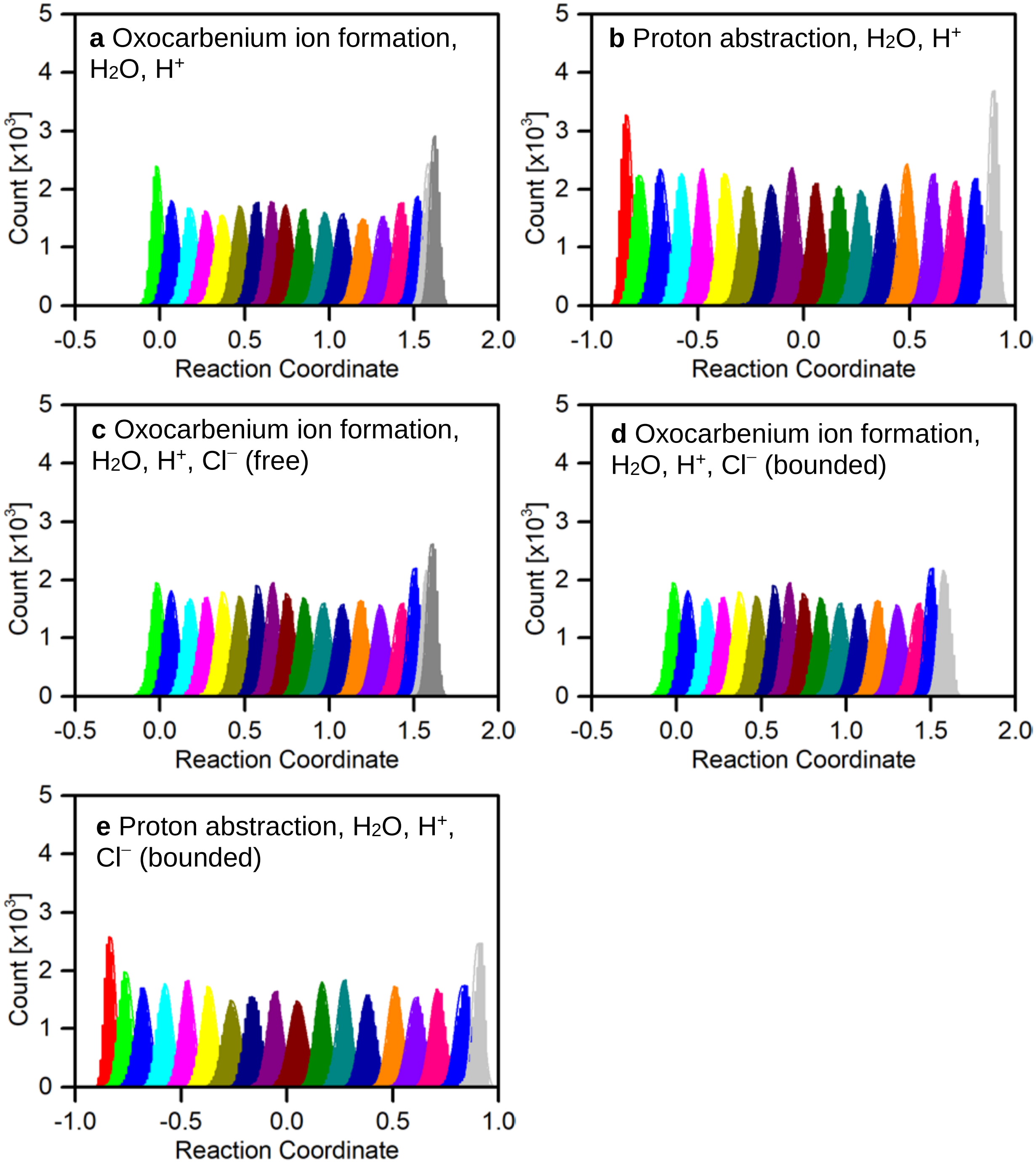

Supplementary Figure 18. Statistical histograms of free energy sampling for fructose dehydration in water systems. **(a)** oxocarbenium ion formation without anion; **(b)** proton abstraction without anion; **(c)** oxocarbenium ion formation with free chloride anion; **(d)** oxocarbenium ion formation with bounded chloride anion in the initial state; **(e)** proton abstraction proceeded from structure in **(d)**. These histograms **(a-e)** correspond to the structures in Supplementary Figure 6 and free energy profiles in Supplementary Figure 15.

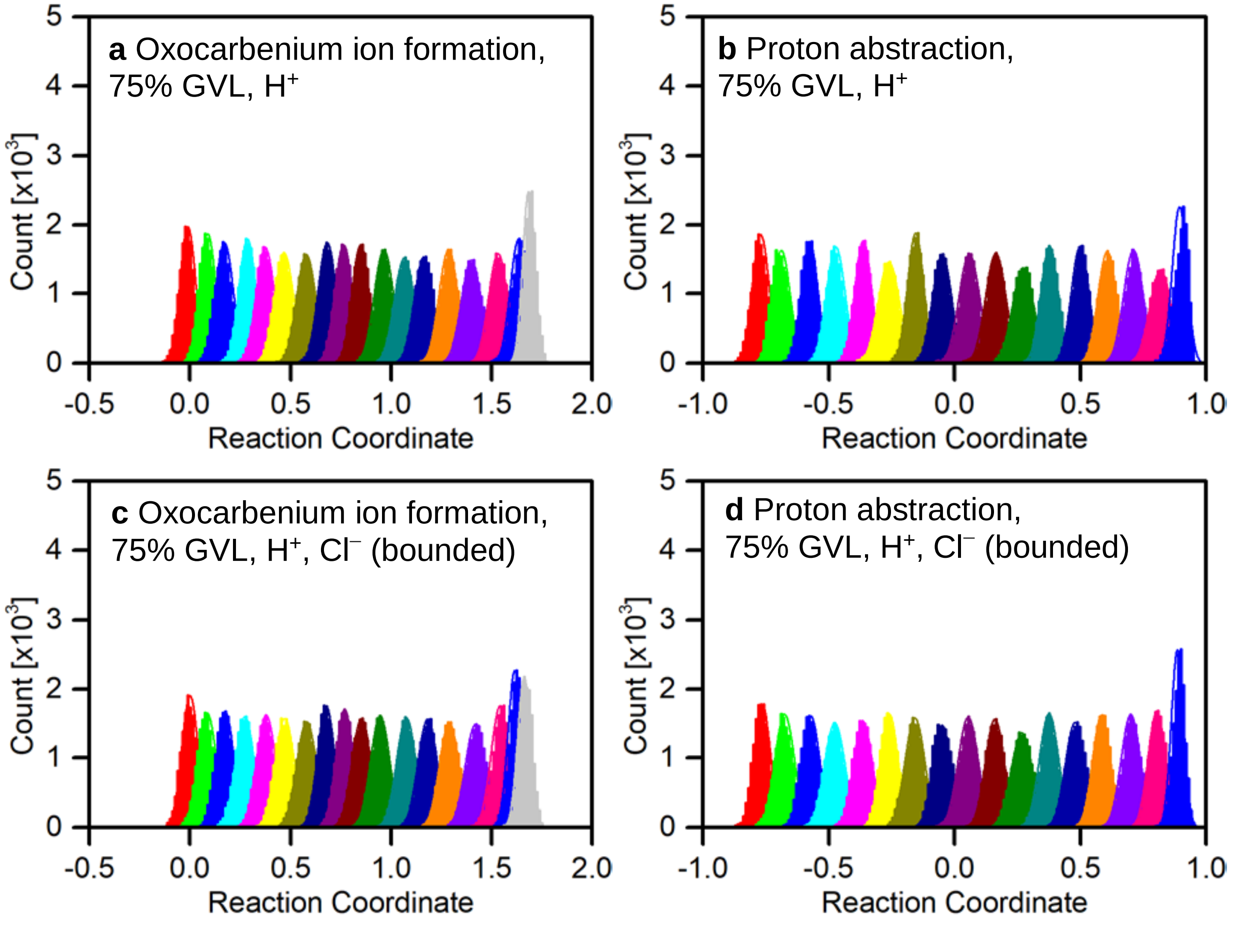

Supplementary Figure 19. Statistical histograms of free energy sampling for fructose dehydration in 75% GVL/25% H_2O (w/w) systems. **(a)** oxocarbenium ion formation without anion; **(b)** proton abstraction without anion; **(c)** oxocarbenium ion formation with bounded chloride anion in the initial state, **(d)** proton abstraction proceeded from structure in **(c)**. These histograms **(a-d)** correspond to the free energy profiles in Supplementary Figure 16.

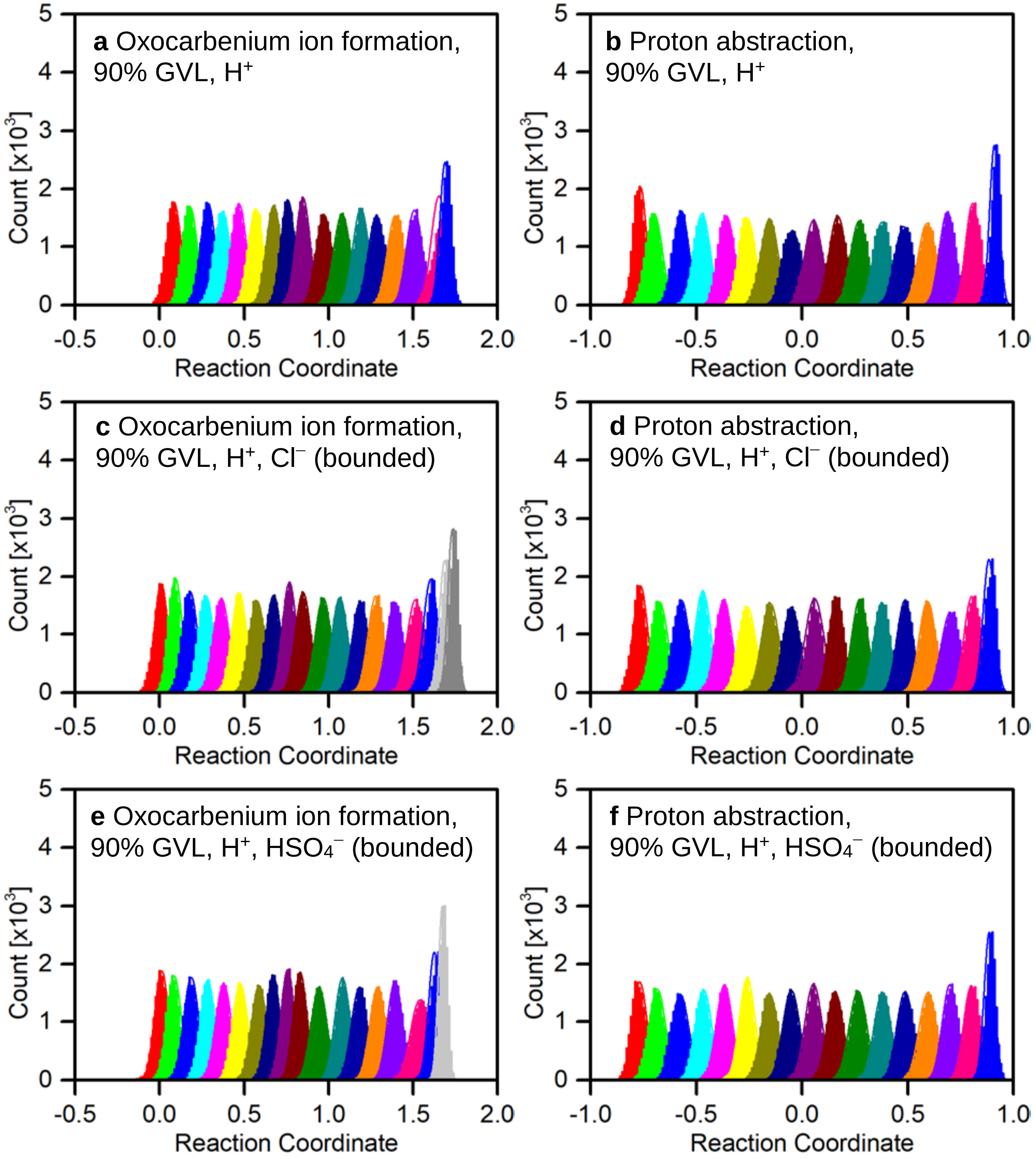

Supplementary Figure 20. Statistical histograms of free energy sampling for fructose dehydration in 90% GVL/10% H_2O (w/w) systems. **(a)** oxocarbenium ion formation without anion; **(b)** proton abstraction without anion; **(c)** oxocarbenium ion formation with bounded chloride anion in the initial state; **(d)** proton abstraction proceeded from structure in **(c)**; **(e)** oxocarbenium ion formation with bounded bisulfate anion in the initial state; **(f)** proton abstraction proceeded from structure in **(e)**; These histograms **(a-f)** correspond to the structures in Supplementary Figure 9 and free energy profiles in Supplementary Figure 17.

Supplementary References

[1] Swift, T. D. *et al.* Kinetics of Homogeneous Brønsted Acid Catalyzed Fructose Dehydration and 5-Hydroxymethyl Furfural Rehydration: A Combined Experimental and Computational Study. *ACS Catal.* **4**, 259-267 (2014).

[2] Martínez, L., Andrade, R., Birgin, E. G. & Martínez, J. M. PACKMOL: A package for building initial configurations for molecular dynamics simulations. *J. Comput. Chem.* **30**, 2157-2164 (2009).

[3] Abraham, M. J. *et al.* GROMACS: High performance molecular simulations through multi-level parallelism from laptops to supercomputers. *SoftwareX* **1-2**, 19-25 (2015).

[4] Hutter, J., Iannuzzi, M., Schiffmann, F. & VandeVondele, J. cp2k: atomistic simulations of condensed matter systems. *Wiley Interdiscip. Rev.: Comput. Mol. Sci.* **4**, 15-25 (2013).

[5] Goga, N., Rzepiela, A. J., de Vries, A. H., Marrink, S. J. & Berendsen, H. J. C. Efficient Algorithms for Langevin and DPD Dynamics. *J. Chem. Theory Comput.* **8**, 3637-3649 (2012).

[6] Parrinello, M. & Rahman, A. Polymorphic transitions in single crystals: A new molecular dynamics method. *J. Appl. Phys.* **52**, 7182-7190 (1981).

[7] Nosé, S. & Klein, M. L. Constant pressure molecular dynamics for molecular systems. *Mol. Phys.* **50**, 1055-1076 (1983).

[8] Jorgensen, W. L., Maxwell, D. S. & Tirado-Rives, J. Development and Testing of the OPLS All-Atom Force Field on Conformational Energetics and Properties of Organic Liquids. *J. Am. Chem. Soc.* **118**, 11225-11236 (1996).

[9] Kony, D., Damm, W., Stoll, S. & Van Gunsteren, W. F. An improved OPLS–AA force field for carbohydrates. *J. Comput. Chem.* **23**, 1416-1429 (2002).

[10] Bonthuis, D. J., Mamatkulov, S. I. & Netz, R. R. Optimization of classical nonpolarizable force fields for OH− and H3O+. *J. Chem. Phys.* **144**, 104503 (2016).

[11] Koneshan, S., Rasaiah, J. C., Lynden-Bell, R. M. & Lee, S. H. Solvent Structure, Dynamics, and Ion Mobility in Aqueous Solutions at 25 °C. *J. Phys. Chem. B* **102**, 4193-4204 (1998).

[12] Canongia Lopes, J. N., Pádua, A. A. H. & Shimizu, K. Molecular Force Field for Ionic Liquids IV: Trialkylimidazolium and Alkoxycarbonyl-Imidazolium Cations; Alkylsulfonate and Alkylsulfate Anions. *J. Phys. Chem. B* **112**, 5039-5046 (2008).

[13] Sunda Anurag, P. & Venkatnathan, A. Molecular dynamics simulations of triflic acid and triflate ion/water mixtures: A proton conducting electrolytic component in fuel cells. *J. Comput. Chem.* **32**, 3319-3328 (2011).

[14] Berendsen, H. J. C., Grigera, J. R. & Straatsma, T. P. The missing term in effective pair potentials. *J. Phys. Chem.* **91**, 6269-6271 (1987).

[15] Miyamoto, S. & Kollman Peter, A. Settle: An analytical version of the SHAKE and RATTLE algorithm for rigid water models. *J. Comput. Chem.* **13**, 952-962 (1992).

[16] Perdew, J. P., Burke, K. & Ernzerhof, M. Generalized Gradient Approximation Made Simple. *Phys. Rev. Lett.* **77**, 3865-3868 (1996).

[17] Goedecker, S., Teter, M. & Hutter, J. Separable dual-space Gaussian pseudopotentials. *Phys. Rev. B* **54**, 1703-1710 (1996).

[18] VandeVondele, J. & Hutter, J. Gaussian basis sets for accurate calculations on molecular systems in gas and condensed phases. *J. Chem. Phys.* **127**, 114105 (2007).

[19] Grimme, S., Ehrlich, S. & Goerigk, L. Effect of the damping function in dispersion corrected density functional theory. *J. Comput. Chem.* **32**, 1456-1465 (2011).

[20] Bussi, G., Donadio, D. & Parrinello, M. Canonical sampling through velocity rescaling. *J. Chem. Phys.* **126**, 014101 (2007).

[21] Grossfield, A. WHAM: the weighted histogram analysis method, version 2.0.9 (Rochester University, Rochester, NY); http://membrane.urmc.rochester.edu/content/wham (2013).

[22] Reichardt, C. & Welton, T. *Solvents and Solvent Effects in Organic Chemistry*; Wiley-VCH Verlag GmbH & Co. KGaA: Weinheim, Germany, pp.165-173 (2010).

[23] Madon, R. J. & Iglesia, E. Catalytic reaction rates in thermodynamically non-ideal systems. *J. Mol. Catal. A: Chem.* **163**, 189-204 (2000).

[24] Mellmer, M. A. *et al.* Solvent-enabled control of reactivity for liquid-phase reactions of biomass-derived compounds. *Nat. Catal.* **1**, 199-207 (2018).

[25] Akien, G. R., Qi, L. & Horváth, I. T. Molecular mapping of the acid catalysed dehydration of fructose. *Chem. Commun.* **48**, 5850-5852 (2012).

[26] Martin, J. D. D. & Hepburn, J. W. Determination of bond dissociation energies by threshold ion-pair production spectroscopy: An improved D0(HCl). *J. Chem. Phys.* **109**, 8139-8142 (1998).

[27] Wang, X.-B., Nicholas, J. B. & Wang, L.-S. Photoelectron Spectroscopy and Theoretical Calculations of SO4- and HSO4-: Confirmation of High Electron Affinities of SO4 and HSO4. *J. Phys. Chem. A* **104**, 504-508 (2000).

[28] Marcus, Y. *Ions in Solution and their Solvation*; John Wiley & Sons, Inc: Hoboken, NJ, pp.113-115 (2015).

[29] Walker, M., Harvey, A. J. A., Sen, A. & Dessent, C. E. H. Performance of M06, M06-2X, and M06-HF Density Functionals for Conformationally Flexible Anionic Clusters: M06 Functionals Perform Better than B3LYP for a Model System with Dispersion and Ionic Hydrogen-Bonding Interactions. *J. Phys. Chem. A* **117**, 12590-12600 (2013).

[30] Marenich, A. V., Cramer, C. J. & Truhlar, D. G. Universal Solvation Model Based on Solute Electron Density and on a Continuum Model of the Solvent Defined by the Bulk Dielectric Constant and Atomic Surface Tensions. *J. Phys. Chem. B* **113**, 6378-6396 (2009).

[31] Pokrovskii, V. A. Calculation of the standard partial molal thermodynamic properties and dissociation constants of aqueous HCl0 and HBr0 at temperatures to 1000°C and pressures to 5 kbar. *Geochim. Cosmochim. Acta* **63**, 1107-1115 (1999).

[32] Haynes, W. M. *CRC Handbook of Chemistry and Physics: A Ready-Reference Book of Chemical and Physical Data*. Boca Raton: CRC Press, (2009).

[33] March, J. *Advanced Organic Chemistry: Reactions, Mechanisms and Structure*, 3rd ed; John Wiley & Sons: Hoboken, NJ, (1985).

[34] Soderberg, T. *Organic Chemistry with Biological Emphasis*, Chemistry Publications, (2016).

[35] Housecroft, C. E. & Sharpe, A. G. *Inorganic Chemistry*, 4th ed.; Pearson Education Limited: Harlow, England, pp. 214 (2012).
