# Supplementary material for: Effects of chloride ions in acid-catalyzed biomass dehydration reactions in polar aprotic solvents
Source: Nat Commun. 2019 Mar 8;10:1132. doi: 10.1038/s41467-019-09090-4 (PMC6408490; doi:10.1038/s41467-019-09090-4)
Supplement: Supplementary file 2 — Peer Review File [file 41467_2019_9090_MOESM2_ESM.pdf]

Reviewers' comments:

Reviewer #1 (Remarks to the Author):

This paper is not ready for detailed evaluation, as the formation of 4-hydroxy-valeric acid (4-HVA) was not considered. It was recently published that gamma-valerolactone (GVL), water, and 4-HVA are in equilibrium in aqueous acidic environment - see Struct. Chem. 2017, 28, 423-429. The GVL and 4-HVA concentrations should be 99% and 1 mol%, respectively. Although 45 mmol GVL was added to the reaction vessel, the reaction mixture actually had 44.5 mmol GVL 0.5 mmol 4-HVA. The latter is about ten times higher than the total chloride concentration, so its role must be considered. A simple 1H-NMR of the reaction mixture after 30 minutes would have been enough to show the presence of 4-HVA.

Reviewer #2 (Remarks to the Author):

General Comments
The authors report experimental and computational work on the effect of metal halides on the dehydration of fructose. They rationalize this effect through a stabilization of the transition state caused by the Cl ion. The results support this hypothesis. The work is potentially interesting but a number of points need to be addressed re the novelty and the mechanism.

1. A major concern regarding the analysis of the experimental data and simulations is the contribution of the acidity to their apparent rate constants and apparent activation energies. Even if the strong acids are completely deprotonated and there is no contribution from general-acid catalysis (for all the studied acids), the rates in the different organic solvents and in water may not be comparable. Different solvents would result in different proton activities which could explain some of the results observed. Even low concentration of salts in organic solvents can alter the proton activity coefficients. I believe it would be important for the impact of this article to address this point.

2. The authors state that 'use of catalytic concentrations (e.g., 5 mM) of chloride salts (e.g., KCl) leads to a 10-fold increase in reactivity'. Later on they state 'In the present study, we observe promotional effects of inorganic salts in polar aprotic solvents at approximately 250- to 1000-fold lower concentrations compared with these chloride effects in water (e.g., 5 mM).' Table 1 indicates that the effect is mainly due to the solvent and the effect of salt is at most a factor of 3. If so, then the text needs to be revised and the novelty of the work is modest.

3. Are the rates still first-order in proton regardless of the solvent or acid?

4. Although the authors pointed out differences in different chloride salts, e.g., NaCl, LiCl, what property of the cation causes change in reactivity, since the principal effect was attributed to the chlorine ion?

5. Regarding the effects in hydrobromic acid why bromine ions are not able to stabilize the transition state as effectively as the chlorine ions?

6. The change in free energies computed is often less than 10 kJ/mol. Given sampling errors and the implicit solvation assumption in the thermodynamic cycle, how statistically relevant are these differences. The authors should quantify the two sources of error.

7. Are the rate constants obtained under differential conditions? In the SI, it appears that they use the entire data set. If not, are the authors accounting for the variation in concentrations of various species?

8. The authors state 'The encapsulated hydrophilic domains localize the protons near the fructose, thus removing the energetic penalties required to transfer the protons from bulk water.' In other parts of the paper, they state that proton transfer is not rate controlling. Are these statements consistent?

Specific Comments:

1. Abstract "Furthermore, increased HMF yields (e.g., >80%)…" : Repetitive

2. Abstract "show that Cl^- acts as a hard base": Is it relevant that chlorine anion is a hard base? Does this property contribute to the stabilization effect of chlorine?

3. Introduction Second Paragraph: Do the authors address the effect on selectivity? The effect of the metal salt on the yield is clear from the experimental data presented in the tables but not the change in the distribution of products.

4. Results Paragraph 1 & Table 1:
a. How were the concentrations of the mineral acids selected for the aqueous and organic solvents (0.5 M to 50 mM in aqueous and organic solvent, respectively)?

b. Why do the authors choose to compare the different acids in terms of concentration rather than pH? The reaction is Brønsted acid catalyzed so a better comparison would be solutions with the same Brønsted acidity. Specifically for the reactions ran in water.

5. Table 2:
a. Why were the HMF yields only calculated for two of the reaction conditions? If selectivity is relevant, reporting selectivity would be valuable. Does the carbon balance close? Do humins form?

b. For the reactions ran with HCl in 90% GVL, the reported yield without any salt was 78 vs. 81 with the added KCl. Is this difference statistically significant? What is the statistical significance of all the reported rate constants and yields? Providing confidence intervals would be useful.

c. Looking at your rate constant in triflic acid and calcium chloride it seems like a significant enhancement in the rate constant, what is the yield? Just by looking at the rate constants calcium chloride seems to have the highest effect.

6. General-acid catalysis by undissociated HCl can be neglected: This effect might be more significant for diprotic acids such as sulfuric acid; did the authors try these calculations with sulfuric or triflic acid?

7. Figure 1A: What is the significance of the shape of the curves? More discussion would be valuable.

Reviewer #3 (Remarks to the Author):

The manuscript entitled "Effects of Chloride Ions in Acid-Catalyzed Biomass Dehydration Reactions in Polar Aprotic Solvents" described a good work about the promotion mechanism of chloride ions on the dehydration of fructose and xylose for the production of HMF and furfural. The HMF and furfural are two important platform compounds in the synthesis of fuels and chemicals. The research topic is very important in biomass conversion. The experiments were well designed. The results are good and convincing. The manuscript was well written. Therefore, I suggest it to be published after some revisions.

My comments are listed below:

1. Table 1. When water, THF/H2O and dioxane/H2O were used as solvents, high HMF yields were achieved under the catalysis of HCl or H2SO4. However, no HMF was obtained using triflic acid as the catalyst. What is the reason for this? Is there any other side reaction (for example the further conversion of HMF to levulinic acid and formic acid) happens under the catalysis of triflic acid? Why high HMF yield was achieved over triflic acid only when 90% GVL/10% H2O was used as solvent? Some comments should be given about this. For the results listed in Table 2, I am wondering why high HMF yields were only achieved over triflic acid + KCl or HCl + KCl catalysts when 90% GVL/10% H2O was used as solvent?

2. In the page 3 of this work, the authors noticed that the presence of the anions I− and F− has some negative effect on the conversion of fructose. Since no HMF was detected when they used HI as catalyst in GVL, the authors think that the iodide ion is strongly electronegative. As the result, it will promote a substitution reaction instead of dehydration. However, the absence of HMF in the product doesn’t mean substitution reaction can occur under the investigated conditions. Did they identified some substituted products? If they have some difficulty in the product analysis, they can also use some simple model reactions (such as the dehydration of alcohol) to prove this.

3. The authors think that the negative effect of F− anion is because HF is weak acid. Therefore, F− ions may compete with fructose for proton, which leads to lower fructose dehydration rates. I am wondering what is the fructose dehydration rate if they use same mole HF to replace KF? By this way, the F- ions will not compete with fructose for the acidic proton. If there is still negative effect when they use HF, they need to find some other reason(s) for the inhibiting effect of F- ions.

4. Page 10. To prevent the ion-exchanging during the reaction, the authors used BTEAC. In fact, the pore sizes of PSA/SiO2 and Amberlyst-70 are still much bigger than the size of benzyltriethylammonium. I think it will be better if they can use zeolites which has smaller pore sizes (for example H-ZSM-5, H-MOR, etc.) as the catalysts.

5. In Table 1 and Table 2, “Triflic” should be corrected as “Triflic acid”.

6. In the page 3 of this manuscript, the authors said “The addition of KCl without a Brønsted acid catalyst in GVL led to a significant decrease in the fructose conversion rate and shows that the chloride anion alone does not catalyze dehydration”. This sentence is misleading. It seems that the presence of KCl has some negative effect on the dehydration of fructose.

7. Page 4, “metal anions” should be “halide (or chloride) anions”.

8. Page 10,Please confirm: “The BTEAC cation” ? Should it be corrected as “The BTEA cation” or “The benzyltriethylammonium cation” because “C” accounts for chloride in the abbreviation.

Reviewer #1 (Remarks to the Author):

This paper is not ready for detailed evaluation, as the formation of 4-hydroxy-valeric acid (4-HVA) was not considered. It was recently published that gamma-valerolactone (GVL), water, and 4-HVA are in equilibrium in aqueous acidic environment - see Struct. Chem. 2017, 28, 423-429. The GVL and 4-HVA concentrations should be 99% and 1 mol%, respectively. Although 45 mmol GVL was added to the reaction vessel, the reaction mixture actually had 44.5 mmol GVL 0.5 mmol 4-HVA. The latter is about ten times higher than the total chloride concentration, so its role must be considered. A simple 1H-NMR of the reaction mixture after 30 minutes would have been enough to show the presence of 4-HVA.

The reviewer brings up a valid criticism. We have carried out new experiments to evaluate the role of 4-HVA for these fructose dehydration reactions in GVL with chloride ions. We have found the presence 4-HVA to be inconsequential in the promotion of the fructose dehydration reaction. We have added additional text and figures regarding our analysis to the manuscript and supplementary information, as follows:

"GVL has previously been shown to reversibly hydrate to 4-hydroxyvaleric acid (4-HVA) under acidic liquid-phase conditions.[33] Quantitative solution-phase ^{1}H NMR was performed to display the effects of chloride ions on the 4-HVA equilibrium concentration in 90% GVL/10% H_2O (w/w) with fructose and H_2SO_4 at various temperatures (Figure S2, A). Equilibrium concentrations of 4-HVA are marginally lower in the presence of KCl at typical reaction conditions (e.g., 1.2 versus 1.4 mol% at 373 K). Additionally, Figure S2, B displays the concentration of 4-HVA as a function of reaction time during fructose dehydration in 90 wt% GVL/10 wt% H_2O (w/w) using H_2SO_4 with and without the presence of KCl. From the initial reaction start-time to ten minutes under reaction conditions (the period during which the reactor achieves the desired temperature of 373 K), the amount of 4-HVA in solution decreases slightly, and it then remains constant throughout the remainder of the experiment, indicating that GVL and 4-HVA are in equilibrium for both cases under typical reaction times and conditions. Moreover, reaction kinetics experiments were performed for fructose dehydration in 90% GVL/10% H_2O containing H_2SO_4, KCl, and valeric acid (a molecular surrogate for 4-HVA at representative equilibrium concentrations of 1 mol% 4-HVA), as shown in Figure S3. Negligible differences in fructose dehydration reaction kinetics were observed with the presence of valeric acid in the reactor. Thus, based on this analysis, we conclude that the increased reaction performance for fructose dehydration with chloride anions in GVL does not involve 4-HVA species."

"Quantitative solution-phase ^{1}H NMR experiments of GVL and 4-HVA (Figure S2), were performed on a Bruker AV 500 MHz spectrometer equipped with an N_2-cooled cryogenic probe. To avoid any confounding effects on the equilibrium conversion of GVL to 4-HVA, samples did not contain deuterated solvents or internal standards. Spectra were collected with the deuterium lock channel off, and shimming was performed manually on the residual water resonance at ~3.5 ppm. Quantitative ^{1}H spectra were collected using a standard 45° pulse sequence with a pulse delay of 10 s, an acquisition time of 2 s, and 8 scans per spectrum. The relative values of the peak integrals at ~0.5 and ~0.7 ppm, corresponding to 4-HVA and GVL, respectively, were used to assess the mole fraction of 4-HVA in solution as a function of temperature and reaction time.

Samples generated under reaction conditions were stored at 277 K overnight. For the variable temperature experiments, samples were allowed to equilibrate for 30 minutes after the sample and probe reached the desired temperature."

"[33] Wong, C. Y. Y. *et al.* Stability of gamma-valerolactone under neutral, acidic, and basic conditions. *Struct. Chem.* **28**, 423-429, (2017)."

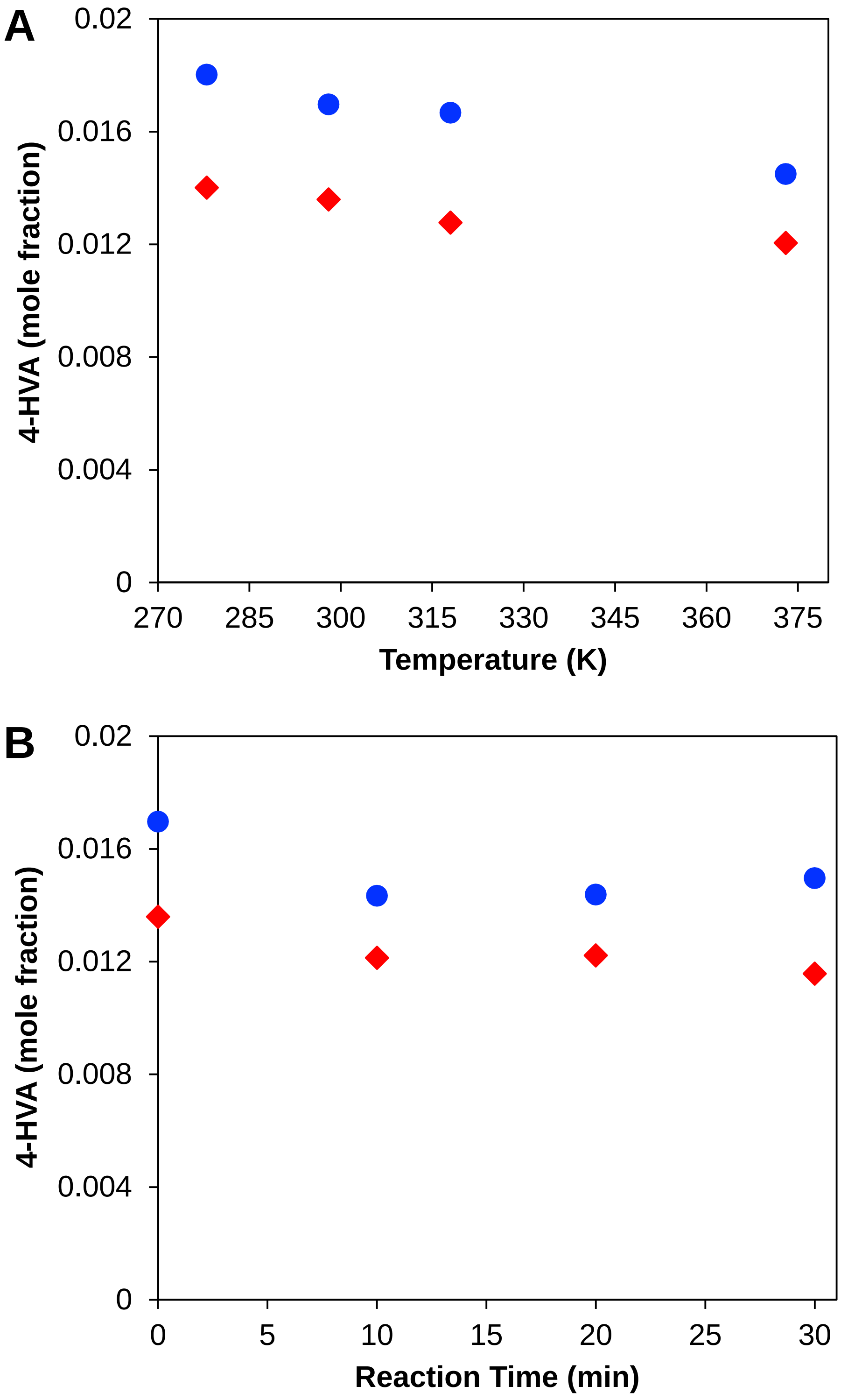

Figure S2. Mole fraction of 4-HVA as function of **(A)** temperature and **(B)** reaction time during the fructose dehydration reaction in 90% GVL/10% H_2O (w/w) with H_2SO_4 (●) and with H_2SO_4 and KCl (◆). Reaction conditions: fructose (50 mM); acid (H_2SO_4; 5 mM); salt (KCl; 5 mM); 373 K for **B**; solvent (5 mL); stirring (700 rpm).

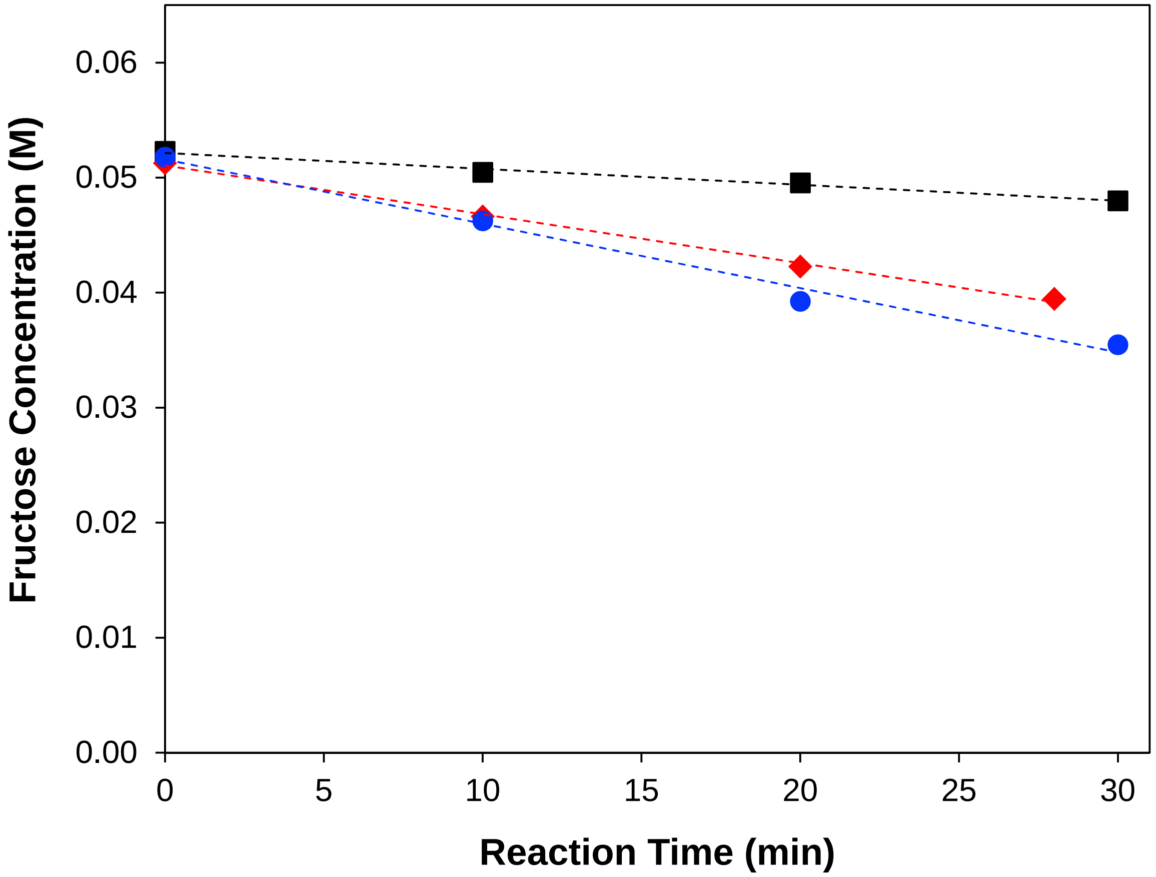

Figure S3. Reaction kinetics data for fructose dehydration in 90% GVL/10% H_2O (w/w) with H_2SO_4 (■); H_2SO_4 and KCl (◆); and H_2SO_4, KCl, and valeric acid (●). Reaction conditions: fructose (50 mM); acid (H_2SO_4; 5 mM); salt (KCl; 5 mM); valeric acid (90 mM); 373 K; solvent (5 mL); stirring (700 rpm).

Reviewer #2 (Remarks to the Author):

General Comments:

The authors report experimental and computational work on the effect of metal halides on the dehydration of fructose. They rationalize this effect through a stabilization of the transition state caused by the Cl ion. The results support this hypothesis. The work is potentially interesting but a number of points need to be addressed re the novelty and the mechanism.

1. A major concern regarding the analysis of the experimental data and simulations is the contribution of the acidity to their apparent rate constants and apparent activation energies. Even if the strong acids are completely deprotonated and there is no contribution from general-acid catalysis (for all the studied acids), the rates in the different organic solvents and in water may not be comparable. Different solvents would result in different proton activities which could explain some of the results observed. Even low concentration of salts in organic solvents can alter the proton activity coefficients. I believe it would be important for the impact of this article to address this point.

The reviewer's overall comment as well as the statement "different solvents would result in different proton activities which could explain some of the results observed" is precisely our position, and thus, we are in alignment with the reviewer. This was, in fact, the basis of our previous study which examined the sole effect of solvent on the kinetics where we showed that the addition of polar aprotic solvents increased the rates by up to three orders of magnitude due to the interactions of the proton as well as the charged transition states with the local solvent environment. The present manuscript shows that changing the acid and its corresponding anion can further increase the rates.

We outlined this hypothesis thoroughly in our previous work as well as in the following sections of the Manuscript and Supplementary Information:

"Previously, we suggested that increased reactivity in polar aprotic solvents, such as GVL, for acid-catalyzed reactions is due to destabilization of the acidic proton, where the protonated transition state is destabilized by a fraction of this amount in these polar aprotic solvents relative to water.[5] More recently, we quantified these solvation effects in terms of initial and transition state contributions[37,40] and developed a multi-descriptor correlation model that predicts rate constants as a function of solvent composition[41] for acid-catalyzed reactions. Based on the experimental results of the present study, we now probe whether the observed increases in acid-catalyzed dehydration rates with chloride salts in polar aprotic solvents are due to the stabilization of the protonated transition state by chloride ions."

The simulations as well as the experiments calculate and measure the apparent free energy barriers, respectively. As such, they explicitly include the changes in the proton and reactant activities in their determination of the apparent free energy barriers. We have added the following text to the Supplementary Information:

“The relationship between the proton activity coefficient and the reactivity can be established by writing the rate in terms of the activities which are equal to the activity coefficients multiplied by the concentrations of the reactants.

$$r = k_{H^+}\gamma_{H^+}\gamma_R[H^+][R]$$

where r is an experimentally measured rate, k_{H+} is a rate constant for specific-acid catalyzed and γ_{H+} and γ_R are the activity coefficient of proton and fructose, respectively. Transition state theory can then be used to express the rate constants in term of activation free energy:

$$r = \kappa\frac{k_BT}{h}e^{\frac{-\Delta G^\ddagger}{RT}}\gamma_{H^+}\gamma_R[H^+][R]$$

where κ is a transmission coefficient, k_B is Boltzmann constant, T is temperature, and h is Planck constant. The activity coefficients γ_{H+} and γ_R can then be combined with the activation free energy term:

$$r = \kappa\frac{k_BT}{h}e^{\frac{\ln(\gamma_{H^+})RT+\ln(\gamma_R)RT-\Delta G^\ddagger}{RT}}[H^+][R]$$

to express rate in terms of an apparent free energy barrier:

$$r = \kappa\frac{k_BT}{h}e^{\frac{\Delta G_{calculated}{}^\ddagger}{RT}}[H^+][R]$$

As such the experimental as well as the calculated activation free energies reported in the manuscript due include these changes in proton activity. The simulations model the proton and transition state interactions with the explicit solvent molecules in the local environment and as such directly calculate the explicit changes in proton activities.”

2. The authors state that ‘use of catalytic concentrations (e.g., 5 mM) of chloride salts (e.g., KCl) leads to a 10-fold increase in reactivity’. Later on they state ‘In the present study, we observe promotional effects of inorganic salts in polar aprotic solvents at approximately 250- to 1000-fold lower concentrations compared with these chloride effects in water (e.g., 5 mM).’ Table 1 indicates that the effect is mainly due to the solvent and the effect of salt is at most a factor of 3. If so, then the text needs to be revised and the novelty of the work is modest.

The statement: “use of catalytic concentrations (e.g., 5 mM) of chloride salts (e.g., KCl) leads to a 10-fold increase in reactivity” can be observed and verified from the results for the fructose dehydration reaction in Table 1 in 90% dioxane by comparing the reactivity values between H_2SO_4 and HCl catalysts, i.e., 10 M^{-1} ks^{-1} using H_2SO_4 vs. 95 M^{-1} ks^{-1} using HCl.

The following statement in the text is supported by the results from the fructose dehydration reaction in Figure 2: “In the present study, we observe promotional effects of inorganic salts in polar aprotic solvents at approximately 250- to 1000-fold lower concentrations compared with these chloride effects in water (e.g., 5 mM). For example, in pure H_2O, 2.5 M KCl is required to achieve a promotional effect of a factor of 3, whereas in 90% GVL, 0.005 M KCl is required to achieve the same promotional factor of 3. Thus, comparing the H_2O and 90% GVL solvents, a 500-fold lower concentration of KCl is required to achieve similar promotional effects (factor of 3) in 90% GVL relative to H_2O.

3. Are the rates still first-order in proton regardless of the solvent or acid?

We have explored the solvent kinetic isotope effect with D_2O, and also using GVL mixtures with D_2O as solvent, using both H_2SO_4 and HCl as catalysts for fructose dehydration, as shown in Table S2. These measurements of the KIE led to rate constant value ratios (k_D/k_H) of 1.7−2.7, suggesting that general-acid catalysis for fructose dehydration using both H_2SO_4 and HCl in both H_2O and GVL can also be neglected.[34,35] Thus, the reaction rate will be proportional to the dissociated proton concentration for H_2O and GVL with H_2SO_4 and HCl.

[34] Carey, F. A. & Sundberg, R. J. in *Advanced Organic Chemistry – Part A: Structure and Mechanisms*, Springer, New York, NY, USA, pp. 346-347 (2007).

[35] Willi, A. V. in *Homogeneous Catalysis of Organic Reactions (Mainly Acid-Base)* in *Comprehensive Chemical Kinetics, Vol. 8* (Eds.: Bamford, C. H. & Tipper, C. F.), Elsevier, Amsterdam, The Netherlands, pp. 21-24 (1997).

4. Although the authors pointed out differences in different chloride salts, e.g., NaCl, LiCl, what property of the cation causes change in reactivity, since the principal effect was attributed to the chlorine ion?

We do observe that there are slight differences in fructose dehydration reactivity with various cations; however, we show that the increased reaction performance is primarily attributed to the effect of the chloride anion. While we are interested in the effects of other cations and anions, the scope of this manuscript was focused on elucidating the effects of the chloride anion. Further work exploring the effects of additional cations and anions would be appropriate for a follow-up article. Furthermore, we have now stated in the manuscript: "The addition of the cations Na^+, Li^+, Ca^{2+} and the anion Br^- with H_2SO_4 and/or triflic acid did not lead to significant differences in fructose conversion reaction rates."

5. Regarding the effects in hydrobromic acid why bromine ions are not able to stabilize the transition state as effectively as the chlorine ions?

This is an insightful question. We believe that changes in reactivity are due to the changes in basicity of the anion.

The acidity and pK_a of the different acids examined follow the order of:

$$HCl < HBr < HI < H_2SO_4 < \text{Triflic Acid}$$

Cl^- has the highest proton affinity and is therefore the strongest conjugate base.

The chloride ion which has the smallest ionic radii and as such the highest localized negative charge is the most basic anion examined in this work. The small radii and localized negative

charge allow it to interact more strongly with positively charged transition state (see Fig. S10, C), thus leading to lower activation free energy.

Anions with larger ionic radius such as bromide delocalize their negative charge over a larger region of space and therefore interact more weakly with charged transition states. This weak interaction is also observed for other large anions (Fig. S13). As such, bromide, iodide, bisulfate, and triflate ions are less effective at stabilizing the transition state than chloride ion. This is consistent with the observed experimental results.

6. The change in free energies computed is often less than 10 kJ/mol. Given sampling errors and the implicit solvation assumption in the thermodynamic cycle, how statistically relevant are these differences. The authors should quantify the two sources of error

We are exploring herein the ***changes*** that result to the reaction energies or activation barriers, i.e. $\Delta(\Delta G)$. The errors which examining the changes in free energy difference are typically much smaller than those associated with the quantitative free energies of reaction or free energy barriers as there is a cancellation of errors or biases that occur in the system. The changes in barrier or reaction energies are often within 3 kJ mol^{-1}.

In order to minimize the sampling errors and establish the lowest energy states, we carried out extensive equilibration runs using classical force field simulations. The subsequent *ab initio* MD simulations were then carried out with well-established sampling methods to minimize any errors from sampling. The AIMD simulations were also run with different low energy starting configuration and then averaged. Calculated energy differences for different low energy starting structures were calculated to be less than 2 kJ mol^{-1}. We did not observe any drifts in relevant observables during the data collection. In addition, this technique was successfully used to calculate activation free energy in similar systems.[37]

All of the simulations were carried out in the presence of explicit solvent molecules and as such there are no errors associated with implicit solvation. The implicit solvation simulations were only used to calculate the stronger acidity of H_2SO_4 in comparison with HCl, as shown in Table S4. The model accurately predicts pK_a values for HCl and other acids.

We have added the following to the manuscript to help address the reviewers point.

"Extensive molecular dynamic simulations were carried out to establish the lowest free energy states. Subsequent AIMD simulations were carried out with established sampling methods. Errors in the calculated changes in the reaction energies and activation barriers were found to be within 2 kJ mol^{-1}."

7. Are the rate constants obtained under differential conditions? In the SI, it appears that they use the entire data set. If not, are the authors accounting for the variation in concentrations of various species?

The reviewer is correct that we use the entire dataset to derive rate constants, and the rate constants are not obtained under differential conditions. Thus, we report in the manuscript rate constants for fructose conversion, accounting for variation in concentrations of various species.

8. The authors state 'The encapsulated hydrophilic domains localize the protons near the fructose, thus removing the energetic penalties required to transfer the protons from bulk water.' In other parts of the paper, they state that proton transfer is not rate controlling. Are these statements consistent?

The two statements the reviewer refers are for two different mechanisms. The first statement:

"These measurements of the KIE led to rate constant value ratios (k_D/k_H) of 1.7−2.7. The measured KIE suggests acid-catalyzed dehydration of fructose is catalyzed primarily by the acidic proton, H^+, (i.e., reactant protonation is not rate determining), and therefore, general-acid catalysis (k_{HB}) can be neglected.[33,34]"

is misstated as it refers to the proton transfer from acid to a solvent molecule (i.e. acid dissociation). For the acids examined in this work, the acid readily dissociates in solution and is rapidly equilibrated.

The second statement:

"The encapsulated hydrophilic domains localize the protons near the fructose, thus removing the energetic penalties required to transfer the protons from bulk water."

refers to the actual transfer of a free proton in bulk water to the hydrophilic domain near fructose (tied to proton activity). This step can be rate limiting.

To avoid any further misunderstanding, we now refer to the first form of transfer as "acid dissociation" and have changed the first statement to:

'These measurements of the KIE led to rate constant value ratios (k_D/k_H) of 1.7−2.7. The measured KIE suggests acid-catalyzed dehydration of fructose is catalyzed primarily by the acidic proton, H^+, (i.e., acid dissociation is not rate determining), and therefore, general-acid catalysis (k_{HB}) can be neglected.[34,35]"

Specific Comments:

1. Abstract "Furthermore, increased HMF yields (e.g., >80%)…" : Repetitive

We have removed the phrase "(e.g., >80%)" from this statement in the abstract. This sentence in the abstract is now as follows: "Furthermore, increased HMF yields can be achieved using polar aprotic solvents mixed with chlorides."

2. Abstract "show that Cl^- acts as a hard base": Is it relevant that chlorine anion is a hard base? Does this property contribute to the stabilization effect of chlorine?

The reviewer raises an interesting question. We believe that the hard basicity of Cl^- is important in understanding the differences of different anions. Since Cl^- is a hard base, the negative charge is more localized as its radius is significantly smaller. As such it can come closer to positive charge in the transition state in the same way it stabilizes a free proton. It is more basic, and as such, it can better stabilize the positively charged transition state. This was the important point raised in question 2 as to why bromine ions are not able to stabilize the transition state as effectively as the chlorine ions?

In order to avoid confusion, we have modified the sentence:

"*Ab initio* molecular dynamics simulations (AIMD) show that highly localized negative charge on Cl^- (hard basicity) allows the chloride anion to more readily approach and stabilize the oxocarbenium ion that forms and the deprotonation transition state."

3. Introduction Second Paragraph: Do the authors address the effect on selectivity? The effect of the metal salt on the yield is clear from the experimental data presented in the tables but not the change in the distribution of products.

The reviewer brings up a valid point. In order to avoid confusion to the reader, we have removed the phrase "selectivity" and replaced the phrase with "yield" in the second paragraph of the introduction. The sentence is now as follows: "Herein, we show that the addition of catalytic concentrations (e.g., < 5 mM) of inorganic salts, specifically chloride salts, in polar aprotic solvents, such as GVL, can further enhance reactivity and yield for acid-catalyzed reactions related to biomass conversion."

Furthermore, we also removed the phrase "selectivity" and placed the phrase with "yield" in the following sentence: "Importantly, we found the increased reactivity and yield with the addition of chloride ions in GVL is also observed for other acid-catalyzed sugar dehydration reactions, and this behavior thus appears to be of general significance (Table S1)."

4. Results Paragraph 1 & Table 1:

a. How were the concentrations of the mineral acids selected for the aqueous and organic solvents (0.5 M to 50 mM in aqueous and organic solvent, respectively)?

b. Why do the authors choose to compare the different acids in terms of concentration rather than pH? The reaction is Brønsted acid catalyzed so a better comparison would be solutions with the same Brønsted acidity. Specifically for the reactions ran in water.

The reactivity values for fructose dehydration between H_2O and a polar aprotic solvent, such as 90% GVL, are significantly different. For example, fructose dehydration kinetics are increased by roughly 2-orders of magnitude (as shown in Table 1) in 90% GVL compared to using pure H_2O as solvent. Thus, in order to keep reaction kinetics experiments on similar experimental timelines, concentrations of mineral acids for aqueous solvent systems were increased by 2-orders of magnitude, from 50 mM to 0.5 M.

We agree that an ideal comparison would be solutions with similar Brønsted acidities. However, we compare acids by concentration rather than by pH, since pK_a values and relative acidity scales for acids in non-aqueous solvents, such as GVL, THF, and dioxane, are extremely limited at present. For the reactions performed in water, HCl, H_2SO_4, and triflic acid are strong acids (pK_a values < -3), and thus, these acids are effectively fully dissociated in water at the reaction conditions (i.e., pH < 1). Furthermore, in order to avoid confusion to the reader, we opted not to switch between acid concentration and solution pH between aqueous and organic solvents.

5. Table 2:

a. Why were the HMF yields only calculated for two of the reaction conditions? If selectivity is relevant, reporting selectivity would be valuable. Does the carbon balance close? Do humins form?

b. For the reactions ran with HCl in 90% GVL, the reported yield without any salt was 78 vs. 81 with the added KCl. Is this difference statistically significant? What is the statistical significance of all the reported rate constants and yields? Providing confidence intervals would be useful.

c. Looking at your rate constant in triflic acid and calcium chloride it seems like a significant enhancement in the rate constant, what is the yield? Just by looking at the rate constants calcium chloride seems to have the highest effect.

To avoid confusion to the reader, the HMF yield value column from Table 2 was removed from the table, since only two HMF yield values were present in the table. These two yield values have been added to the Table 2 footnotes: ". . . [e] 78 ±1% HMF yield at 393 K at approximately 90% conversion. [f] 81 ±1% HMF yield at 393 K at approximately 90% conversion."

Furthermore, we thank the reviewer for the valuable suggestion. We added 95% confidence intervals for the rate constants and HMF yield values in Table 2 and Table 3 of the manuscript.

6. General-acid catalysis by undissociated HCl can be neglected: This effect might be more significant for diprotic acids such as sulfuric acid; did the authors try these calculations with sulfuric or triflic acid?

Indeed, we have also explored the solvent kinetic isotope effect with D_2O using sulfuric acid for fructose dehydration, as shown in Table S2. These measurements of the KIE using sulfuric acid led to rate constant value ratios (k_D/k_H) of 1.7−1.9, suggesting that general-acid catalysis for fructose dehydration using sulfuric acid can also be neglected.[34,35]

[34] Carey, F. A. & Sundberg, R. J. in *Advanced Organic Chemistry – Part A: Structure and Mechanisms*, Springer, New York, NY, USA, pp. 346-347 (2007).

[35] Willi, A. V. in *Homogeneous Catalysis of Organic Reactions (Mainly Acid-Base)* in *Comprehensive Chemical Kinetics, Vol. 8* (Eds.: Bamford, C. H. & Tipper, C. F.), Elsevier, Amsterdam, The Netherlands, pp. 21-24 (1997).

7. Figure 1A: What is the significance of the shape of the curves? More discussion would be valuable.

In order to add clarity to the reader, we expanded the discussion of Figure 1A in the manuscript. The discussion is now as follows: "Figure 1A displays the results for the effects of varying the chloride ion concentration on the fructose conversion rate constant value using triflic acid as catalyst in 90% GVL/10% H_2O. The reaction rate constant shows an increasing, concave down reactivity trend with increasing chloride ion concentration, meaning there is a diminishing promotional effect using chloride ions."

Reviewer #3 (Remarks to the Author):

The manuscript entitled "Effects of Chloride Ions in Acid-Catalyzed Biomass Dehydration Reactions in Polar Aprotic Solvents" described a good work about the promotion mechanism of chloride ions on the dehydration of fructose and xylose for the production of HMF and furfural. The HMF and furfural are two important platform compounds in the synthesis of fuels and chemicals. The research topic is very important in biomass conversion. The experiments were well designed. The results are good and convincing. The manuscript was well written. Therefore, I suggest it to be published after some revisions.

My comments are listed below:

1. Table 1. When water, THF/H2O and dioxane/H2O were used as solvents, high HMF yields were achieved under the catalysis of HCl or H2SO4. However, no HMF was obtained using triflic acid as the catalyst. What is the reason for this? Is there any other side reaction (for example the further conversion of HMF to levulinic acid and formic acid) happens under the catalysis of triflic acid? Why high HMF yield was achieved over triflic acid only when 90% GVL/10% H2O was used as solvent? Some comments should be given about this. For the results

listed in Table 2, I am wondering why high HMF yields were only achieved over triflic acid + KCl or HCl + KCl catalysts when 90% GVL/10% H2O was used as solvent?

The en-dashes in Table 1 under the HMF yield column represented reactions that were not performed since we considered these data to be redundant as we have results to support that the use of sulfuric acid and triflic acid as catalysts gives similar reaction kinetics and HMF yield values for fructose dehydration. However, in order to avoid confusion, these reactions were performed and corresponding HMF yield values were added to Table 1 to "complete" the table.

Furthermore, for Table 2, also to avoid confusion, the HMF yield value column was removed from the table, since only two HMF yield values were present in the table. These two yield values have been added to the Table 2 footnotes: ". . . e] 78 ±1% HMF yield at 393 K at approximately 90% conversion. [f] 81 ±1% HMF yield at 393 K at approximately 90% conversion."

2. In the page 3 of this work, the authors noticed that the presence of the anions I− and F− has some negative effect on the conversion of fructose. Since no HMF was detected when they used HI as catalyst in GVL, the authors think that the iodide ion is strongly electronegative. As the result, it will promote a substitution reaction instead of dehydration. However, the absence of HMF in the product doesn't mean substitution reaction can occur under the investigated conditions. Did they identified some substituted products? If they have some difficulty in the product analysis, they can also use some simple model reactions (such as the dehydration of alcohol) to prove this.

The reviewer brings up a valid criticism. We have altered the text to highlight our speculation for the observed result of the low fructose dehydration reaction rate and inhibited HMF production for reactions containing iodide. We have also included the oxidation of the iodide anion as another potential explanation of the observed reaction behavior.

The text of that section is now as follows: "The iodide ion is strongly electronegative and may potentially promote a substitution reaction mechanism over an elimination mechanism, as no detectable production of HMF was observed using HI as catalyst in GVL. Furthermore, iodide anions are strong reducing agents and have the potential to oxidize to diatomic iodine and water, deactivating the acid catalyst."

3. The authors think that the negative effect of F− anion is because HF is weak acid. Therefore, F− ions may compete with fructose for proton, which leads to lower fructose dehydration rates. I am wondering what is the fructose dehydration rate if they use same mole HF to replace KF? By this way, the F- ions will not compete with fructose for the acidic proton. If there is still negative effect when they use HF, they need to find some other reason(s) for the inhibiting effect of F- ions.

A Triflic Acid/HF system compared to a Triflic Acid/KF system will lead to marginally improved dehydration reaction rates, as the Triflic Acid/HF system will contain more acidic

protons to catalyze dehydration. However, the Triflic Acid/HF system will also contain lower concentrations of fluoride anions due to the higher concentration of undissociated HF. Thus, due to the inherent nature of HF being a weak acid (pK_a value of 3.2 in H_2O), observing the effects of strictly the fluoride anion in acid-catalyzed dehydration reactions is extremely difficult.

These limitations with assessing the role of fluoride anions in acid-catalyzed biomass dehydration reactions were also noted in similar work that is cited in the manuscript:

[29] Enslow, K. R. & Bell, A. T. The Role of Metal Halides in Enhancing the Dehydration of Xylose to Furfural. *ChemCatChem* **7**, 479-489 (2015).

4. Page 10. To prevent the ion-exchanging during the reaction, the authors used BTEAC. In fact, the pore sizes of PSA/SiO2 and Amberlyst-70 are still much bigger than the size of benzyltriethylammonium. I think it will be better if they can use zeolites which has smaller pore sizes (for example H-ZSM-5, H-MOR, etc.) as the catalysts.

The reviewer is correct in stating that the pore sizes of PSA/SiO_2 and Amberlyst 70 are bigger than the molecular size of the benzyltriethylammonium. Indeed, we have considered using zeolites to assess the chloride effect for fructose dehydration in GVL using solid acid catalysts; however, we wanted to ensure the absence of mass transport limitations for fructose dehydration and obtain reliable reaction kinetics data in order to draw strong conclusions regarding chloride effects in solid acid catalysts. Based on our reaction kinetics results using PSA/SiO_2 and Amberlyst 70, we believe that benzyltriethylammonium was able to provide sufficient steric hinderance to prevent ion-exchange with the solid acid catalysts. The authors appreciate the insightful comment.

5. In Table 1 and Table 2, "Triflic" should be corrected as "Triflic acid".

In order to avoid confusion to the reader, the phrase "Triflic" has now been replaced with the phrase "Triflic Acid" in both Table 1 and in Table 2.

6. In the page 3 of this manuscript, the authors said "The addition of KCl without a Brønsted acid catalyst in GVL led to a significant decrease in the fructose conversion rate and shows that the chloride anion alone does not catalyze dehydration". This sentence is misleading. It seems that the presence of KCl has some negative effect on the dehydration of fructose.

In order to avoid confusion to the reader, we have removed the sentence "The addition of KCl without a Brønsted acid catalyst in GVL led to a significant decrease in the fructose conversion rate and shows that the chloride anion alone does not catalyze dehydration" and replaced the sentence with the following: "The use of KCl for fructose dehydration without a Brønsted acid catalyst in GVL led to a low fructose conversion rate and shows that the chloride anion alone does not catalyze fructose dehydration."

7. Page 4, "metal anions" should be "halide (or chloride) anions".

In order to avoid confusion to the reader, we have removed the phrases "metal cations" and "metal anions" and replaced the phrases with "metal halide cations" and "metal halide anions". The sentence is now as follows: "Similarly, Enslow, et al. explored the role of various metal halides for xylose dehydration to furfural using salt cations (i.e., Li^+, Na^+, and K^+) and anions (i.e., Cl^-, Br^-, and I^-) at 5 M metal halide aqueous solutions, suggesting that metal halide cations disrupt the solvation of xylose by water, and metal halide anions act to stabilize critical xylose dehydration intermediates, leading to increased reactivity and selectivity.[29]

8. Page 10,Please confirm: "The BTEAC cation" ? Should it be corrected as "The BTEA cation" or "The benzyltriethylammonium cation" because "C" accounts for chloride in the abbreviation.

In order to avoid confusion to the reader, we have removed the phrase "The BTEAC cation" and replaced the phrase with "The benzyltriethylammonium cation". The sentence is now as follows: "The benzyltriethylammonium cation was used to provide sufficient steric hindrance to prevent cation exchange with the acidic protons of the solid catalysts."

REVIEWERS' COMMENTS:

Reviewer #2 (Remarks to the Author):

The paper has been adequately revised and is now suitable for publication in NatComm.

Reviewer #3 (Remarks to the Author):

The authors have properly answered the reviewers'questiones and carefully revised their manuscript. I recommend this paper to be published as it is.
